# Supplementary material for: Photoswitchable dynamic conjugate addition-elimination reactions as a tool for light-mediated click and clip chemistry
Source: Nat Commun. 2023 Jul 7;14:4015. doi: 10.1038/s41467-023-39669-x (PMC10328932; doi:10.1038/s41467-023-39669-x)
Supplement: Supplementary file 1 — Supplementary Information [file 41467_2023_39669_MOESM1_ESM.pdf]

Supplementary Information

Photoswitchable Dynamic Conjugate Addition-Elimination Reactions as a Tool for Light-Mediated Click and Clip Chemistry

Hanwei Lu[1,2], Hebo Ye[1], Meilan Zhang,[1] Zimu Liu,[1] Hanxun Zou,[1] and Lei You[1,2,3*]

[1]State Key Laboratory of Structural Chemistry, Fujian Institute of Research on the Structure of Matter, Chinese Academy of Sciences, Fuzhou 350002, China.

[2]University of Chinese Academy of Sciences, Beijing 100049, China.

[3]Fujian Science & Technology Innovation Laboratory for Optoelectronic Information of China, Fuzhou 350108, China.

[*]e-mail: lyou@fjirsm.ac.cn

Table of content

1. Synthesis and Characterization (Supplementary Figures 1-28)

2. Photoswitching Experiments (Supplementary Figures 29-31)

3. Light-Controlled Exchange of Alcohol Nucleophiles (Supplementary Figures 32-34)

4. Light-Controlled Exchange of Thiol Nucleophiles (Supplementary Figures 35-56)

5. Light-Controlled Exchange of Amine Nucleophiles (Supplementary Figures 57-104)

6. Determination of the Activation Energy (Supplementary Figures 105-107)

7. Computations (Supplementary Figures 108-109)

8. Light-Controlled Amphiphilic Assemblies (Supplementary Figures 110-114)

9. Light-Control Regulation of Polymers (Supplementary Figures 115-129)

10. Supplementary References

1. Synthesis and Characterization

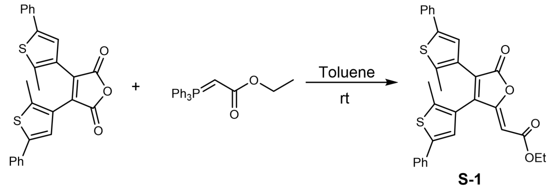

Supplementary Figure 1. Synthesis of **S-1**.

To a solution of 3,4-bis(2-methyl-5-phenylthiophene-3-yl)furan-2,5-dione (prepared according to the literature procedure,[1] 0.50 g, 1.13 mmol) in anhydrous toluene (10 mL) was added (carbethoxymethylene)triphenylphosphorane (0.78 g, 2.26 mmol). The mixture was stirred overnight at room temperature. After quenched with acetone the solution was concentrated under reduced pressure, and the residue was purified by silica gel column chromatography (ethyl acetate/petroleum ether, 1:10) to afford the product **S-1** as a green solid (0.47 g, 85%). ^{1}H NMR ($CDCl_3$): δ = 7.60 (d, *J* = 7.2 Hz, 2H), 7.51 (d, *J* = 7.6 Hz, 2H), 7.44 (t, *J* = 7.2 Hz, 2H), 7.36-7.27 (m, 4H), 7.19 (d, *J* = 8.2 Hz, 2H), 5.6 (s, 1H), 4.33 (q, *J* = 6.8 Hz, 2H), 2.14 (s, 3H), 2.06 (s, 3H), 1.38 (t, *J* = 7.2 Hz, 3H). ^{13}C{^{1}H} NMR ($CDCl_3$): δ = 167.1, 163.6, 156.7, 144.8, 142.9, 141.6, 140.9, 140.1, 133.5, 133.2, 129.1, 128.9, 128.1, 127.7, 127.2, 127.0, 126.9, 125.7, 125.6, 123.6, 123.1, 100.7, 61.2, 14.9, 14.4, 14.3. ESI-HRMS: *m/z* Calcd. for $C_{30}H_{24}O_4S_2Na$ $[M + Na]^+$: 535.1014; found:535.1009.

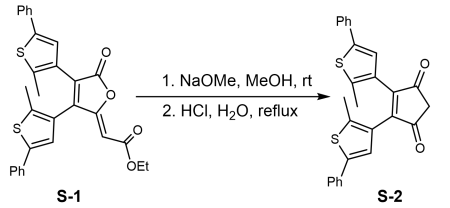

Supplementary Figure 2. Synthesis of **S-2**.

S-1 (0.38 g, 0.78 mmol) was dissolved in anhydrous MeOH (25 mL), and 0.3 mL of MeONa (5.4 M in methanol, 1.57 mmol) was added dropwise over 10 min. The mixture was stirred for 0.5 h at room temperature, and then the solvent was removed under reduced pressure. The obtained solid was suspended in 2 M HCl (10 mL). After refluxing for 1 h the mixture was cooled to room temperature and extracted with ethyl acetate (3 × 20 mL). The combined organic layers were washed with brine and dried over Na_2SO_4, and the solvents were evaporated *in vacuo*. The residue was purified by silica gel column chromatography (ethyl acetate/petroleum ether, 1:6) to give the product **S-2** as a yellow solid (0.24 g, 70%). ^{1}H NMR (DMSO-d_6): δ = 7.55 (d, *J* = 7.2 Hz, 4H), 7.41 (t, *J* = 7.2 Hz, 4H), 7.33-7.28 (m, 4H), 3.31 (s, 2H), 2.01 (s, 6H). ^{13}C{^{1}H} NMR (DMSO-d_6): δ = 199.6, 150.3, 140.4, 140.3, 133.5, 129.7, 129.2, 128.2, 125.5, 124.9, 42.4, 14.9. ESI-HRMS: *m/z* Calcd. for $C_{27}H_{20}O_2S_2Na$ $[M + Na]^+$: 463.0802; found: 463.0801.

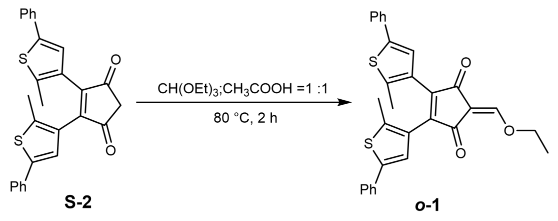

Supplementary Figure 3. Synthesis of ***o*-1**.

S-2 (0.24 g, 0.55 mmol) was dissolved in the mixture solution of triethyl orthoformate (5 mL) and acetic acid (5 mL), After stirring for 2 h under 80 °C the reaction was quenched with water and extracted with ethyl acetate (3 × 20 mL). The combined organic layers were washed with brine (3 × 20 mL), dried over Na_2SO_4, and evaporated *in vacuo*. The residue was purified by silica gel column chromatography (ethyl acetate/petroleum ether, 1:2) to give the product ***o*-1** as a green solid (0.19 g, 72%). ^{1}H NMR (CD_3CN): δ = 7.62 (s, 1H), 7.56-7.59 (m, 4H), 7.40 (t, *J* = 7.6 Hz, 4H), 7.32 (t, *J* = 6.8 Hz, 2H), 7.25 (d, *J* = 7.2 Hz, 2H), 4.45 (q, *J* = 7.2 Hz, 2H), 2.06 (s, 6H), 1.43 (t, *J* = 7.2 Hz, 3H). ^{13}C{^{1}H} NMR (CD_3CN): δ = 192.3, 189.5, 160.5, 146.5, 145.0, 140.6, 140.5, 133.7, 129.1, 127.1, 125.3, 125.0, 124.9, 107.6, 74.3, 14.8, 14.1, 14.1. ESI-HRMS: *m/z* Calcd. for $C_{30}H_{24}O_3S_2Na$ [M + Na]$^+$: 519.1065; found: 519.1059.

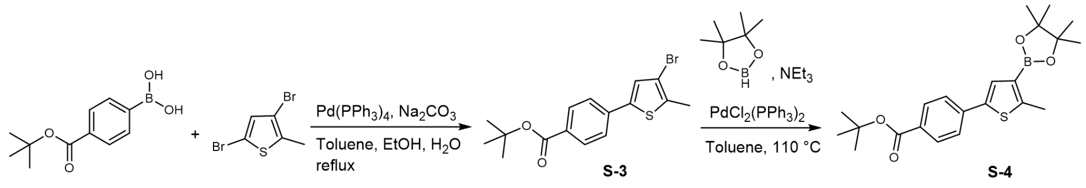

Supplementary Figure 4. Synthesis of **S-4**

3,5-Dibromo-2-methylthiophene (prepared according to the literature procedure,[2] 3.53 g, 13.90 mmol) was dissolved in a mixture of 10 mL toluene and 5 mL of 2 M Na_2CO_3 aqueous solution. After stirring for 10 min at room temperature, the solution of 4-(*tert*-butoxycarbonyl)phenylboronic acid (3.97 g, 16.68 mmol) in EtOH (5 mL) was added, then $Pd(PPh_3)_4$ (0.27 g, 0.24 mmol) was added in portion, and the reaction was heated at 90 °C for 8 h. The mixture was quenched with 50 mL of water and extracted with ethyl acetate (3 × 50 mL). The combined organic layers were washed with brine (3 × 20 mL), dried over Na_2SO_4, and evaporated *in vacuo*. The residue was purified by silica gel column chromatography (ethyl acetate/petroleum ether, 1:50) to give the product **S-3** as a white solid (3.47 g, 71%). ^{1}H NMR ($CDCl_3$): δ = 7.99 (d, *J* = 7.2 Hz, 2H), 7.56 (d, *J* = 7.2 Hz, 2H), 7.21 (s, 1H), 2.45 (s, 3H), 1.63 (s, 9H), ^{13}C{^{1}H} NMR ($CDCl_3$): δ = 165.3, 139.9, 137.1, 135.1, 131.0, 130.2, 126.8, 124.8, 110.3, 81.1, 28.2, 15.0. ESI-HRMS: *m/z* Calcd. for $C_{16}H_{18}BrO_2S_2$ [M + H]$^+$: 353.0211; found: 353.0207.

S-3 (3.06 g, 8.70 mmol) was combined with pinacolborane (3.8 mL, 26.10 mmol),

NEt_3 (2.0 mL, 14.44 mmol), $PdCl_2(PPh_3)_2$ (0.11 g, 0.15 mmol), and 15 mL degassed toluene. The reaction mixture was refluxed for 4 h. The mixture was quenched with 50 mL of water and extracted with ethyl acetate (3 × 50 mL). The organic layers were dried over Na_2SO_4, evaporated, and then purified by silica gel column chromatography (ethyl acetate/petroleum ether, 1:30) to give the product **S-4** (2.61 g, 75%) as a white solid. ^1H NMR ($CDCl_3$): δ = 7.98 (d, J = 7.2 Hz, 2H), 7.62 (d, J = 7.2 Hz, 2H), 7.57 (s, 1H), 2.73 (s, 3H), 1.62 (s, 9H), 1.34 (s, 12H). $^{13}C\{^1H\}$ NMR ($CDCl_3$): δ= 165.4, 153.7, 139.7, 138.3, 130.4, 130.1, 130.0, 125.0, 125.0, 83.4, 80.7, 28.2, 24.9, 16.0. ESI-HRMS: m/z calcd for $C_{22}H_{30}BO_4S$ $[M + H]^+$: 401.1958; found: 401.1956.

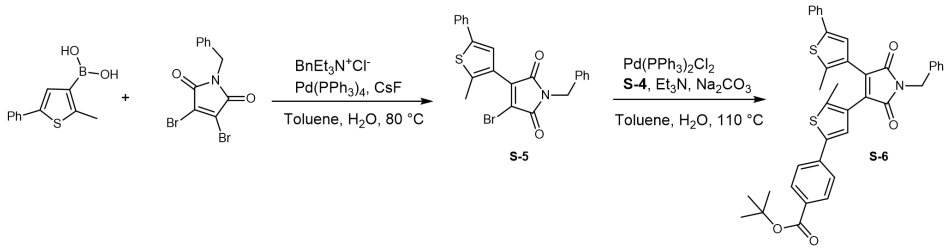

Supplementary Figure 5. Synthesis of **S-6**.

(2-Methyl-5-phenylthiophen-3-yl)boronic acid (prepared according to the literature procedure,[3] 1.04 g, 4.78 mmol) was combined with 2,3-dibromo-*N*-benzylmaleimide (prepared according to the literature procedure,[4] 2.45 g, 7.17 mmol), cesium fluoride (0.36 g, 2.39 mmol), $Pd(PPh_3)_4$ (0.06 g, 0.05 mmol), and 15 mL each of degassed toluene and water. The reaction mixture was refluxed for 8 h, quenched with 50 mL of water, and extracted with ethyl acetate (3 × 50 mL). The organic layers were dried over Na_2SO_4, evaporated, and then purified by silica gel column chromatography (ethyl acetate/petroleum ether, 1:30) to give the product **S-5** (1.67 g, 80%) as a yellow solid. ^1H NMR ($CDCl_3$): δ = 7.57 (d, J = 7.2 Hz, 2H), 7.46 (d, J = 7.2 Hz, 2H), 7.42-7.36 (m, 4H), 7.35-7.31 (m, 2H), 7.29 (s, 1H), 4.81 (s, 2H), 2.54 (s, 3H). $^{13}C\{^1H\}$ NMR ($CDCl_3$): δ = 168.2, 165.3, 142.3, 141.6, 138.6, 135.8, 133.5, 129.0, 128.8, 128.1, 127.8, 125.7, 125.5, 123.8, 123.7, 42.7, 16.1. ESI-HRMS: m/z calcd for $C_{22}H_{16}BrNaNO_2S$ $[M + Na]^+$: 459.9983; found: 459.9978.

S-5 (1.67 g, 3.82 mmol) was combined with **S-4** (1.52 g, 3.82 mmol), Et_3N (0.25 mL, 1.91 mmol), $PdCl_2(PPh_3)_4$ (0.03 g, 0.038 mmol), Na_2CO_3 (0.20 g, 1.91 mmol), and 10 mL each of degassed toluene and water. The reaction mixture was refluxed for 4 h, then quenched with 50 mL of water, and extracted with ethyl acetate (3 × 50 mL). The organic layers were dried over Na_2SO_4, evaporated, and then purified by silica gel column chromatography (ethyl acetate/petroleum ether, 1:20) to give the product **S-6** (1.57 g, 65%) as a green solid. ^1H NMR ($CDCl_3$): δ = 8.02 (d, J = 6.8 Hz, 2H), 7.63-7.57 (m, 4H), 7.54 (d, J = 6.8 Hz, 2H), 7.48 (s, 1H), 7.40-7.29 (m, 7H), 4.86 (s, 2H), 2.04 (s, 6H), 1.65 (s, 9H). $^{13}C\{^1H\}$ NMR ($CDCl_3$): δ = 170.4, 170.3, 165.4, 142.6,

141.5, 141.4, 140.3, 137.4, 136.4, 133.6, 133.1, 132.6, 130.9, 129.0, 128.8, 128.1, 128.0, 127.8, 127.7, 125.6, 125.1, 124.2, 81.1, 42.2, 28.2, 15.2, 15.2. ESI-HRMS: m/z calcd for $C_{38}H_{33}NaNO_4S_2$ [M + Na]$^+$: 654.1749; found: 654.1742.

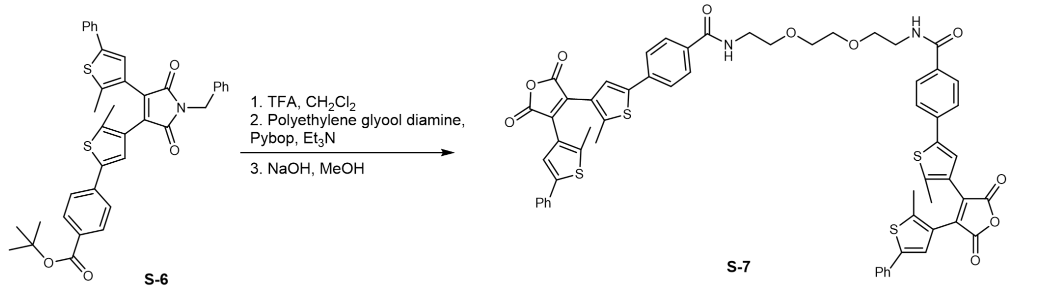

Supplementary Figure 6. Synthesis of **S-7**.

S-6 (1.56 g, 2.48 mmol) was dissolved in a mixture solution of CH_2Cl_2 (5 mL) and TFA (1 mL), and after stirring for 1 h at room temperature the reaction was quenched with water and extracted with CH_2Cl_2 (3 × 20 mL). The combined organic layers were washed with brine (3 × 20 mL), dried over Na_2SO_4, and evaporated to give a crude residue, which was used without further purification in the next step. The obtained crude product and PyBOP (1.29 g, 2.48 mmol) was dissolved in acetonitrile (10 mL). After stirring for 10 min, Et_3N (0.32 mL, 2.48 mmol) was added, and the reaction was continued to stirred for 30 min. The solution of 2,2′-(Ethylenedioxy)bis(ethylamine) (0.19 g, 1.25 mmol) in $CHCl_3$ (3 mL) was added dropwise. After TLC indicated that the reaction was complete, the mixture was filtered, and the solvents were evaporated *in vacuo*. The obtained crude product was then dissolved in MeOH (20 mL) under heating. NaOH aqueous solution (5 M, 2 mL) was added dropwise over 10 min, and the solution was stirred for 1 h at room temperature. The reaction mixture was then acidified with 2 M HCl (12 mL) and extracted with CH_2Cl_2. The combined organic layers were dried over Na_2SO_4, evaporated, and then purified by silica gel column chromatography (ethyl acetate/petroleum ether, 1:1) to give the product **S-7** (1.21 g, 45%) as an orange solid. ^{1}H NMR (CD_3CN): δ = 7.77 (d, J = 7.2 Hz, 4H), 7.59 (d, J = 6.8 Hz, 8H), 7.43-7.31 (m, 10H), 7.22 (t, J = 6.8 Hz, 2H), 3.65-3.62 (m, 8H), 3.54-3.50 (m, 4H), 2.00-1.95 (m, 12H). ^{13}C{^{1}H} NMR (CD_3CN:DMSO-d_6 = 1:1): δ = 165.6, 164.7, 164.7, 142.9, 142.0, 140.8, 139.8, 135.1, 134.7, 133.4, 132.6, 128.9, 127.9, 127.7, 126.8, 124.9, 124.7, 124.6, 123.6, 69.5, 68.7, 39.1, 14.0, 13.9. ESI-HRMS: m/z calcd for $C_{60}H_{48}NaN_2O_{10}S_4$ [M + Na]$^+$: 1107.1880; found: 1107.1884.

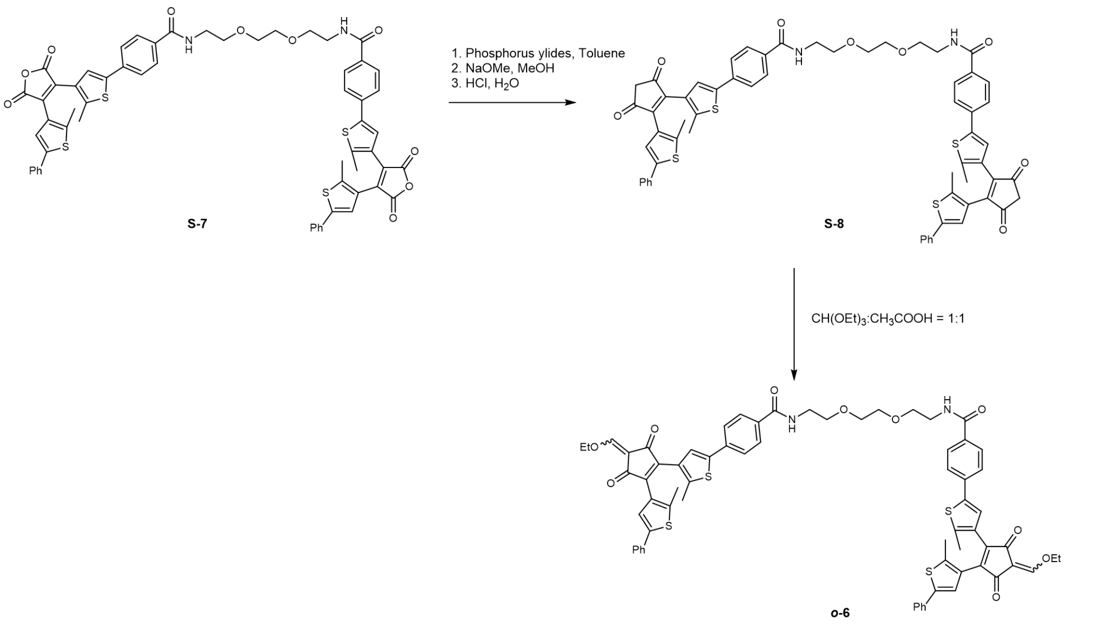

Supplementary Figure 7. Synthesis of ***o*-6**.

The compound was synthesized from **S-7** (1.25 g, 1.13 mmol) according to the procedure of **S-2** and obtained **S-8** as a green solid (0.61 g, 50%). ^{1}H NMR ($CDCl_3$): δ = 7.70 (d, *J* = 7.2 Hz, 4H), 7.48-7.43 (m, 8H), 7.28 (t, *J* = 7.2 Hz, 4H), 7.23-7.18 (m, 4H), 7.13 (s, 2H), 6.67 (t, *J* = 6.8 Hz, 2H), 3.60-3.57 (m, 12H), 3.15 (s, 4H), 1.94 (s, 6H), 1.93 (s, 6H). ^{13}C{^{1}H} NMR ($CDCl_3$): δ = 198.7, 198.7, 166.9, 150.5, 149.9, 142.5, 141.7, 141.4, 140.2, 136.5, 133.5, 133.1, 129.4, 128.3, 128.0, 127.8, 125.6, 125.4, 125.2 70.2, 68.6, 42.1, 39.8, 15.1. ESI-HRMS: m/z calcd for $C_{62}H_{52}N_2NaO_8S_4$ $[M + Na]^+$: 1103.2504; found: 1103.2507.

The compound was synthesized from **S-8** (0.61 g, 0.57 mmol) according to the procedure of ***o*-1** and obtained ***o*-6** as a yellow solid and a mixture of *E*/*Z* isomers (0.36 g, 50%). ^{1}H NMR (CD_3CN): δ = 7.75-7.74 (d, *J* = 6.8 Hz, 4H), 7.59-7.52 (m, 10H), 7.36 (t, *J* = 7.2 Hz, 4H), 7.30-7.22 (m, 6H), 7.16-7.14 (m, 2H), 4.44 (q, *J* = 7.2 Hz, 4H), 3.65-3.63 (m, 8H), 3.56-3.51 (m, 4H), 2.07-2.01 (m, 12H), 1.43 (t, *J* = 7.2 Hz, 6H). ^{13}C{^{1}H} NMR (CD_3CN:$CDCl_3$ = 5:1): 205.8, 200.5, 198.8, 192.2, 190.3, 188.9, 166.7, 160.4, 146.3, 146.0, 144.8, 144.5, 141.8, 141.6, 140.7, 140.6, 139.5, 136.4, 136.3, 134.2, 133.5, 133.1, 129.2, 129.0, 127.9, 125.9, 125.8, 125.3, 125.1, 124.7, 124.6, 73.9, 70.1, 68.7, 38.2, 15.1, 14.6. ESI-HRMS: m/z calcd for $C_{68}H_{60}N_2NaO_{10}S_4$ $[M + Na]^+$: 1215.3028; found: 1215.3024.

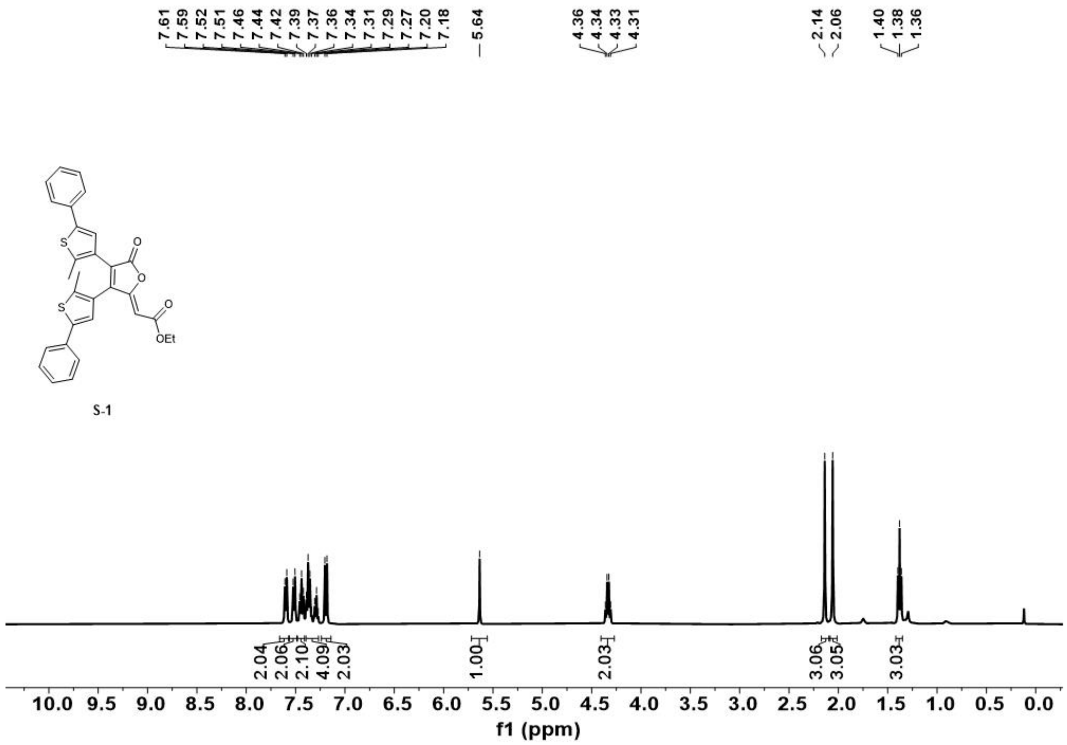

Supplementary Figure 8. ^{1}H NMR (400 MHz, 20 °C) spectrum of **S-1** in $CDCl_3$.

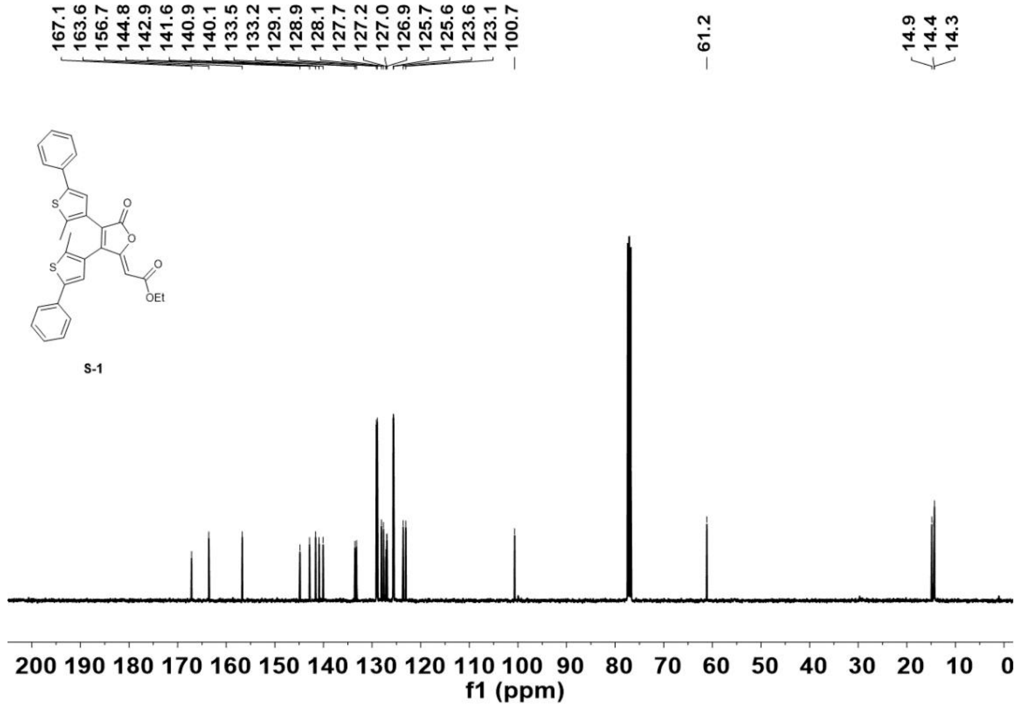

Supplementary Figure 9. ^{13}C NMR (100 MHz, 20 °C) spectrum of **S-1** in $CDCl_3$.

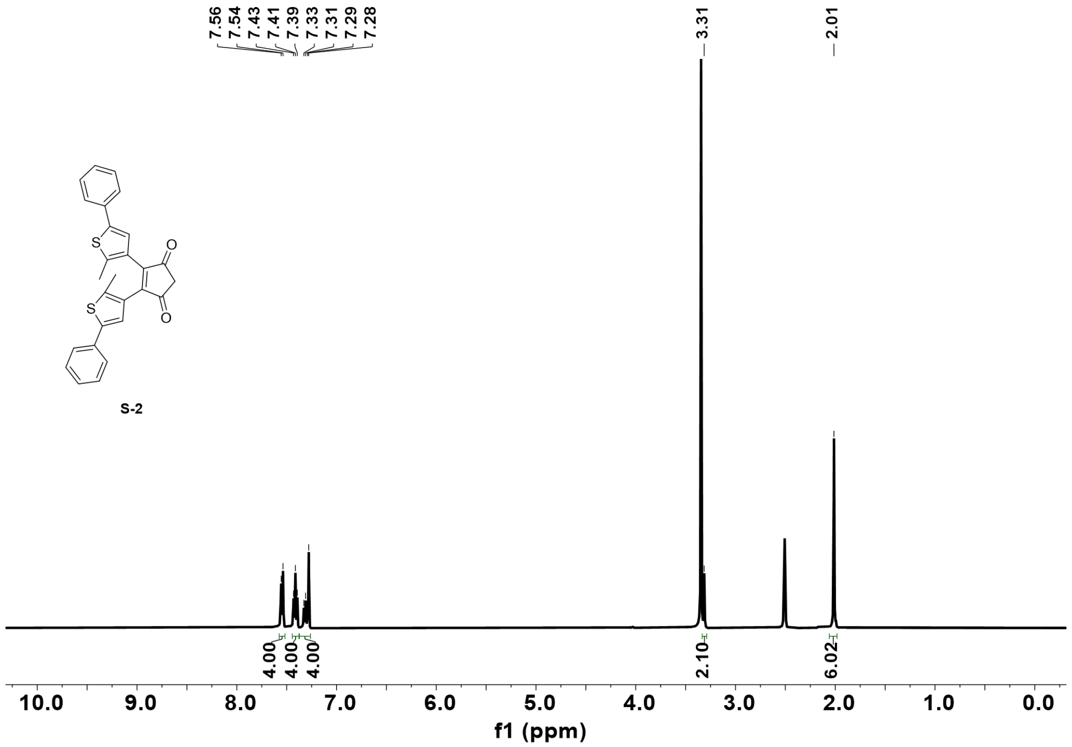

Supplementary Figure 10. ^{1}H NMR (400 MHz, 20 °C) spectrum of **S-2** in DMSO-d_6.

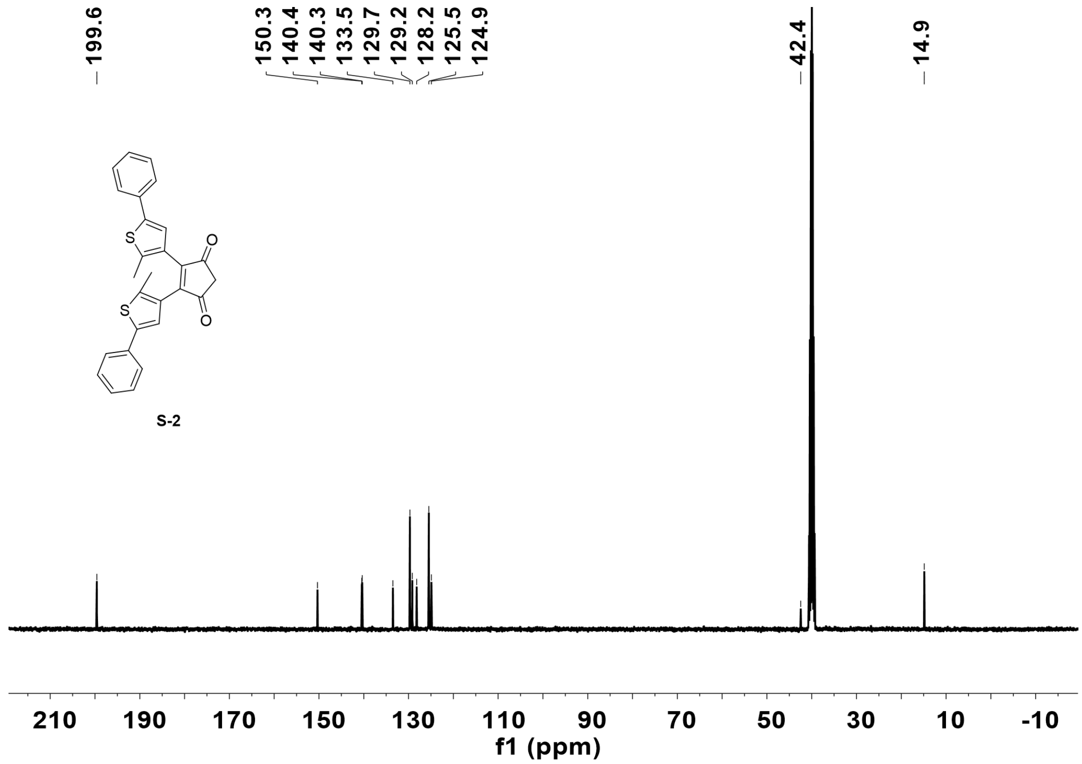

Supplementary Figure 11. ^{13}C NMR (100 MHz, 20 °C) spectrum of **S-2** in DMSO-d_6.

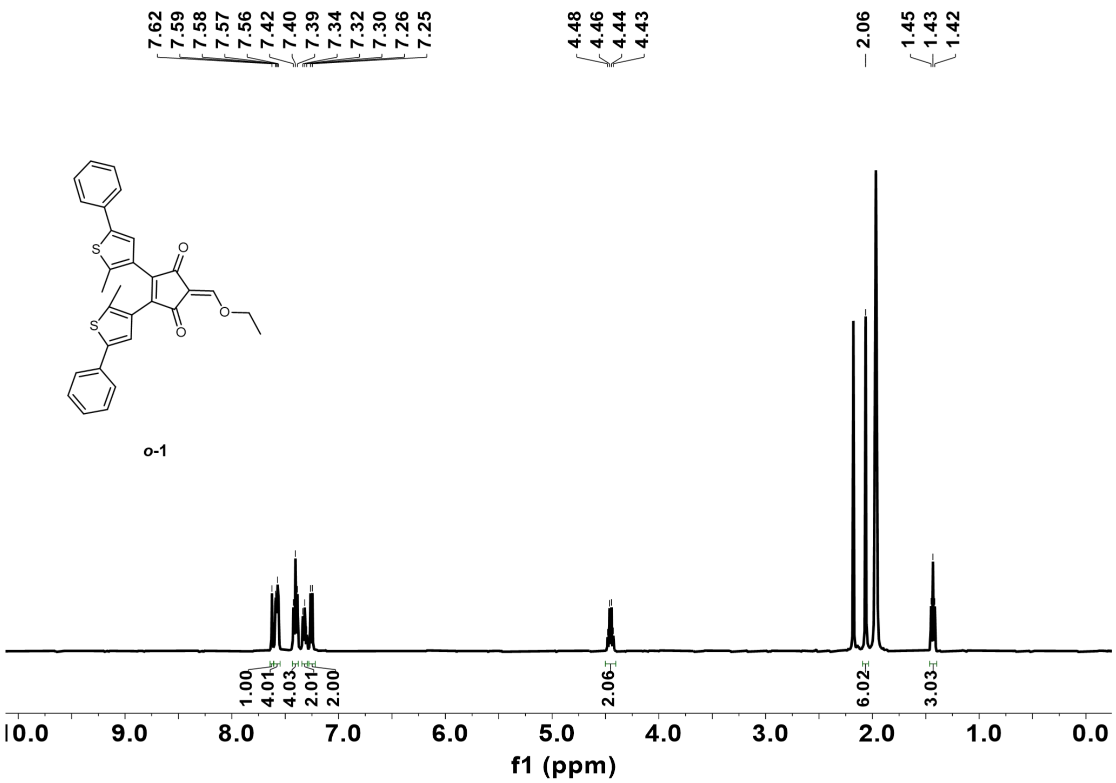

Supplementary Figure 12. ^{1}H NMR (400 MHz, 20 °C) spectrum of ***o*-1** in CD_3CN.

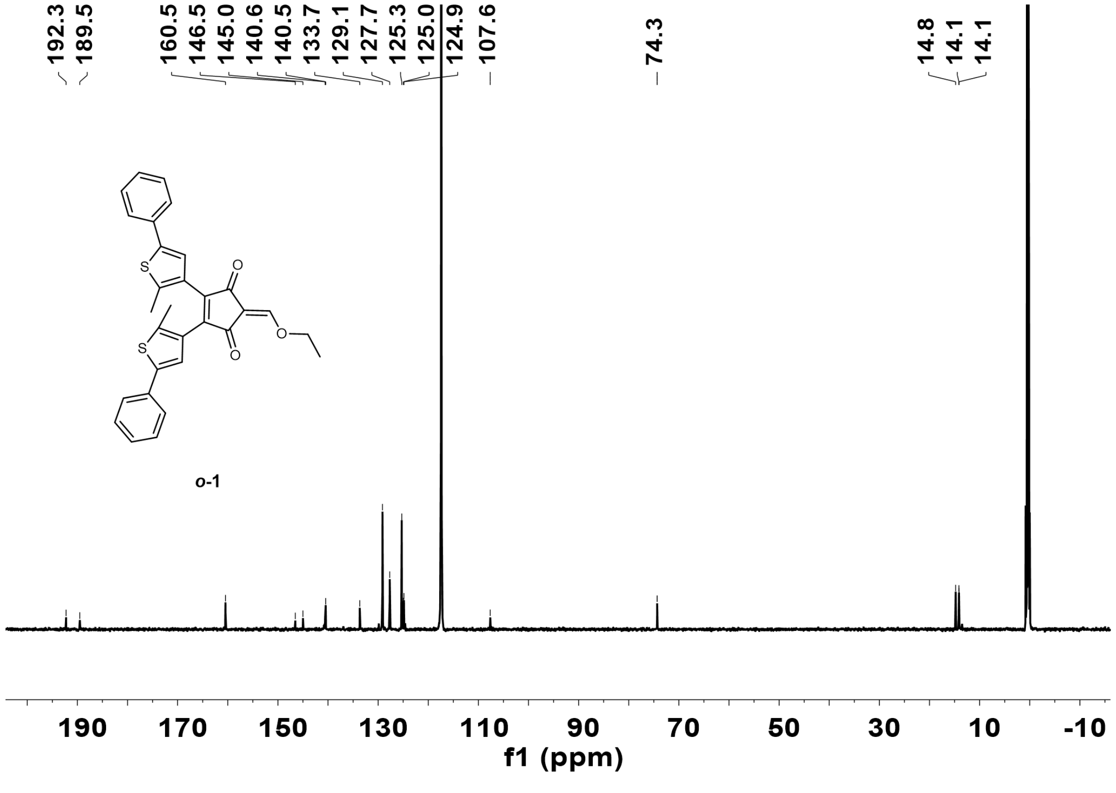

Supplementary Figure 13. ^{13}C NMR (100 MHz, 20 °C) spectrum of ***o*-1** in CD_3CN.

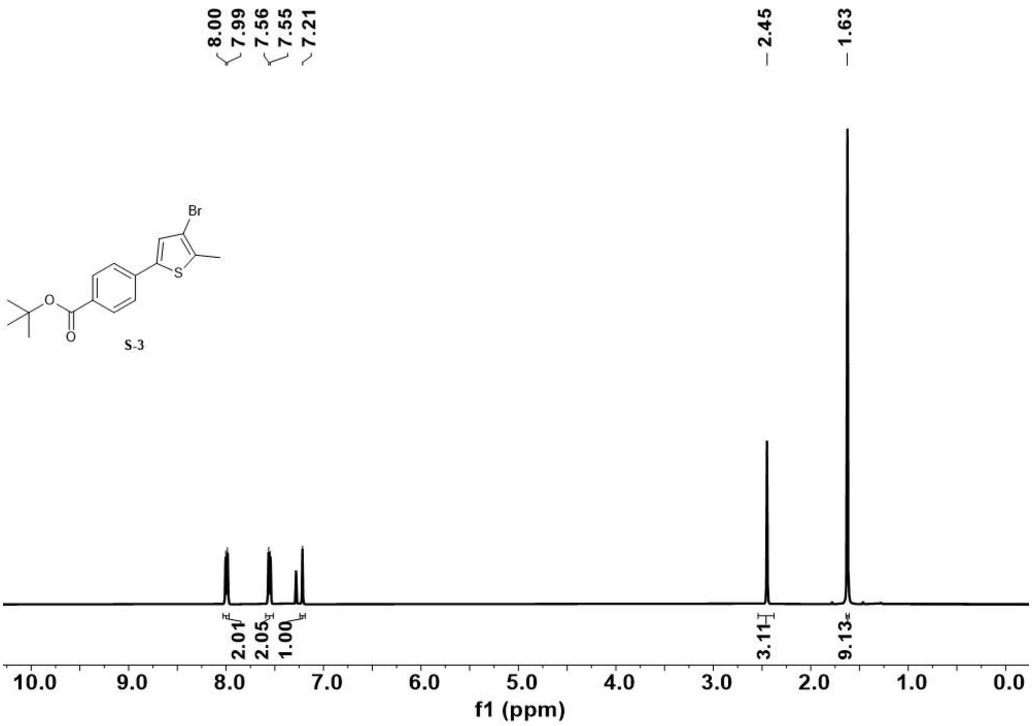

Supplementary Figure 14. ^{1}H NMR (400 MHz, 20 °C) spectrum of **S-3** in $CDCl_3$.

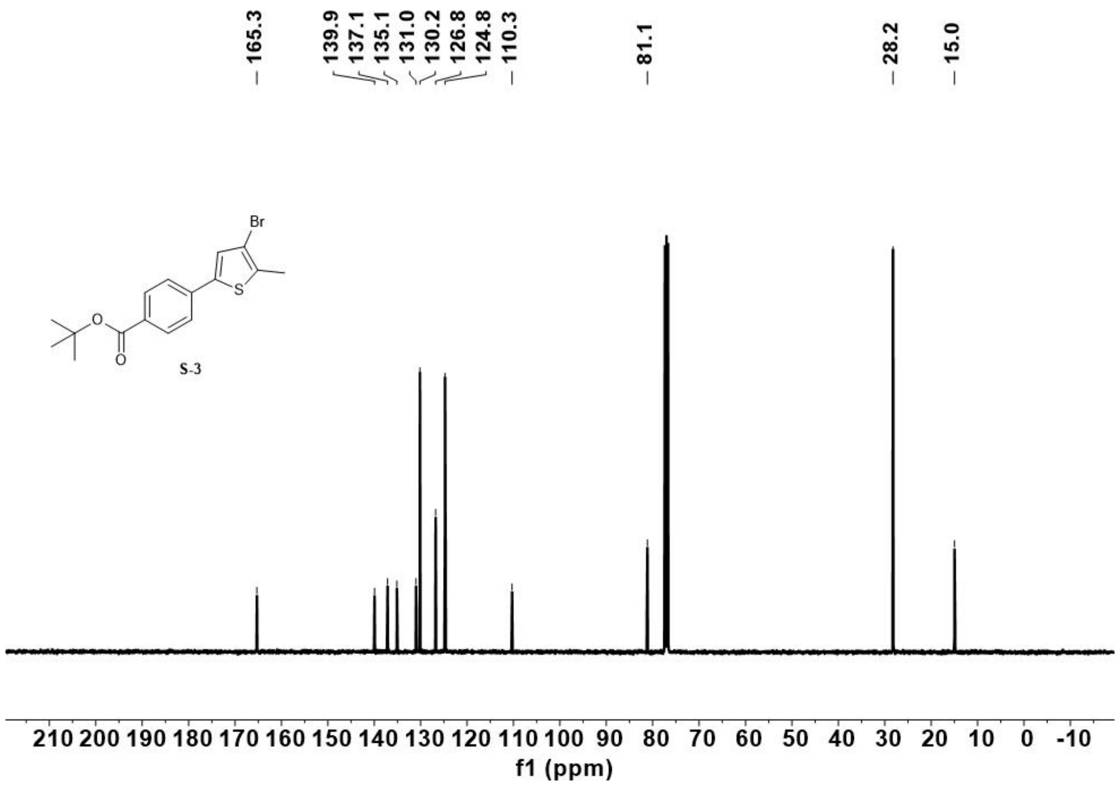

Supplementary Figure 15. ^{13}C NMR (100 MHz, 20 °C) spectrum of **S-3** in $CDCl_3$.

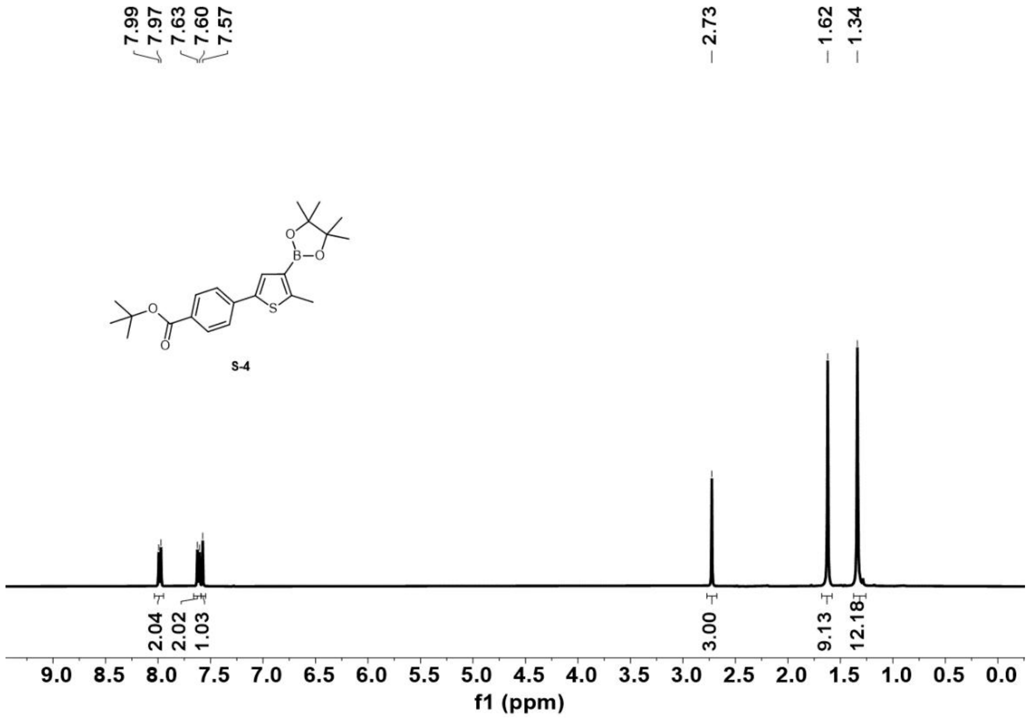

Supplementary Figure 16. ^{1}H NMR (400 MHz, 20 °C) spectrum of **S-4** in $CDCl_3$.

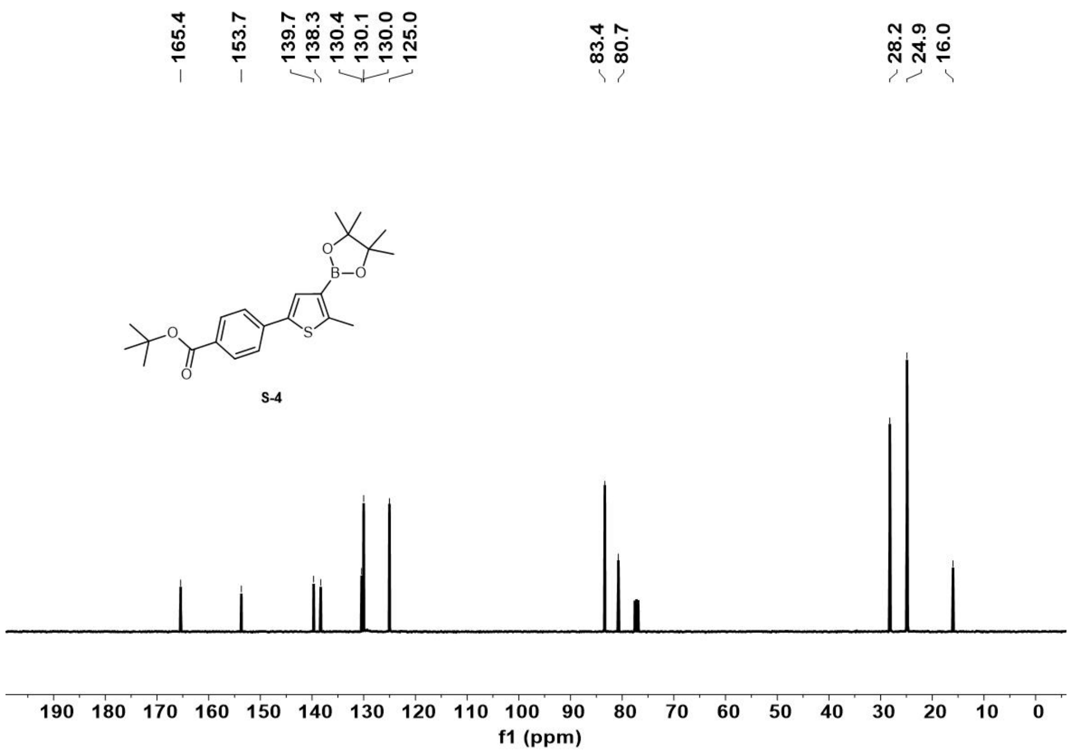

Supplementary Figure 17. ^{13}C NMR (100 MHz, 20 °C) spectrum of **S-4** in $CDCl_3$.

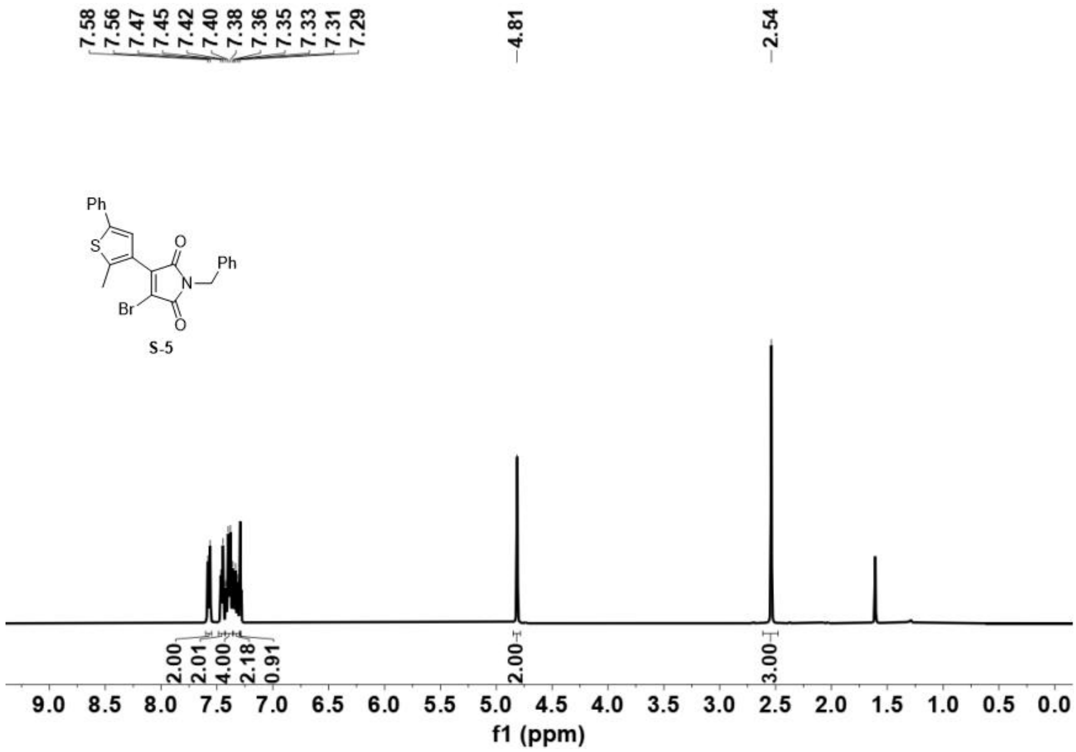

Supplementary Figure 18. ^{1}H NMR (400 MHz, 20 °C) spectrum of **S-5** in $CDCl_3$.

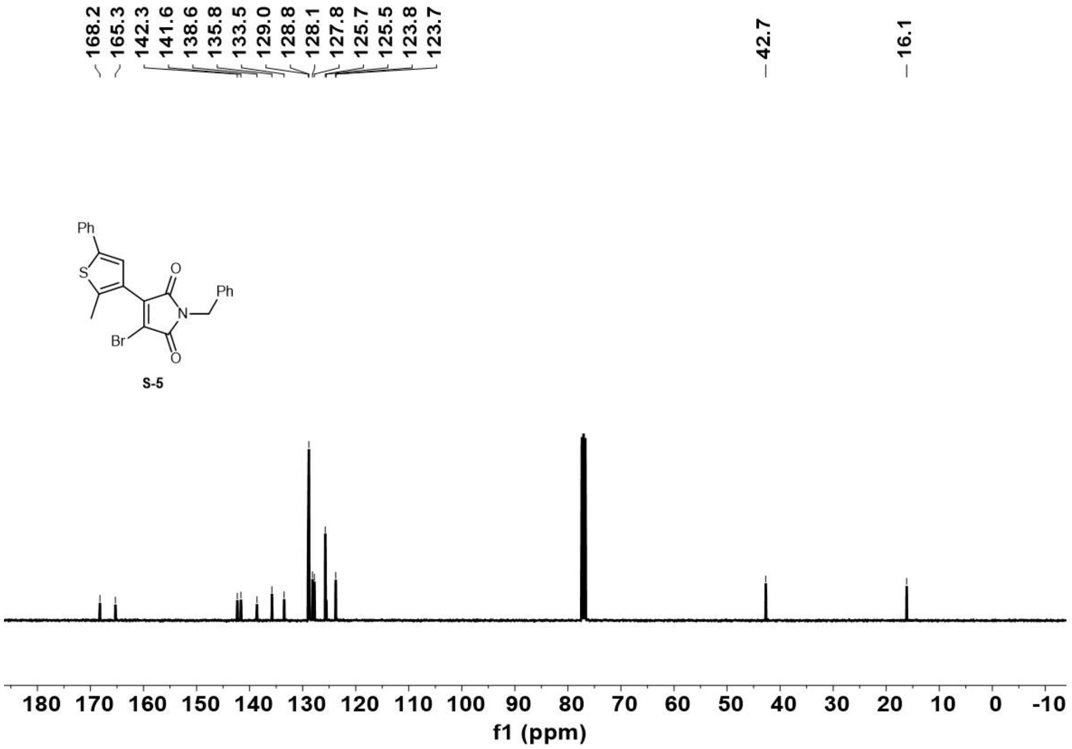

Supplementary Figure 19. ^{13}C NMR (100 MHz, 20 °C) spectrum of **S-5** in $CDCl_3$.

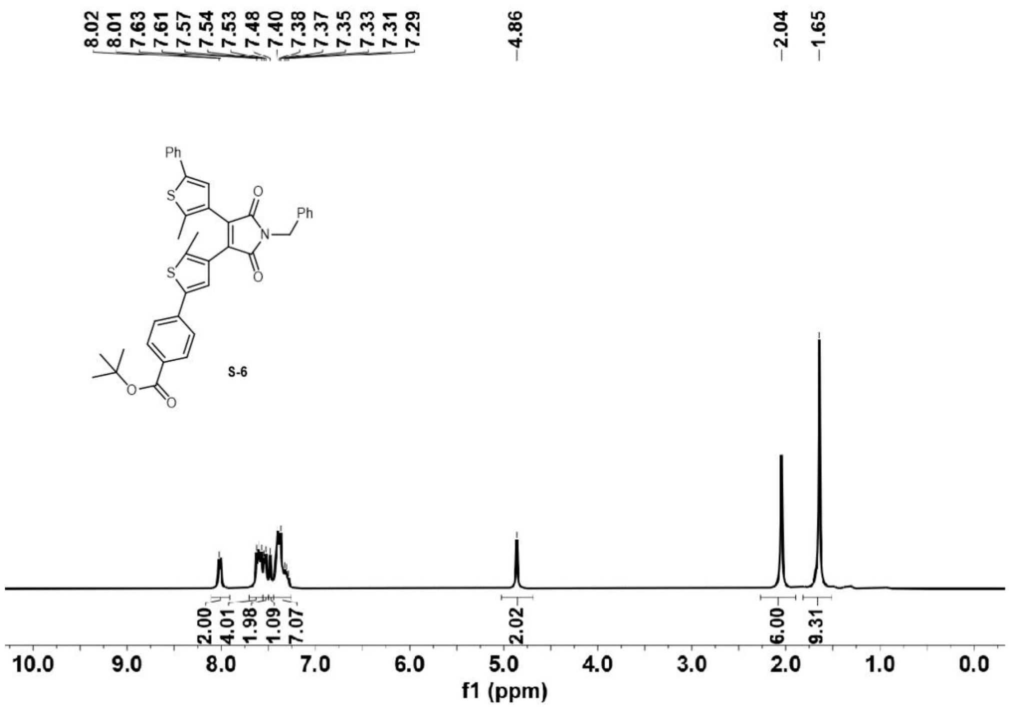

Supplementary Figure 20. ^{1}H NMR (400 MHz, 20 °C) spectrum of **S-6** in $CDCl_3$.

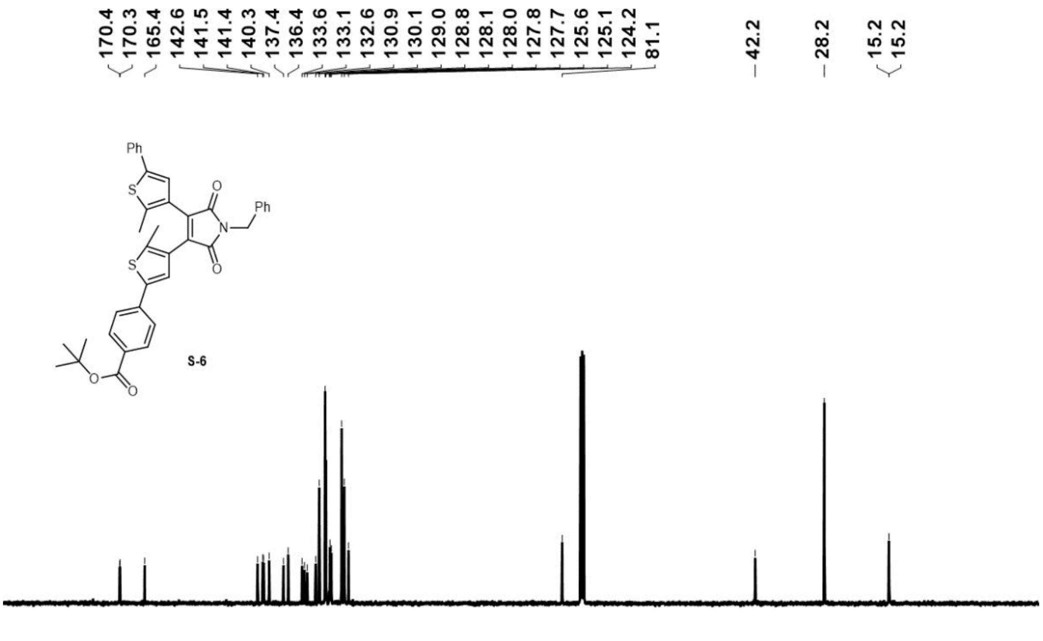

Supplementary Figure 21. ^{13}C NMR (100 MHz, 20 °C) spectrum of **S-6** in $CDCl_3$.

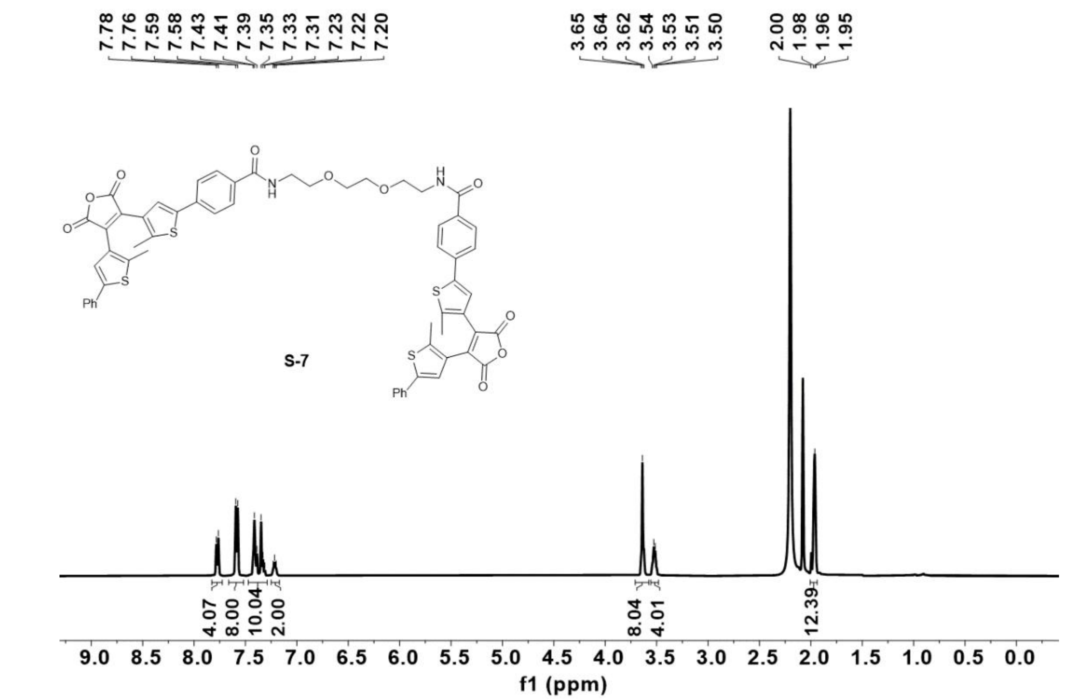

Supplementary Figure 22. ^{1}H NMR (400 MHz, 20 °C) spectrum of **S-7** in CD_3CN.

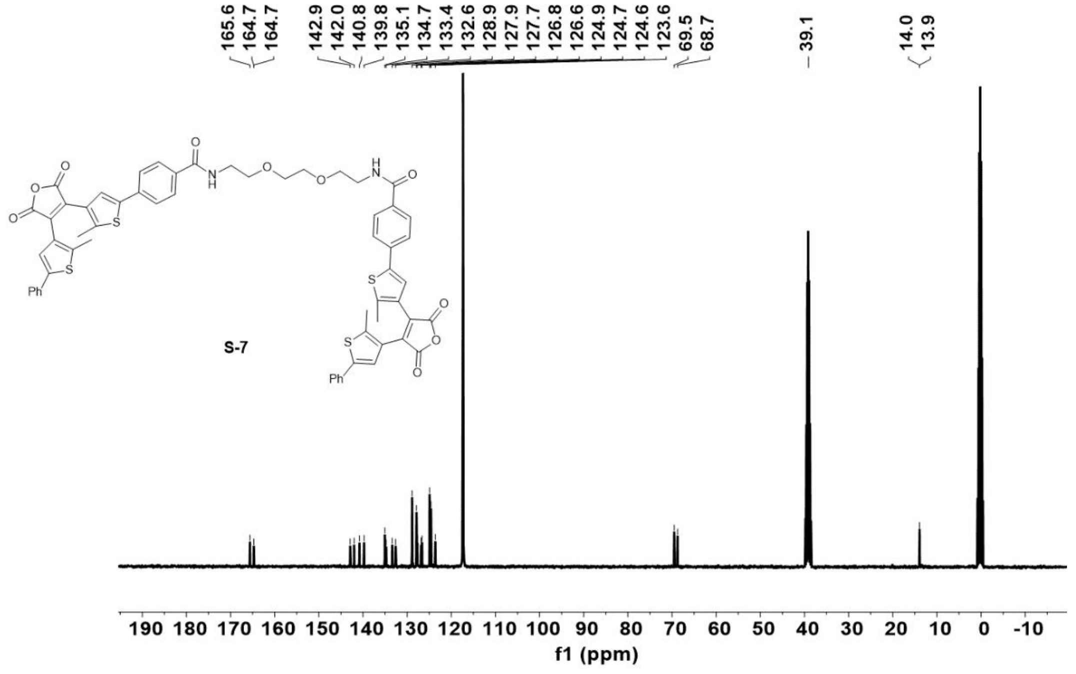

Supplementary Figure 23. ^{13}C NMR (100 MHz, 20 °C) spectrum of **S-7** in CD_3CN:DMSO-d_6 (1:1).

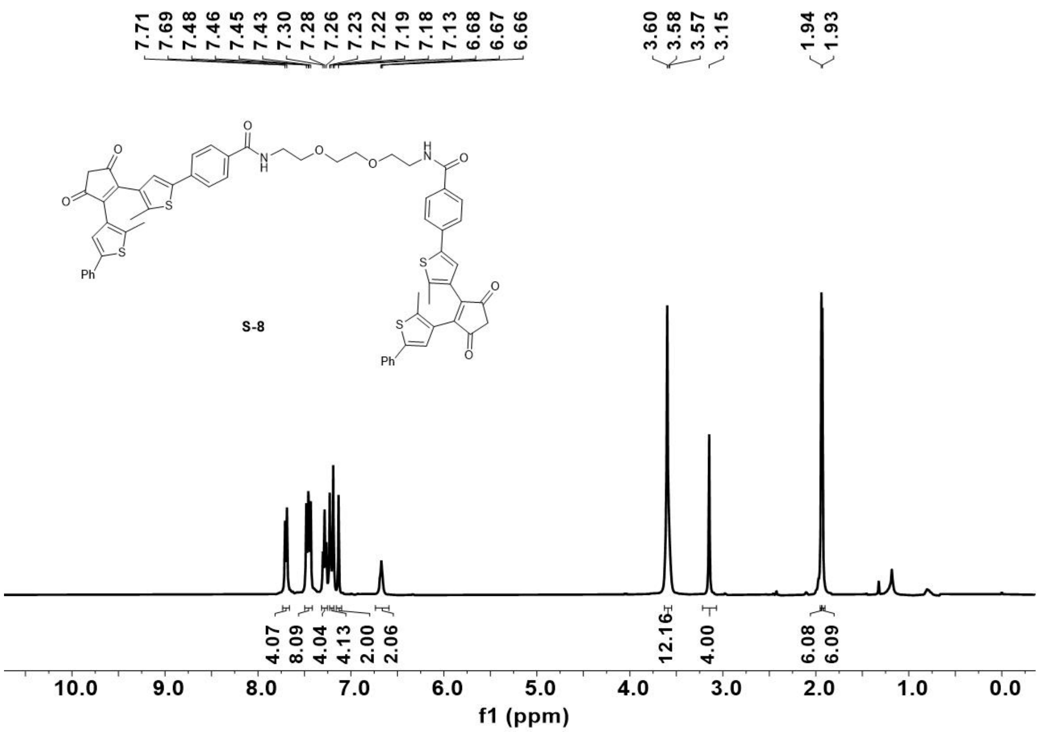

Supplementary Figure 24. ^{1}H NMR (400 MHz, 20 °C) spectrum of **S-8** in $CDCl_3$.

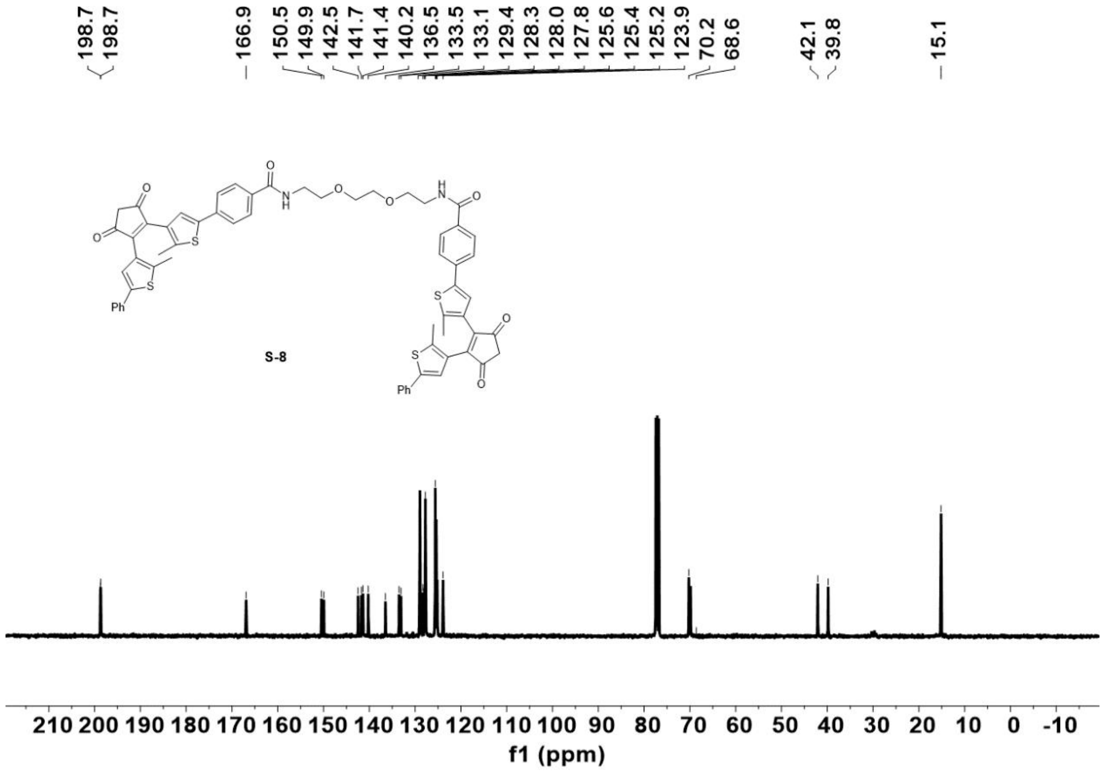

Supplementary Figure 25. ^{13}C NMR (100 MHz, 20 °C) spectrum of **S-8** in $CDCl_3$.

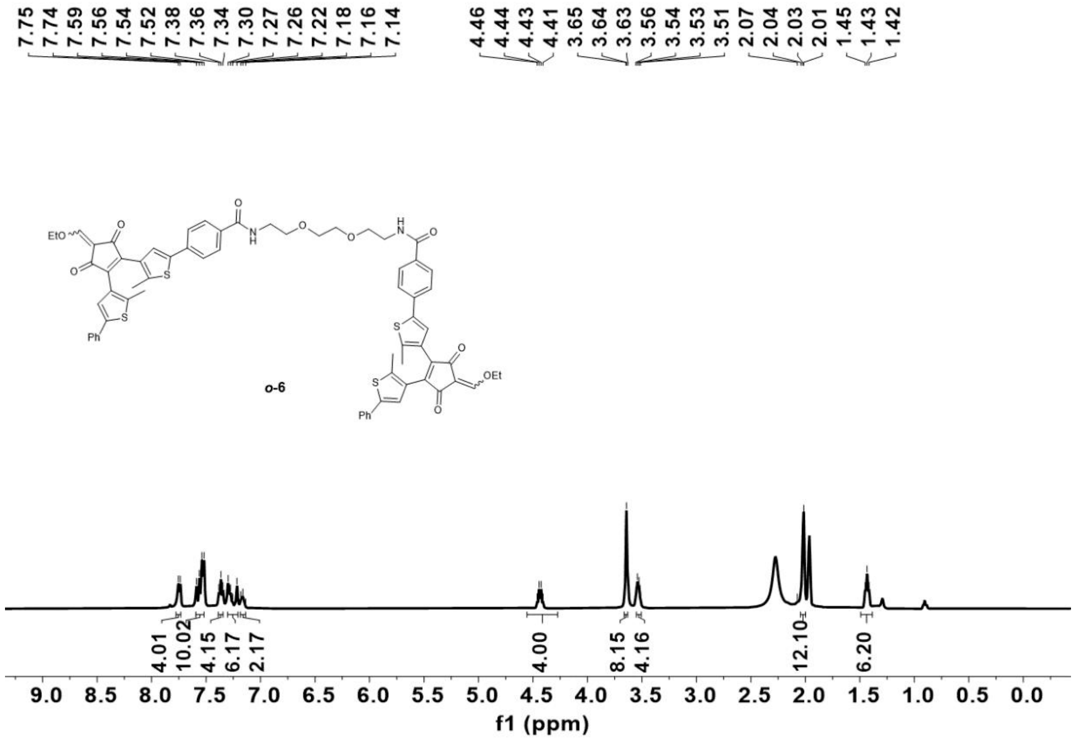

Supplementary Figure 26. ^{1}H NMR (400 MHz, 20 °C) spectrum of ***o*-6** in CD_3CN.

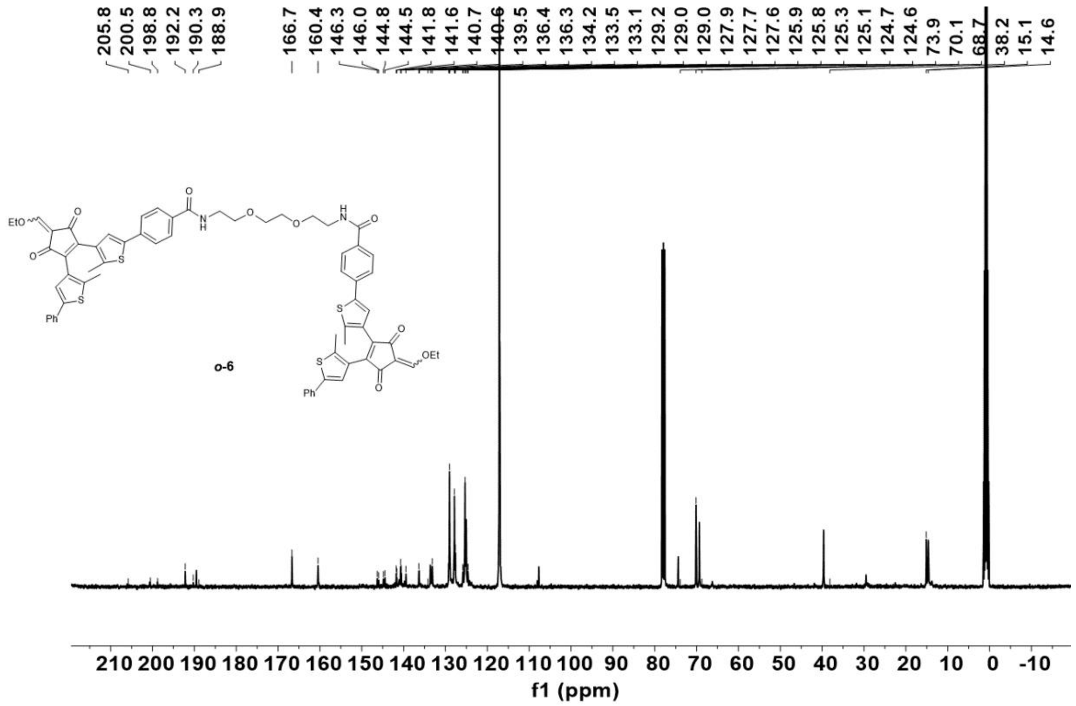

Supplementary Figure 27. ^{13}C NMR (100 MHz, 20 °C) spectrum of ***o*-6** in $CD_3CN:CDCl_3$ (5:1).

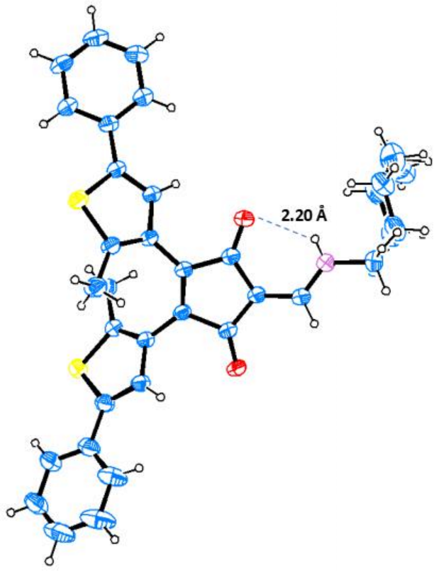

Supplementary Figure 28. Crystal structures of ***o*-3**(1-butylamine). The thermal ellipsoids were scaled to the 30% probability level for ***o*-3**(1-butylamine).

Supplementary Table 1. Summary of crystallographic data.

**Compound**	***o*-3**(1-butylamine)
Formula	$C_{32}H_{29}NO_2S_2$
Formula weight	523.68
T/*K*	293(2)
Crystallization	acetonitrile
Color	yellow
Crystal system	monoclinic
Space group	$P2_1/c$
a / Å	16.8382(8)
b / Å	9.0005(6)
c / Å	18.3050(11)
α / °	90
β / °	98.603(5)
γ / °	90
V/ $Å^3$	2742.9(3)
Z	4
D_x / g cm^{-3}	1.268
μ / mm^{-1}	1.287
F(000)	1104
θ range / º	2.307 to 60.697
GOF on F^2	1.040
R_1 [$I > 2σ(I)$]	0.0572
wR_2 (all data)	0.1474

2. Photoswitching Experiments

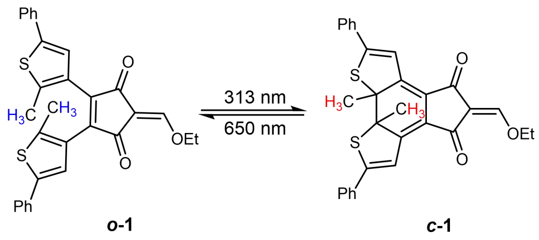

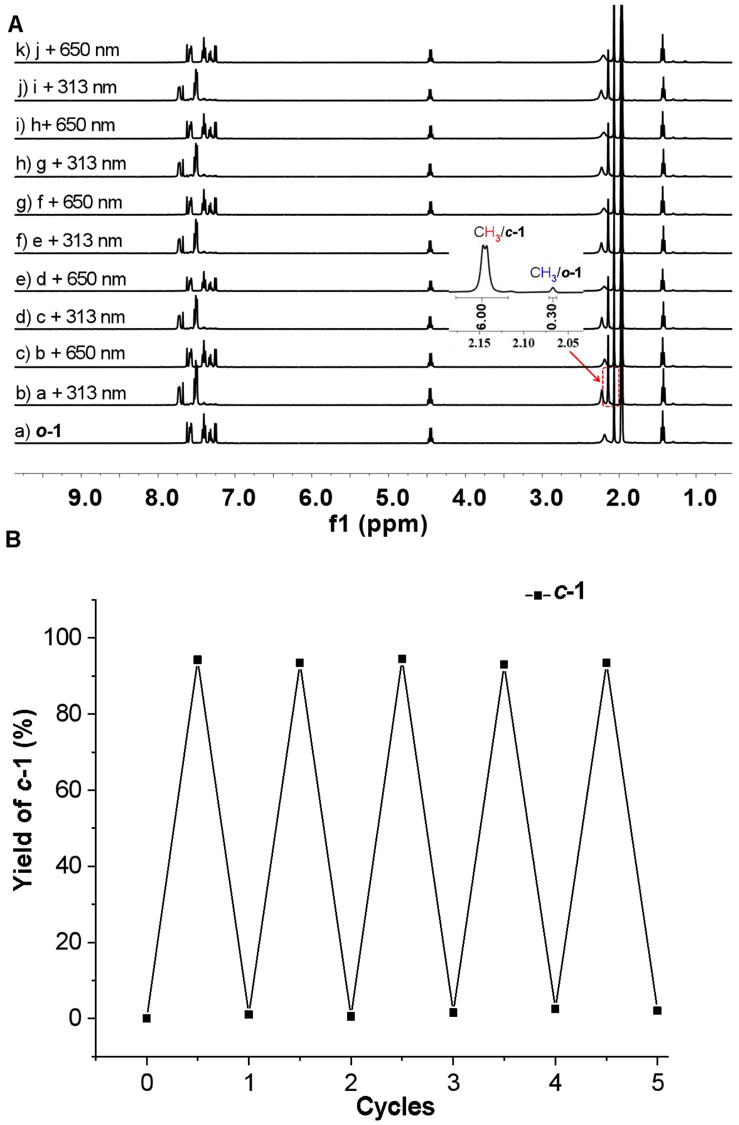

Supplementary Figure 29. (A) (a) ^{1}H NMR (400 MHz, 20 °C) spectrum of ***o*-1** (5 mM) in CD_3CN; (b) Irradiation of ***o*-1** with UV light (313 nm, 1.5 h). The ratio of ***c*-1** and ***o*-1** is 95:5; (c) Further irradiation with visible light (650 nm, 2 h). The ratio of ***o*-1** and ***c*-1** is 100:0; (d-k) Continued photoswitching for four cycles. (B) Variation of the yield of ***o*-1** in photoswitching cycle monitored by ^{1}H NMR (400 MHz, 20 °C).

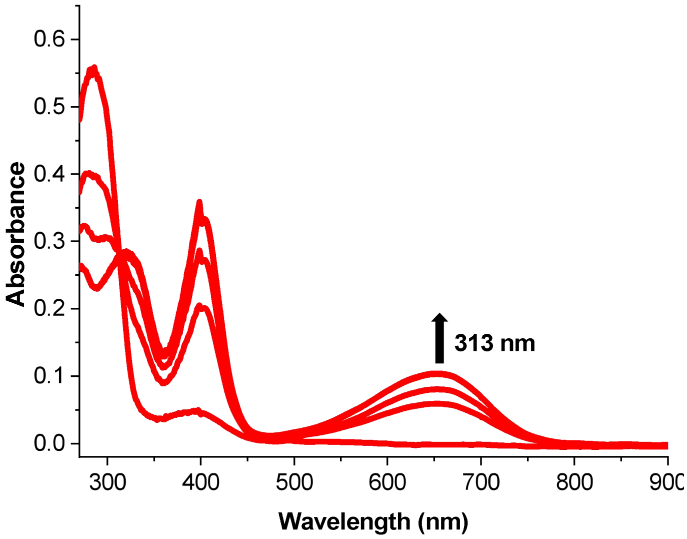

Supplementary Figure 30. Photocyclization of ***o*-1** to give ***c*-1**: changes in absorption spectra upon irradiation of ***o*-1** (25 µM in CH_3CN, 25 °C) with 313 nm light after total irradiation time of 0, 5, 10, 15, 20, and 25 s. The photostationary state was reached after 15 s.

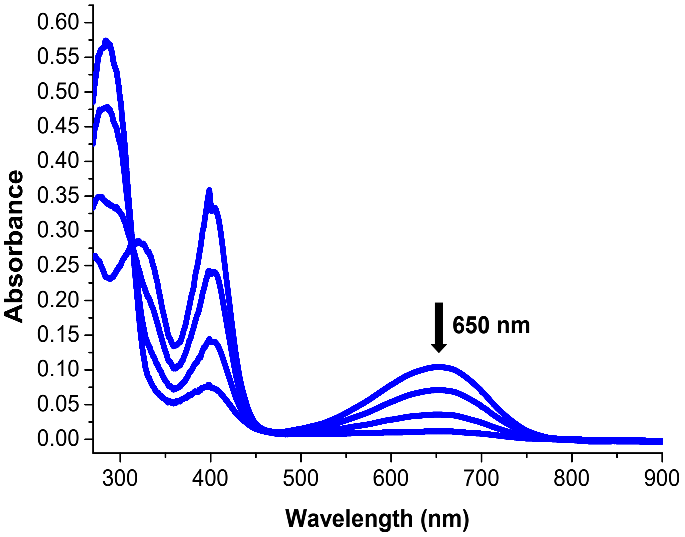

Supplementary Figure 31. Photocycloreversion of ***c*-1** to give ***o*-1**: changes in absorption spectra upon irradiation of ***c*-1** (25 µM in CH_3CN, 25 °C) with 650 nm light after total irradiation of 0, 4, 8, 12, and 15 min. The photostationary state was reached after 12 min.

3. Light-Controlled Exchange of Alcohol Nucleophiles

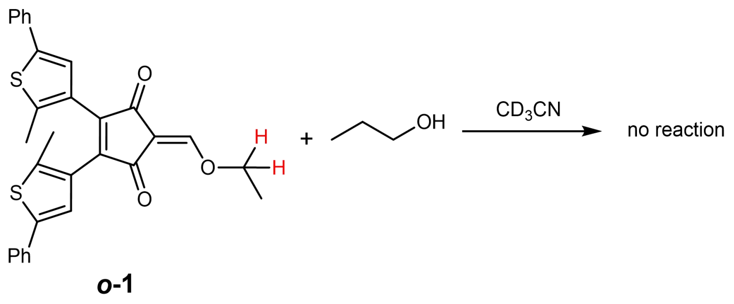

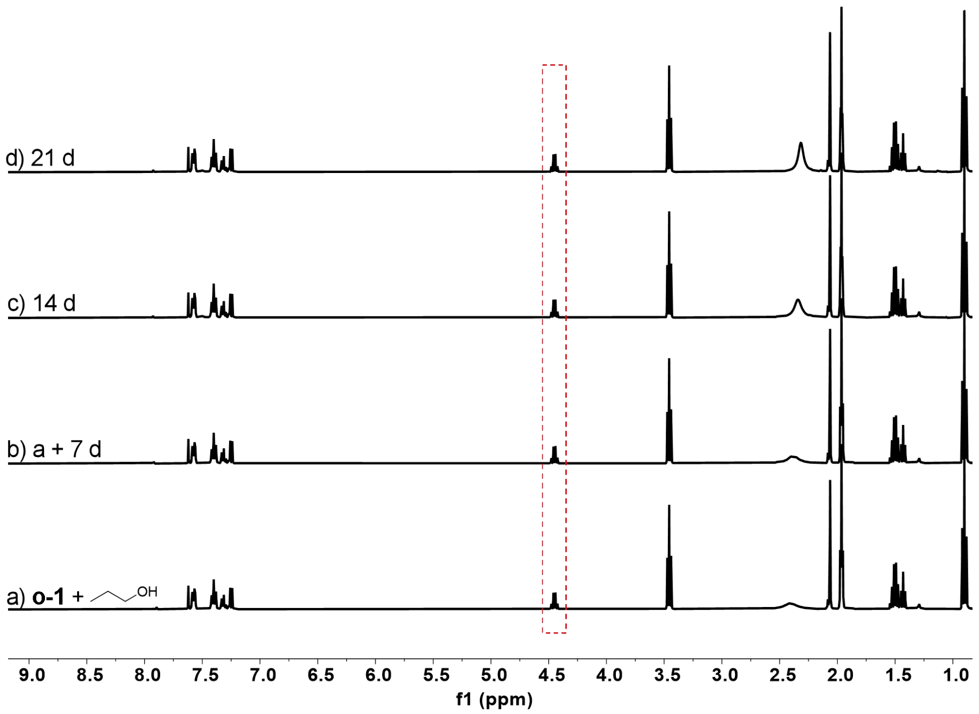

Supplementary Figure 32. Stacked ^{1}H NMR (400 MHz, 20 °C) spectra of the reaction of ***o*-1** (5 mM) with 1-propanol (3.0 equiv.) in CD_3CN at varied time. No reaction occurred.

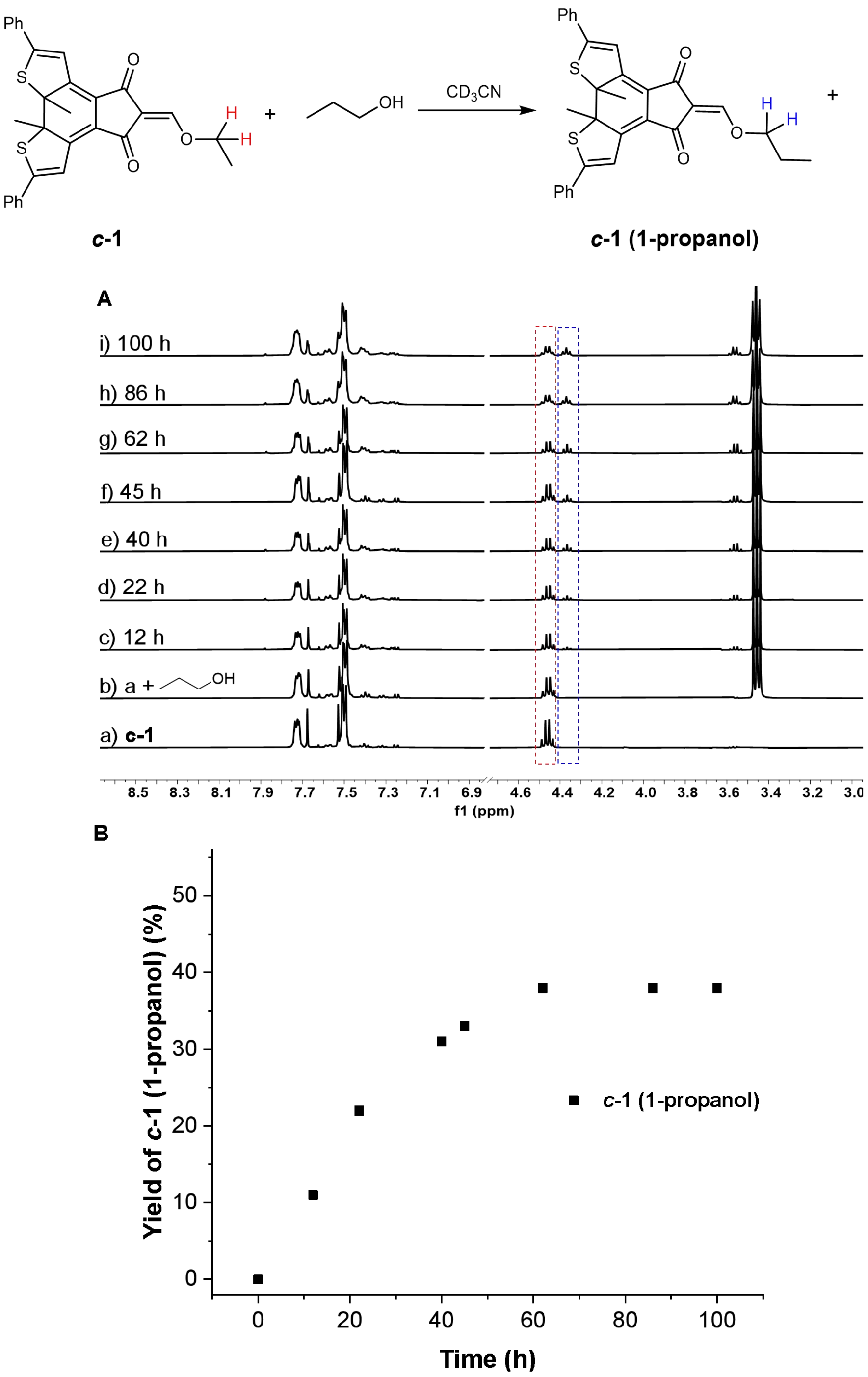

Supplementary Figure 33. (A) Stacked ^{1}H NMR (400 MHz, 20 °C) spectra of the reaction of ***o*-1** (5 mM) with 1-propanol (3.0 equiv.) in CD_3CN at varied time. (B) The kinetics profile of the reaction of ***o*-1** with aniline in 100 h.

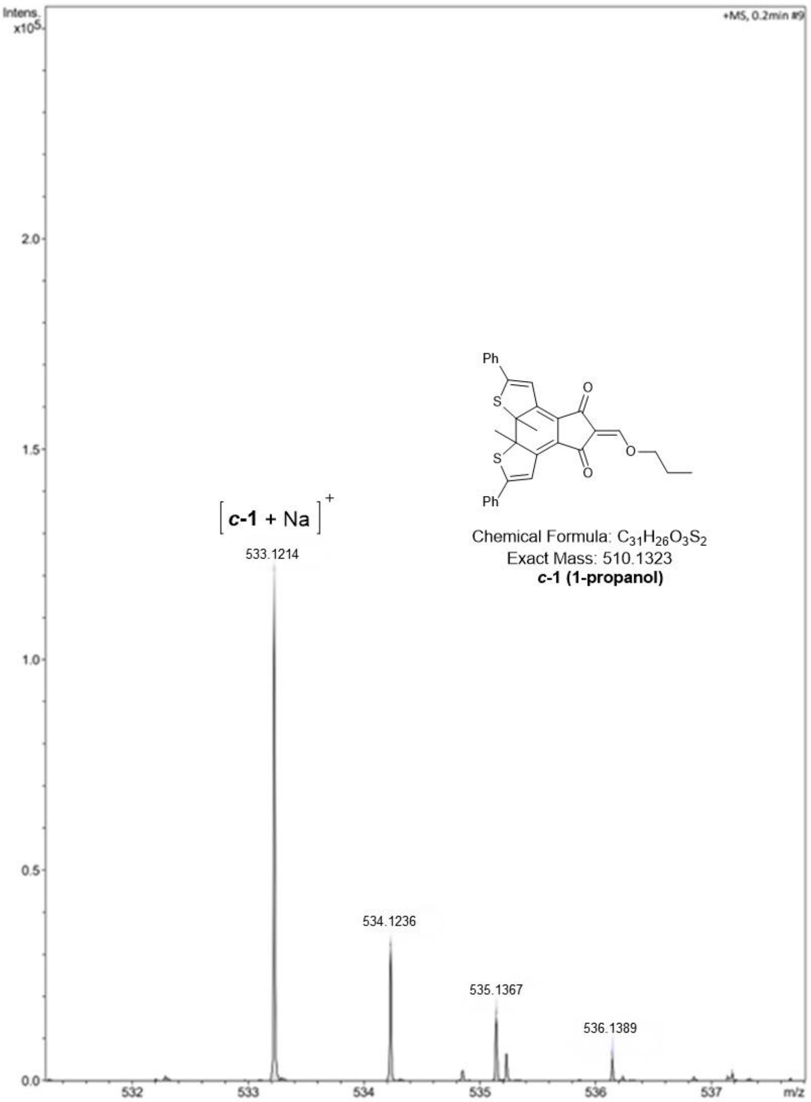

Supplementary Figure 34. ESI mass spectrum of the reaction of ***c*-1** and 1-propanol in acetonitrile.

4. Light-Controlled Exchange of Thiol Nucleophiles

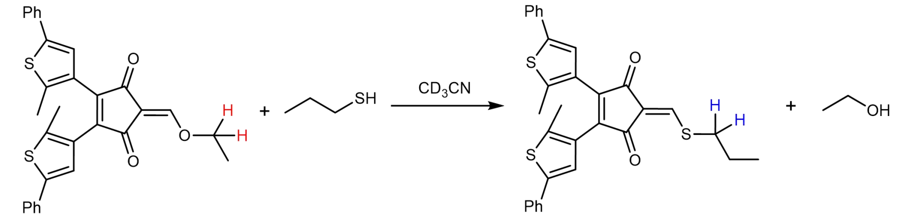

o-1 *o*-2 (1-propanethiol)

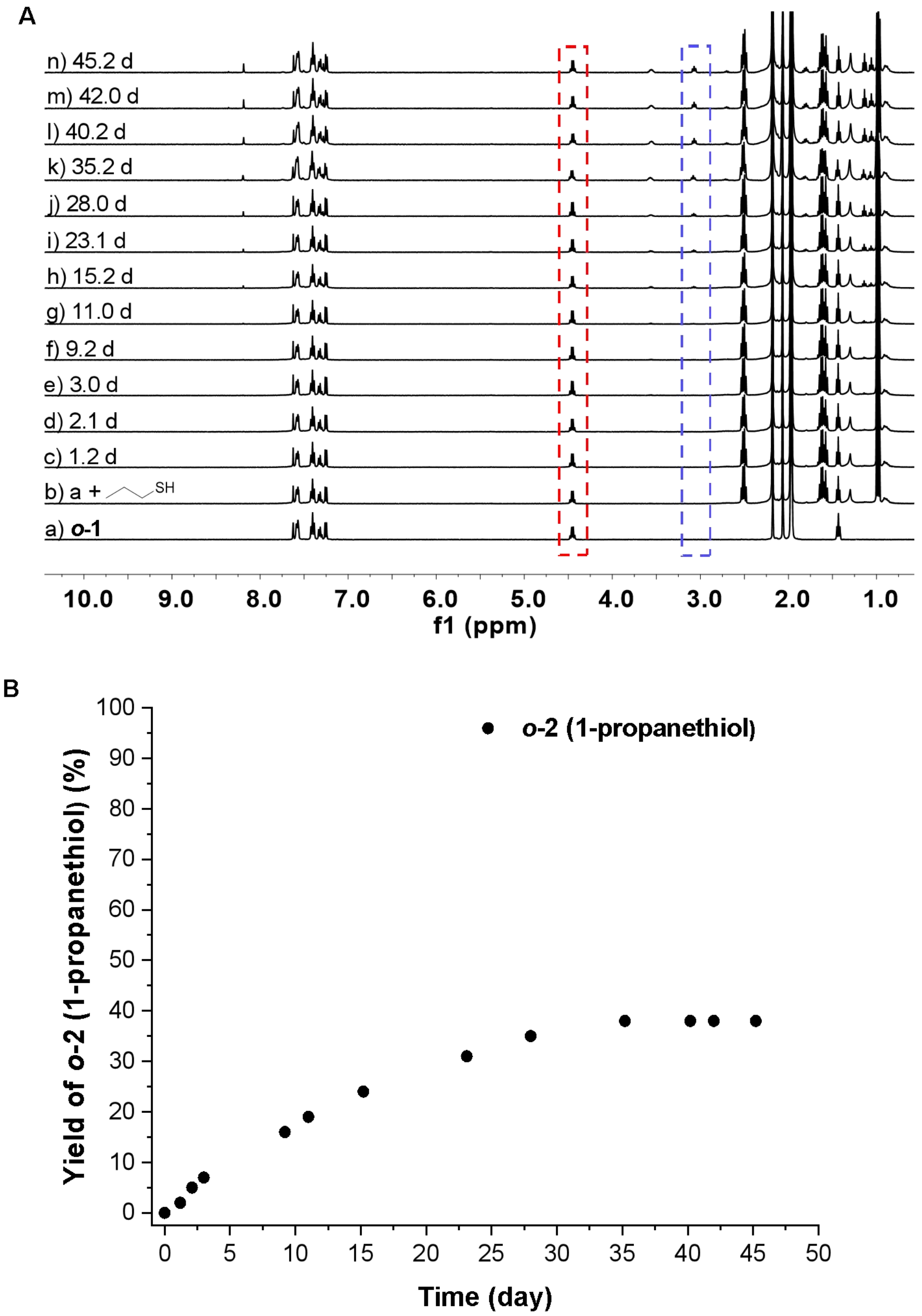

Supplementary Figure 35. (A) Stacked ^{1}H NMR (400 MHz, 20 °C) spectra of the reaction of ***o*-1** (5 mM) with 1-propanethiol (3 equiv.) in CD_3CN at varied time. (B) The kinetics profile of the reaction of ***o*-1** with 1-propanethiol in 45 d.

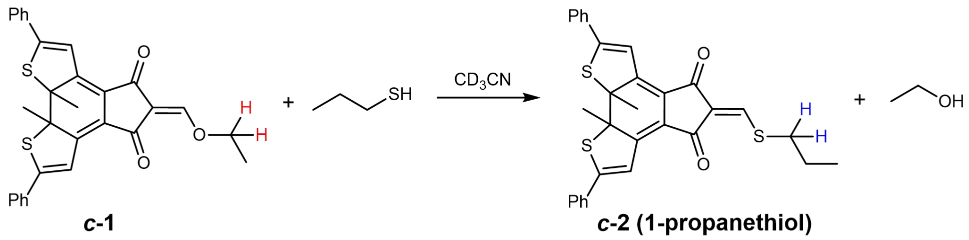

A

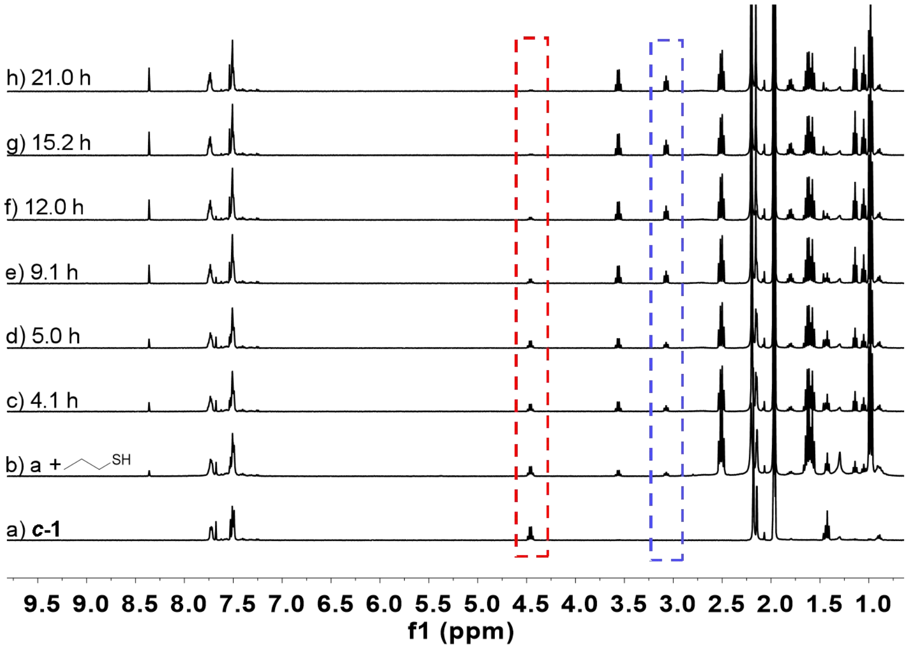

B

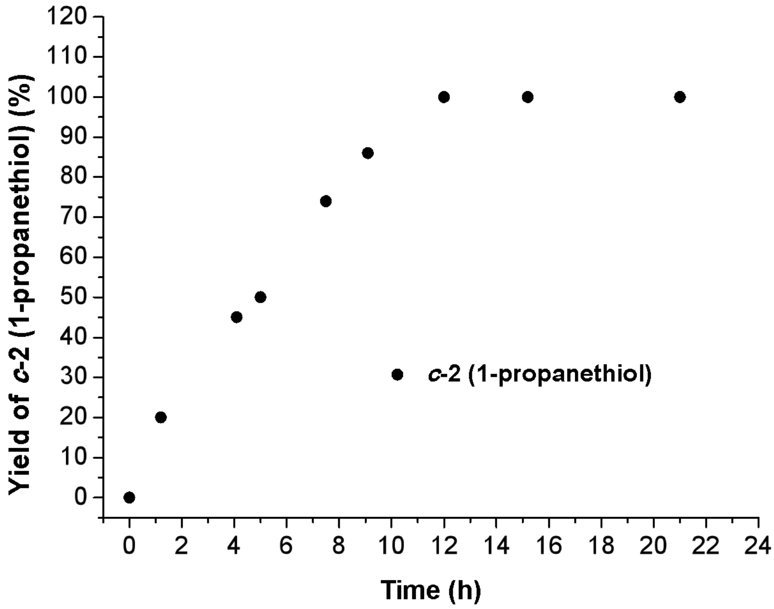

Supplementary Figure 36. (A) Stacked ^{1}H NMR (400 MHz, 20 °C) spectra of the reaction of ***c*-1** (5 mM) with 1-propanethiol (3 equiv.) in CD_3CN at varied time. (B) The kinetics profile of the reaction of ***c*-1** with 1-propanethiol in 22 h.

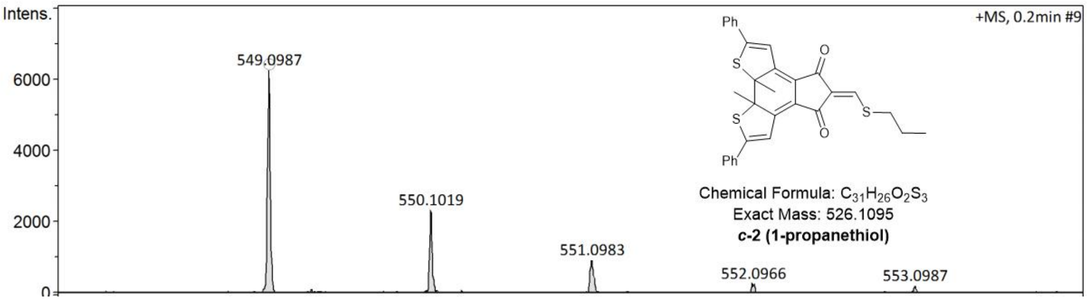

Supplementary Figure 37. ESI mass spectrum of the reaction of ***c*-1** and 1-propanethiol in acetonitrile.

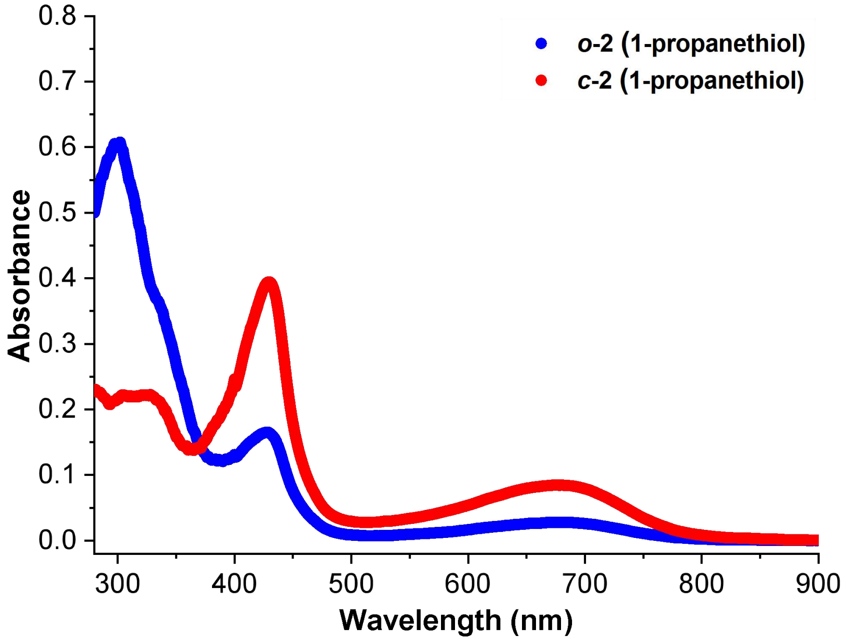

Supplementary Figure 38. Absorbance spectra of ***o*-2** (blue, 25 μM, 25 °C) and ***c*-2** (red, 25 μM, 25 °C) in CH_3CN.

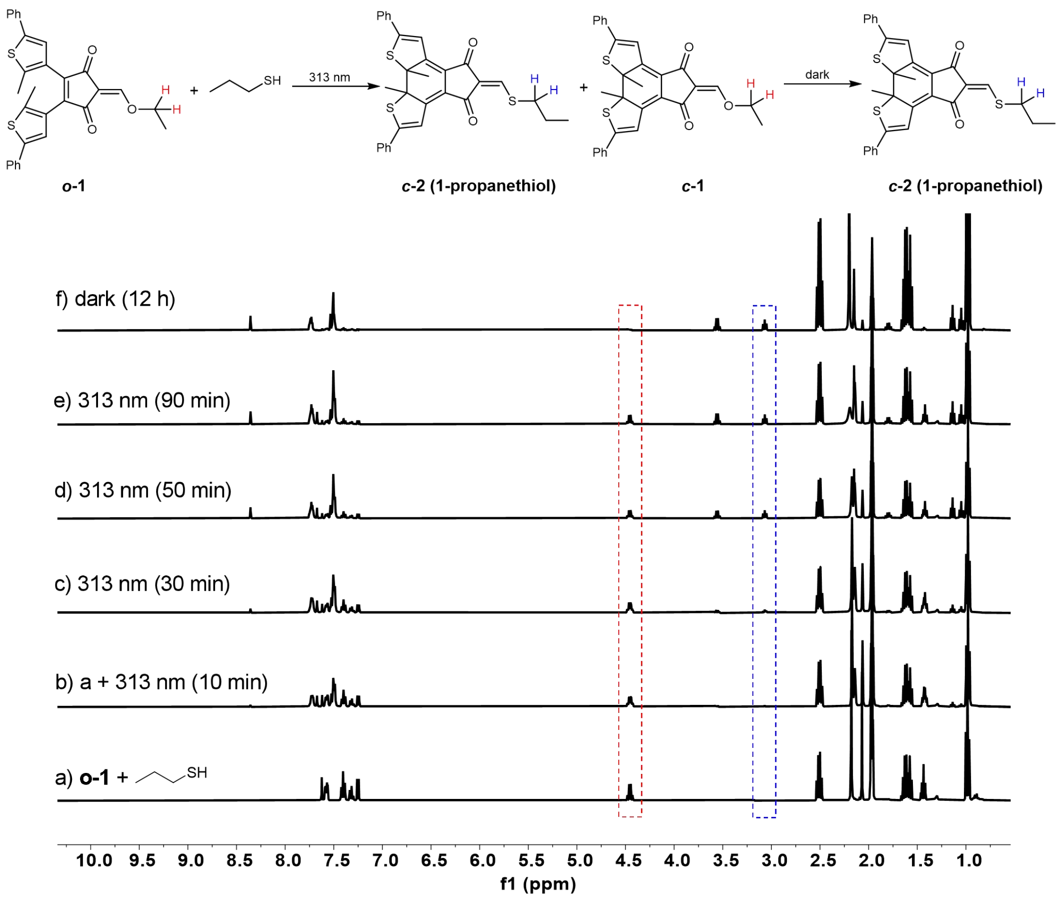

Supplementary Figure 39. (a-e) Stacked ^{1}H NMR (400 MHz, 20 °C) spectra of the reaction of ***o*-1** (5 mM) with 1-propanethiol (3 equiv.) in CD_3CN under UV light (313 nm) at varied time. The ratio of ***o*-1**:***c*-1**:***c*-2** is 6:57:37; (f) Continued reaction for 12 h in dark. The ratio of ***o*-1**:***c*-2** is 6:94.

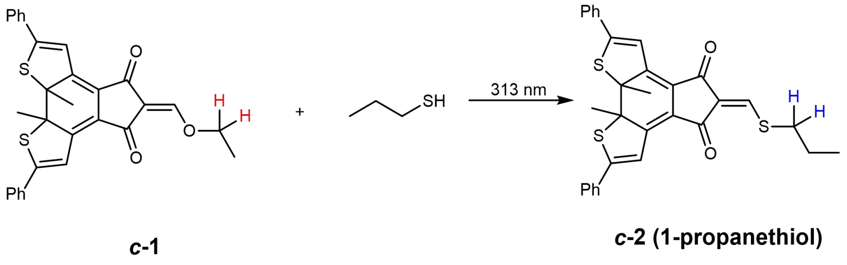

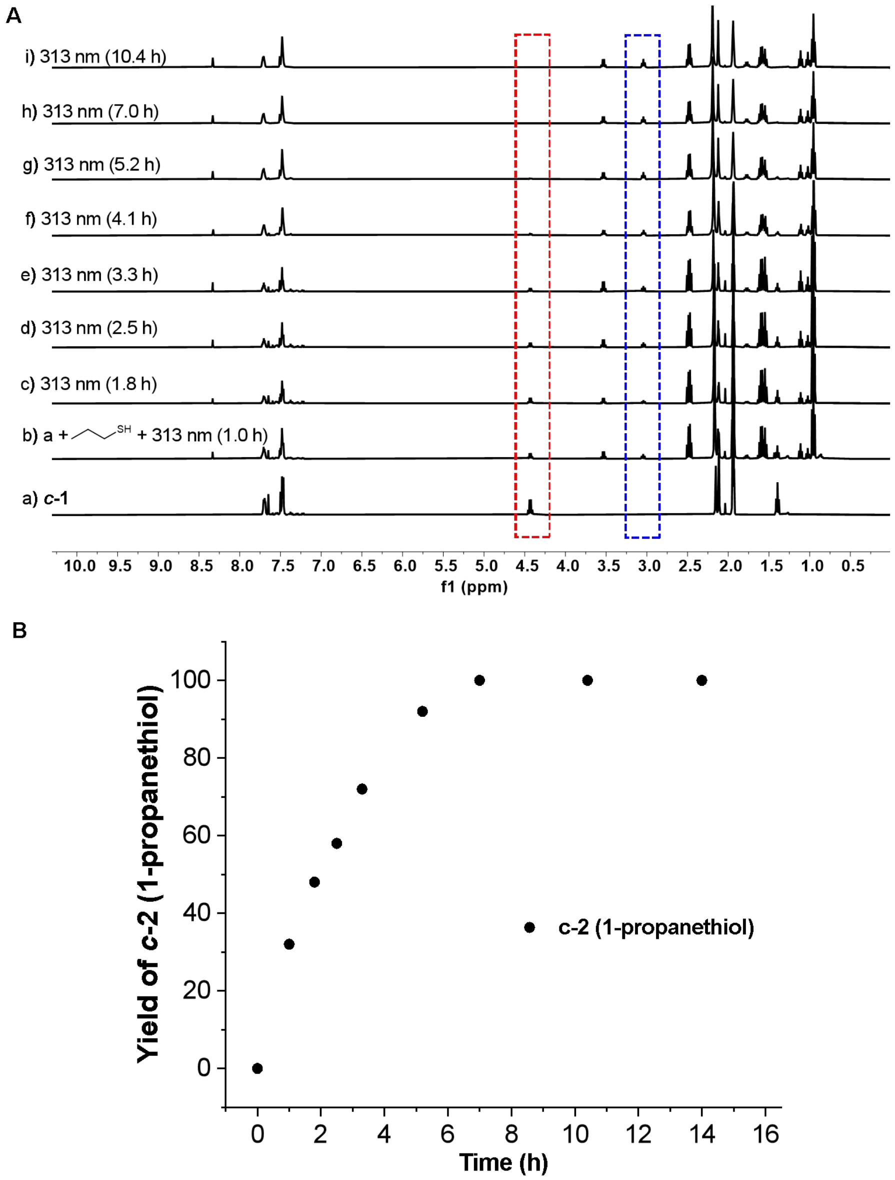

Supplementary Figure 40. (a-g) Stacked ^{1}H NMR spectra (400 MHz, 20 °C) of the reaction of ***c*-1** (5 mM) with 1-propanethiol (3 equiv.) in CD_3CN under UV light (313 nm) at varied time. (B) The kinetics profile of the reaction of ***c*-1** with 1-propanethiol in 15 h.

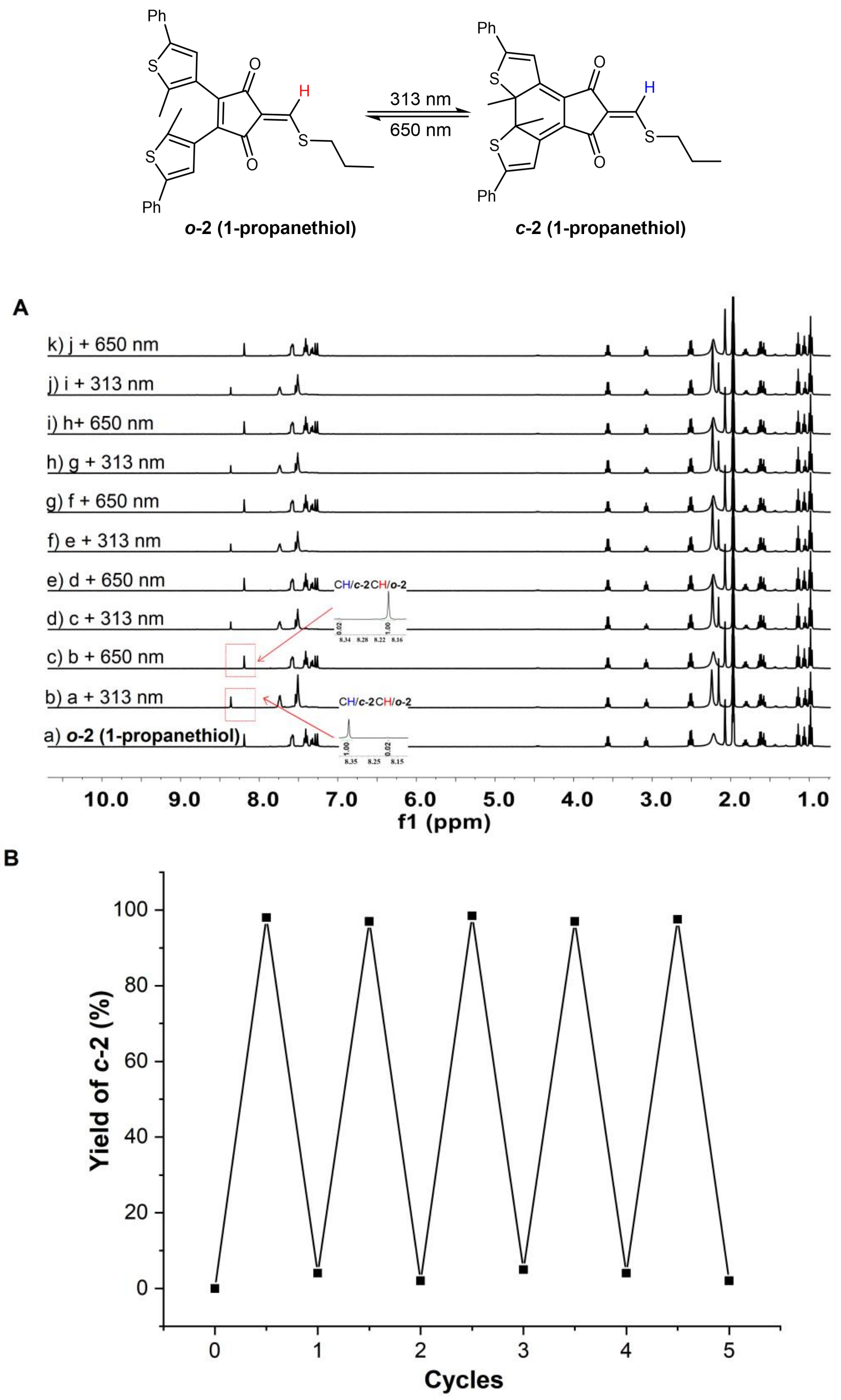

Supplementary Figure 41. (A) (a) ^{1}H NMR (400 MHz, 20 °C) spectrum of ***o*-2** created from **1** (5 mM) and 1-propanethiol (3.0 equiv.) *in situ* in CD_3CN; (b) Irradiation of ***o*-2** with UV light (313 nm, 1 h); The ratio of ***c*-2** and ***o*-2** is 98:2; (c) Further irradiation with visible light (650 nm, 1.5 h); The ratio of ***o*-2** and ***c*-2** is 98:2; (d-k) Continued photoswitching for four cycles. (B) Variation of the yield of ***o*-2** in photoswitching cycle monitored by ^{1}H NMR spectrum.

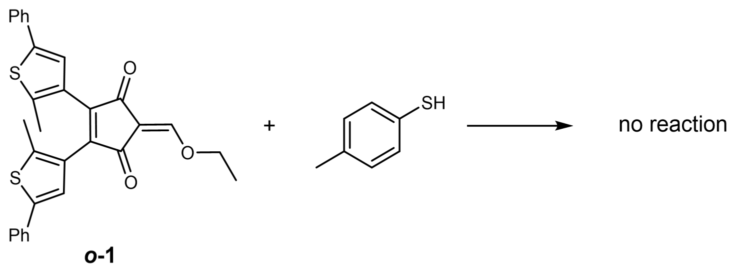

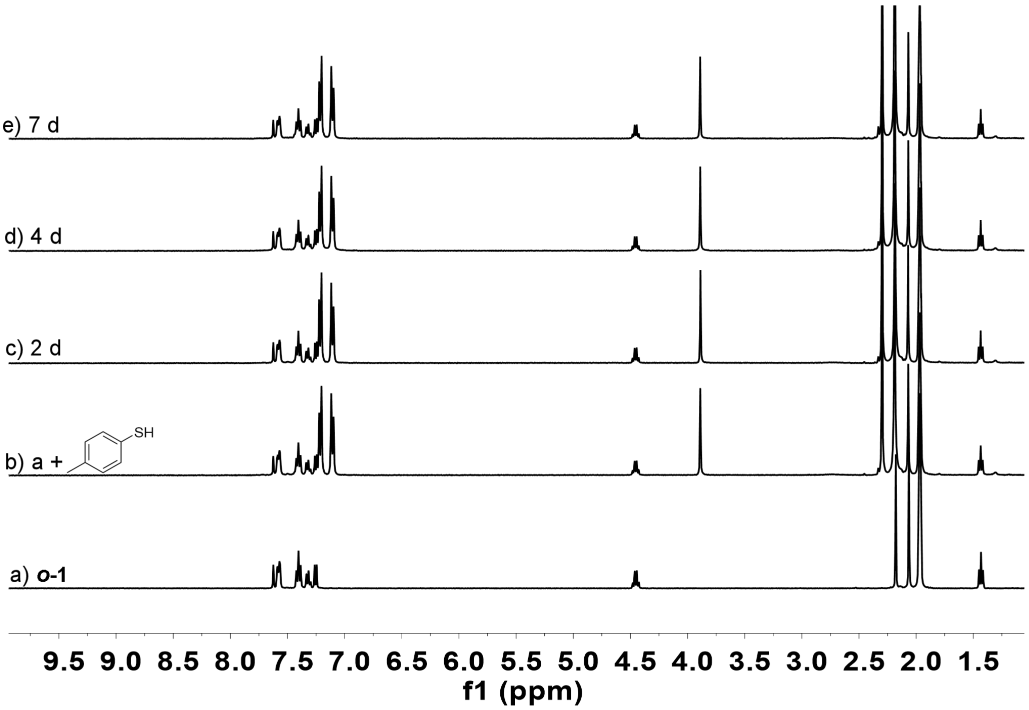

Supplementary Figure 42. Stacked ^{1}H NMR (400 MHz, 20 °C) spectra of the reaction of ***o*-1** (5 mM) with *p*-toluenethiol (3.0 equiv.) in CD_3CN at varied time. No reaction occurred.

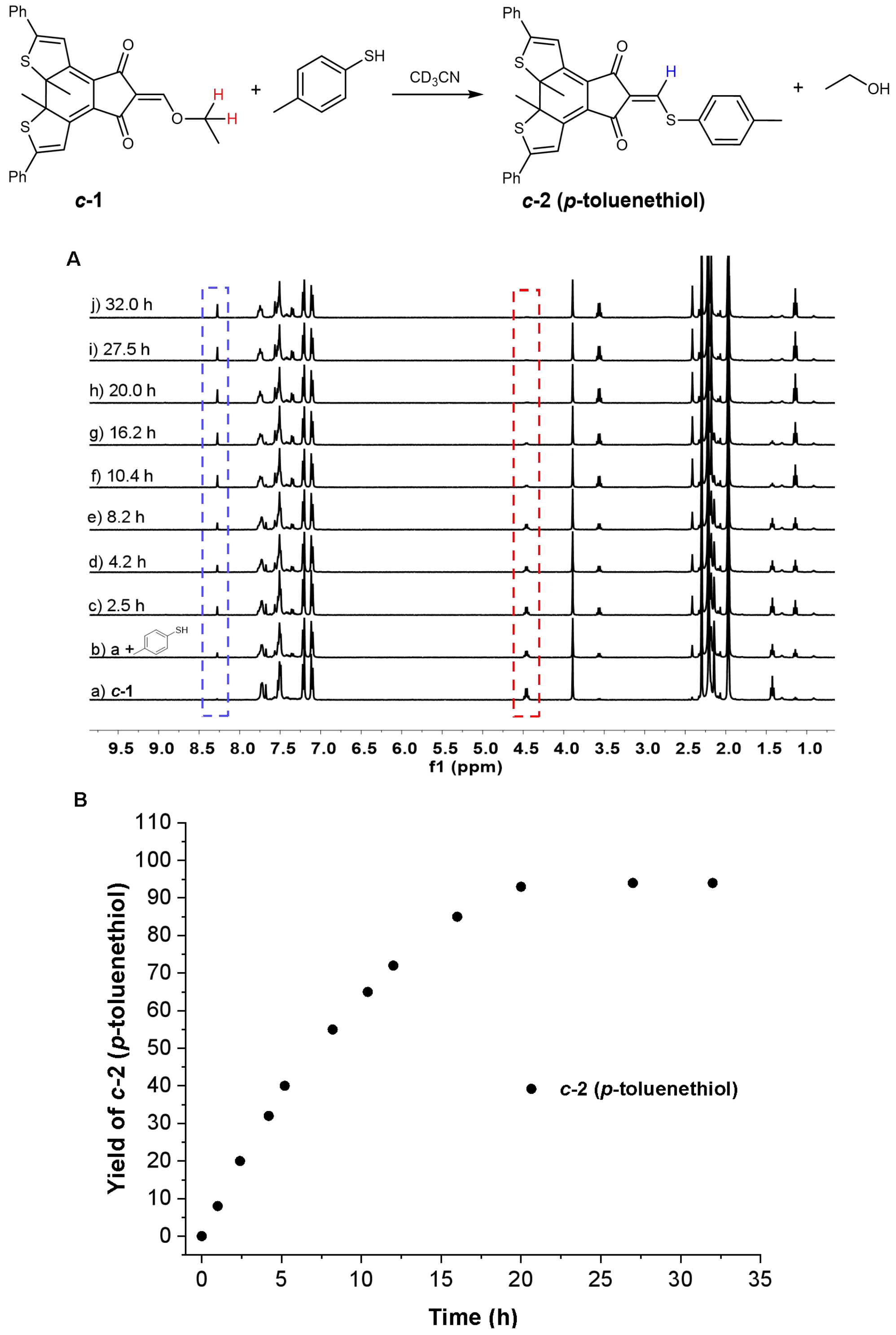

Supplementary Figure 43. (A) Stacked ^{1}H NMR (400 MHz, 20 °C) spectra of the reaction of ***c*-1** (5 mM) with *p*-toluenethiol (3 equiv.) in CD_3CN at varied time. (B) The kinetics profile of the reaction of ***c*-1** with *p*-toluenethiol in 32 h.

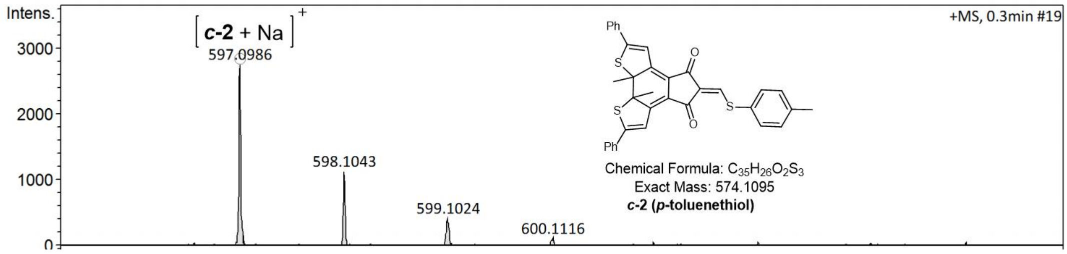

Supplementary Figure 44. ESI mass spectrum of the reaction of ***c*-1** and *p*-toluenethiol in acetonitrile.

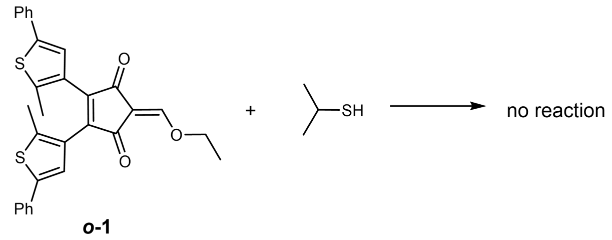

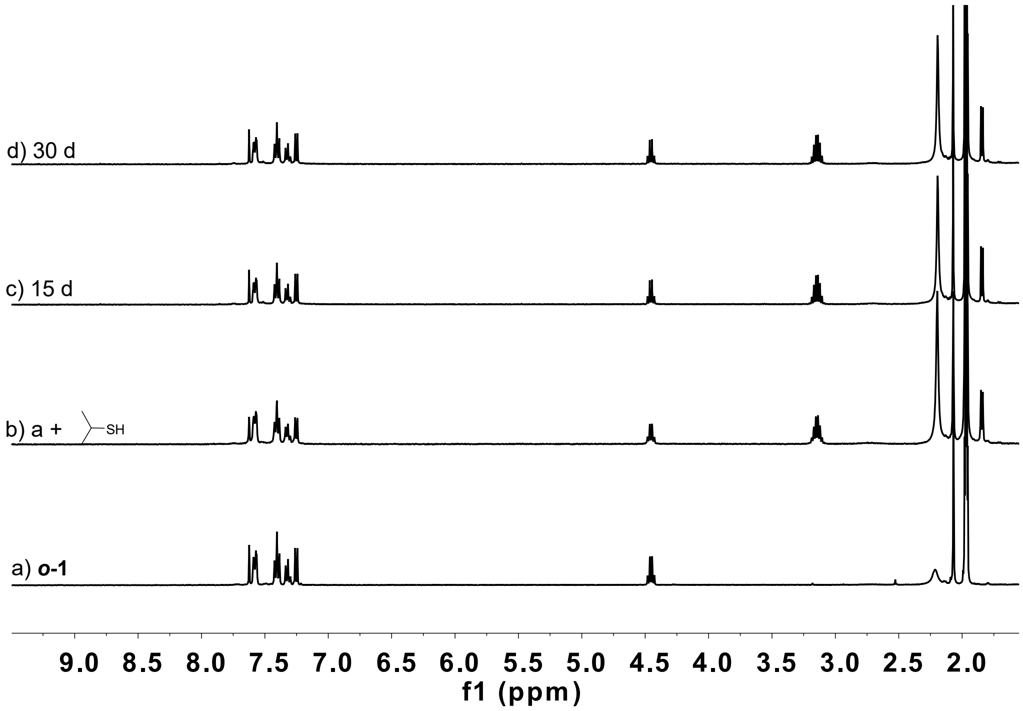

Supplementary Figure 45. Stacked ^{1}H NMR (400 MHz, 20 °C) spectra of the reaction of ***o*-1** (5 mM) with 2-propanethiol (3.0 equiv.) in CD_3CN at varied time. No reaction occurred.

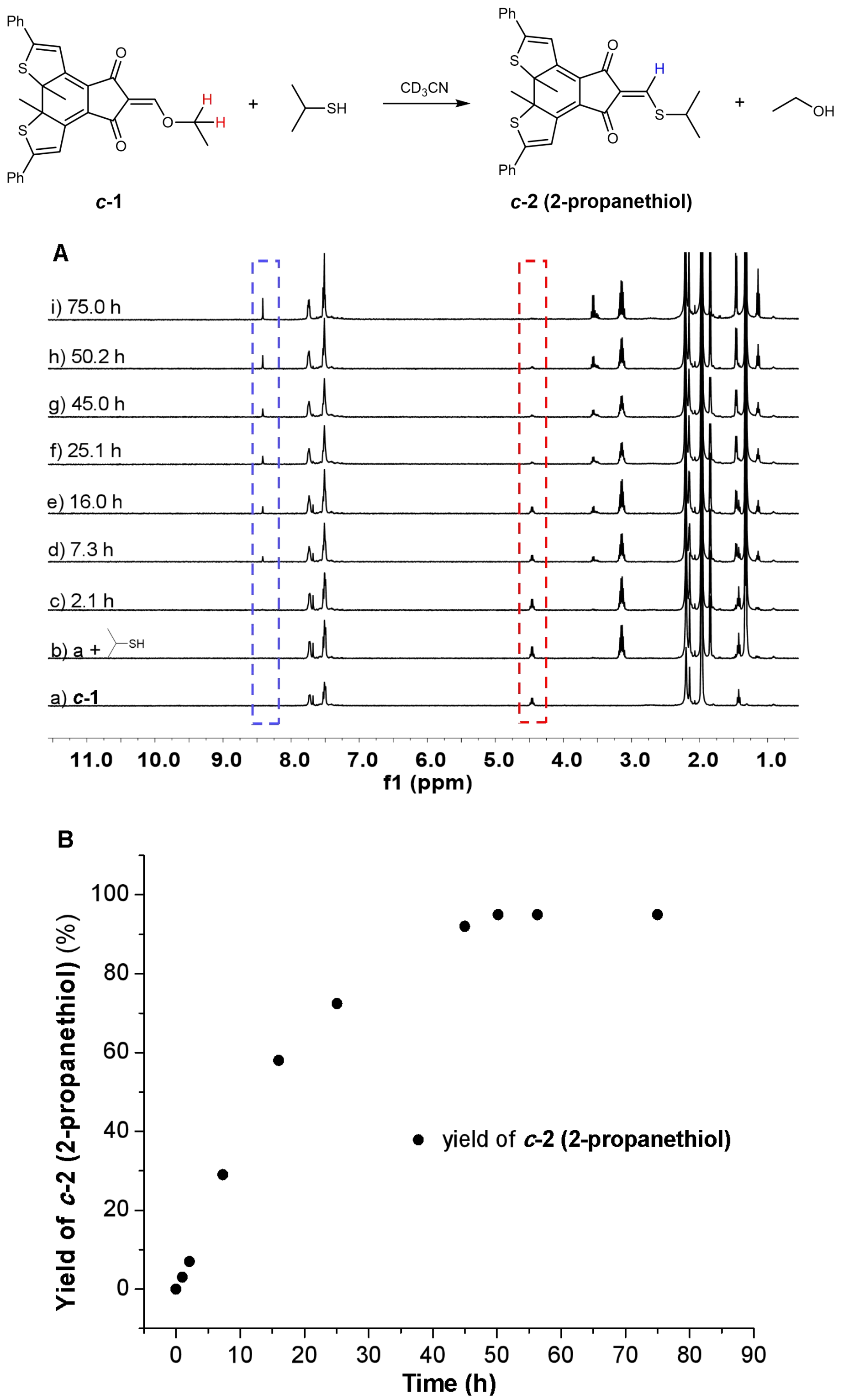

Supplementary Figure 46. (A) Stacked ^{1}H NMR (400 MHz, 20 °C) spectra of the reaction of ***c*-1** (5 mM) with 2-propanethiol (3 equiv.) in CD_3CN at varied time. (B) The kinetics profile of the reaction of ***c*-1** with 2-propanethiol in 75 h.

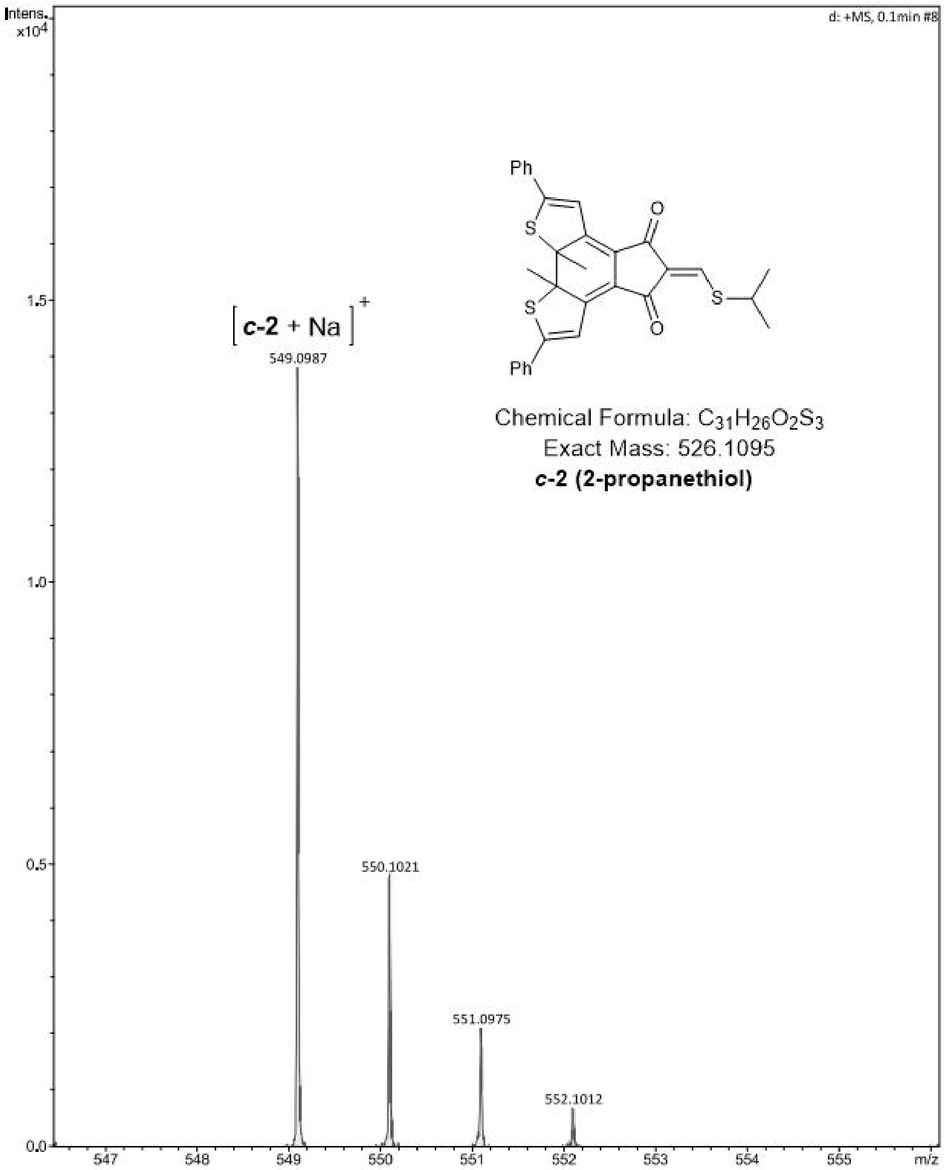

Supplementary Figure 47. ESI mass spectrum of the reaction of ***c*-1** and 2-propanethiol in acetonitrile.

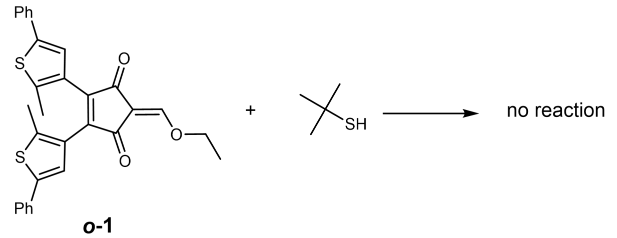

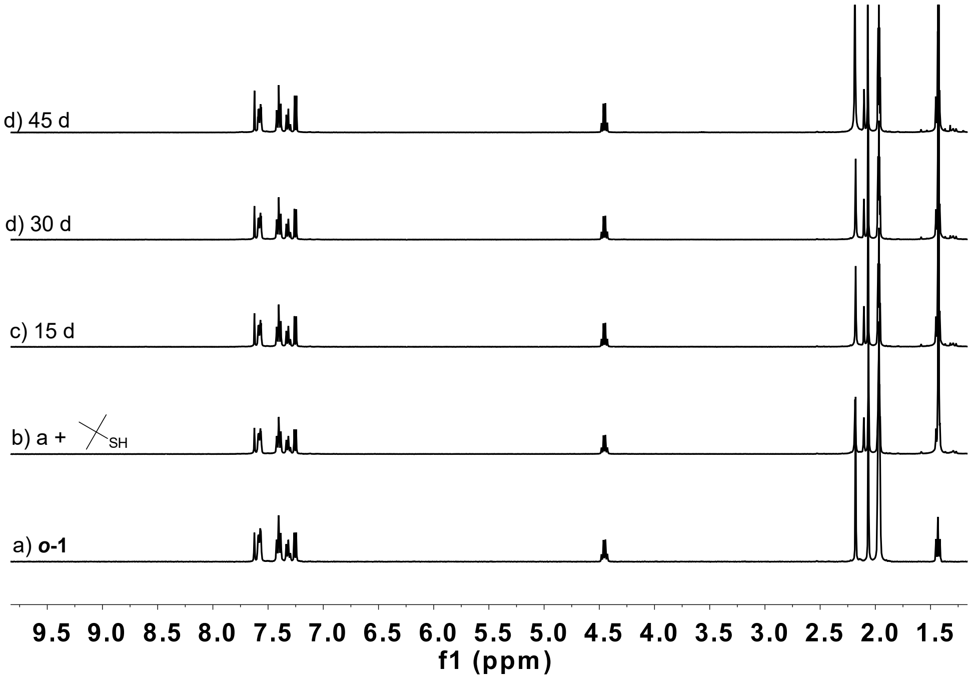

Supplementary Figure 48. Stacked ^{1}H NMR (400 MHz, 20 °C) spectra of the reaction of ***o*-1** (5 mM) with *t*-butyl mercaptan (3.0 equiv.) in CD_3CN at varied time. No reaction occurred.

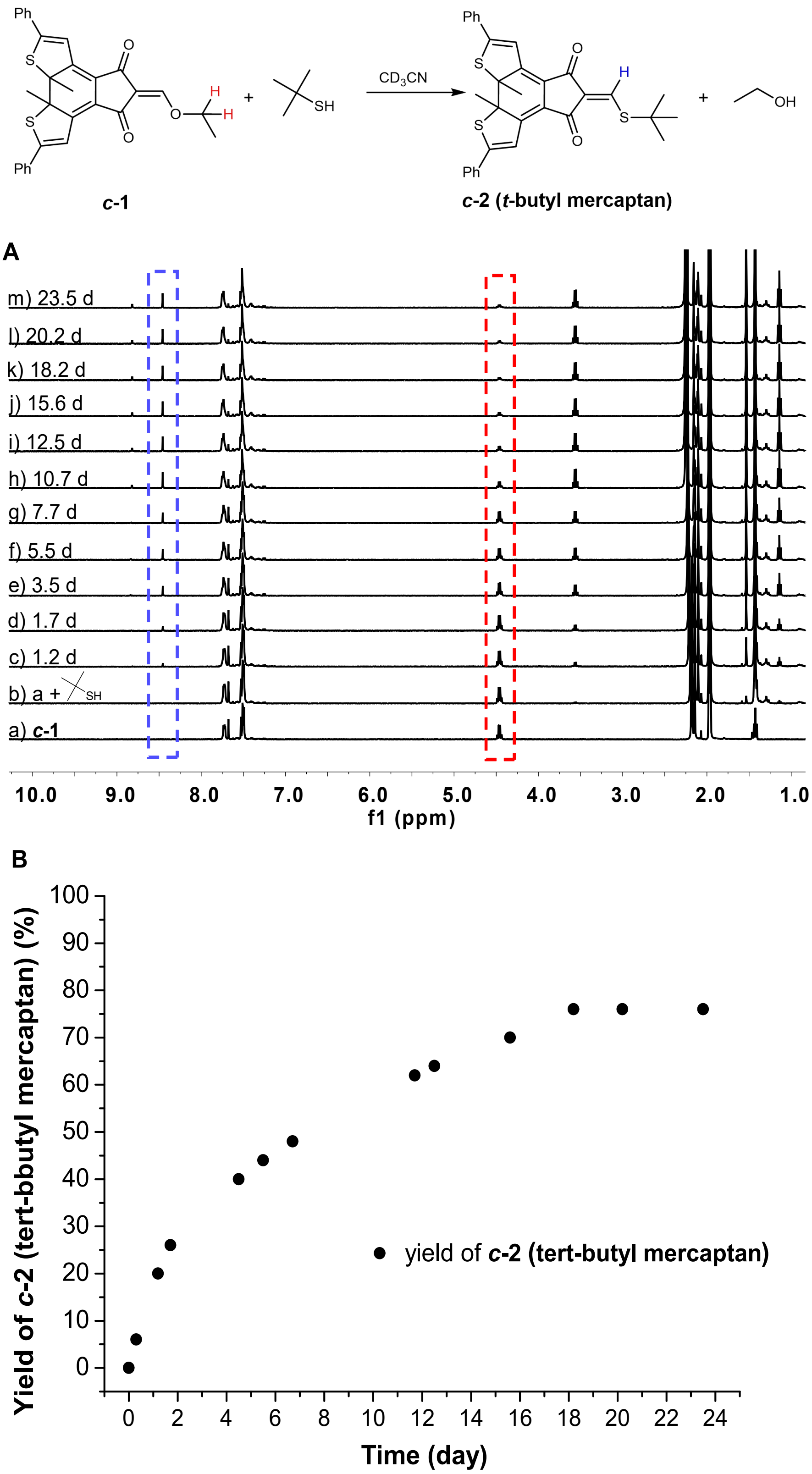

Supplementary Figure 49. (A) Stacked ^{1}H NMR (400 MHz, 20 °C) spectra of the reaction of ***c*-1** (5 mM) with *t*-butyl mercaptan (3 equiv.) in CD_3CN at varied time. (B) The kinetics profile of the reaction of ***c*-1** with *t*-butyl mercaptan in 24 d.

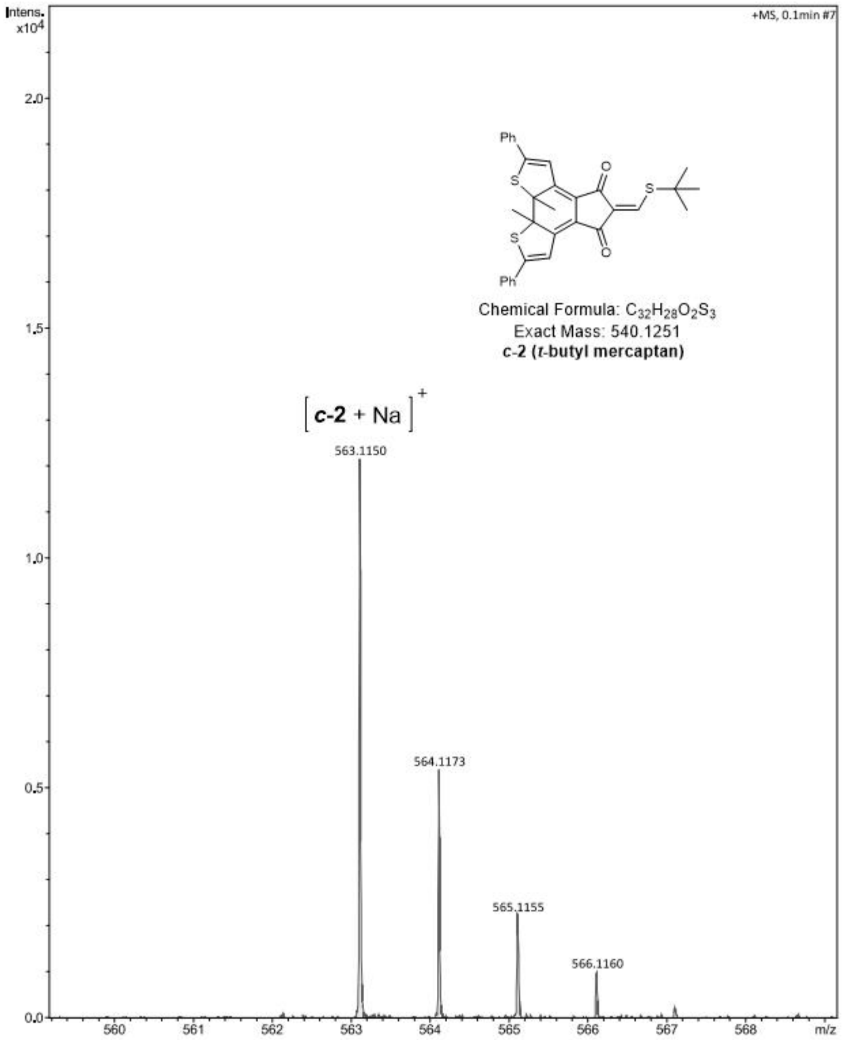

Supplementary Figure 50. ESI mass spectrum of the reaction of ***c*-1** and *t*-butyl mercaptan in acetonitrile.

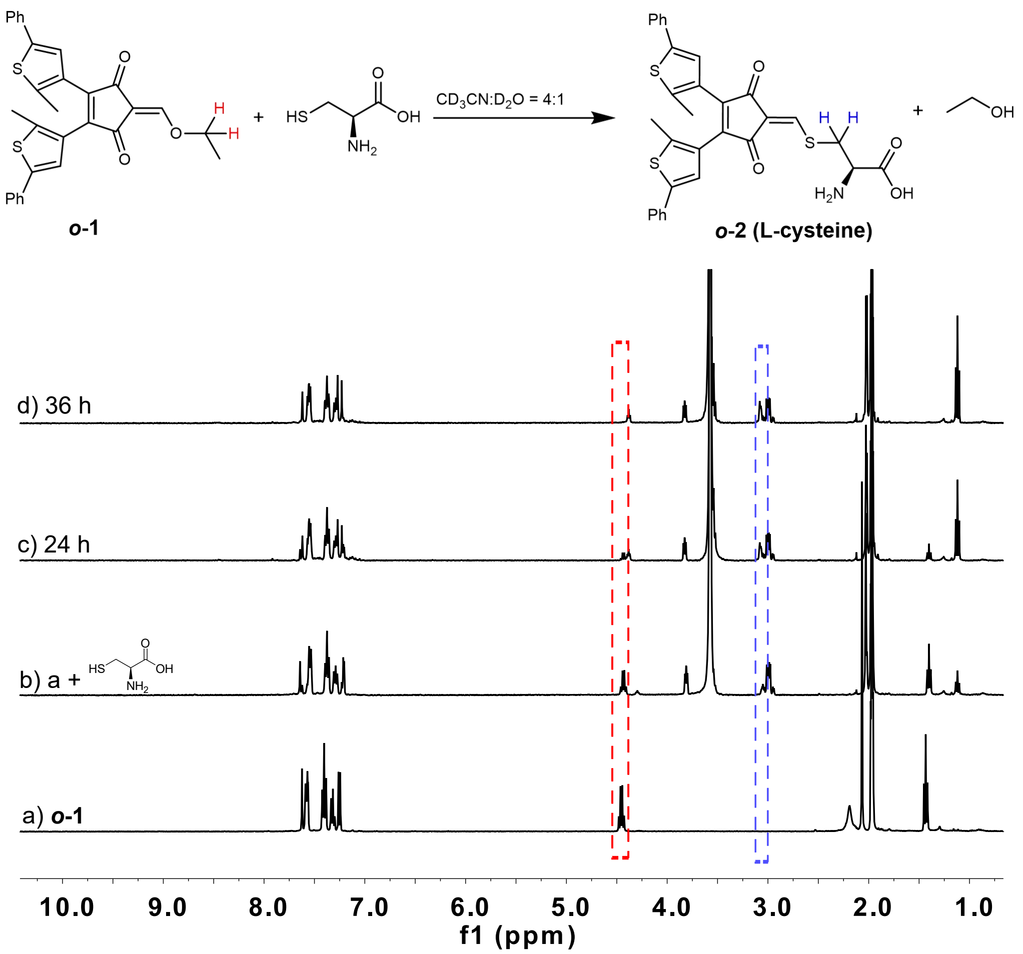

Supplementary Figure 51. (a) ^{1}H NMR (400 MHz, 20 °C) spectrum of ***o*-1** (5 mM) in CD_3CN (0.5 mL); (b) After addition of L-cysteine (3 equiv. in D_2O (0.125 mL)) into the panel a; (c, d) The reaction of ***o*-1** with L-cysteine after 24 h and 36 h.

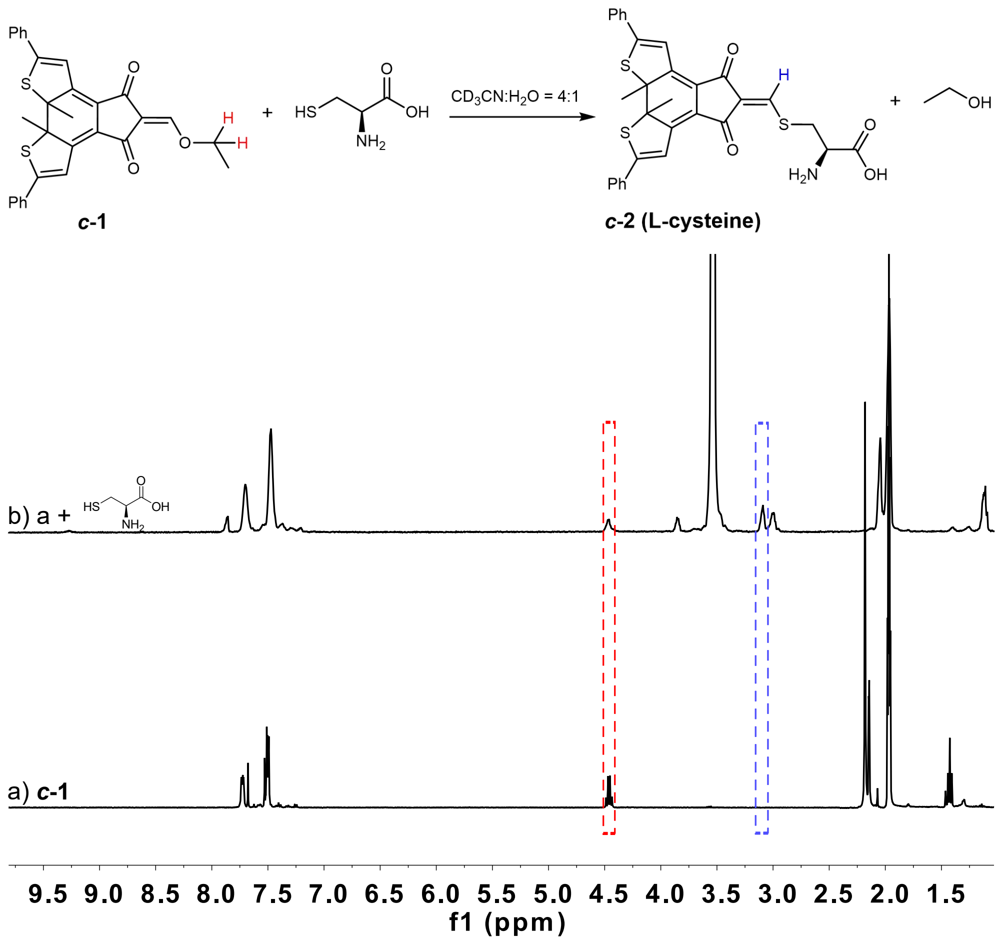

Supplementary Figure 52. (a) ^{1}H NMR (400 MHz, 20 °C) spectrum of ***c*-1** (5 mM) in CD_3CN (0.5 mL); (b) After addition of L-cysteine (3 equiv. in D_2O (0.125 mL)) into the panel a. The reaction was complete within 3 min to afford ***c*-2**.

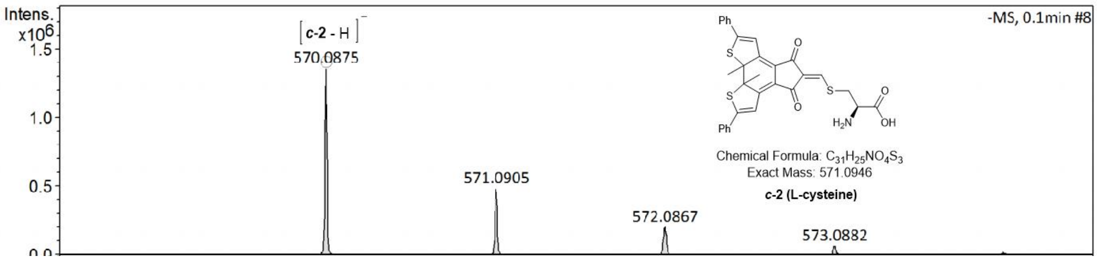

Supplementary Figure 53. ESI mass spectrum of the reaction of ***c*-1** and L-cysteine in 4:1 $MeCN:H_2O$.

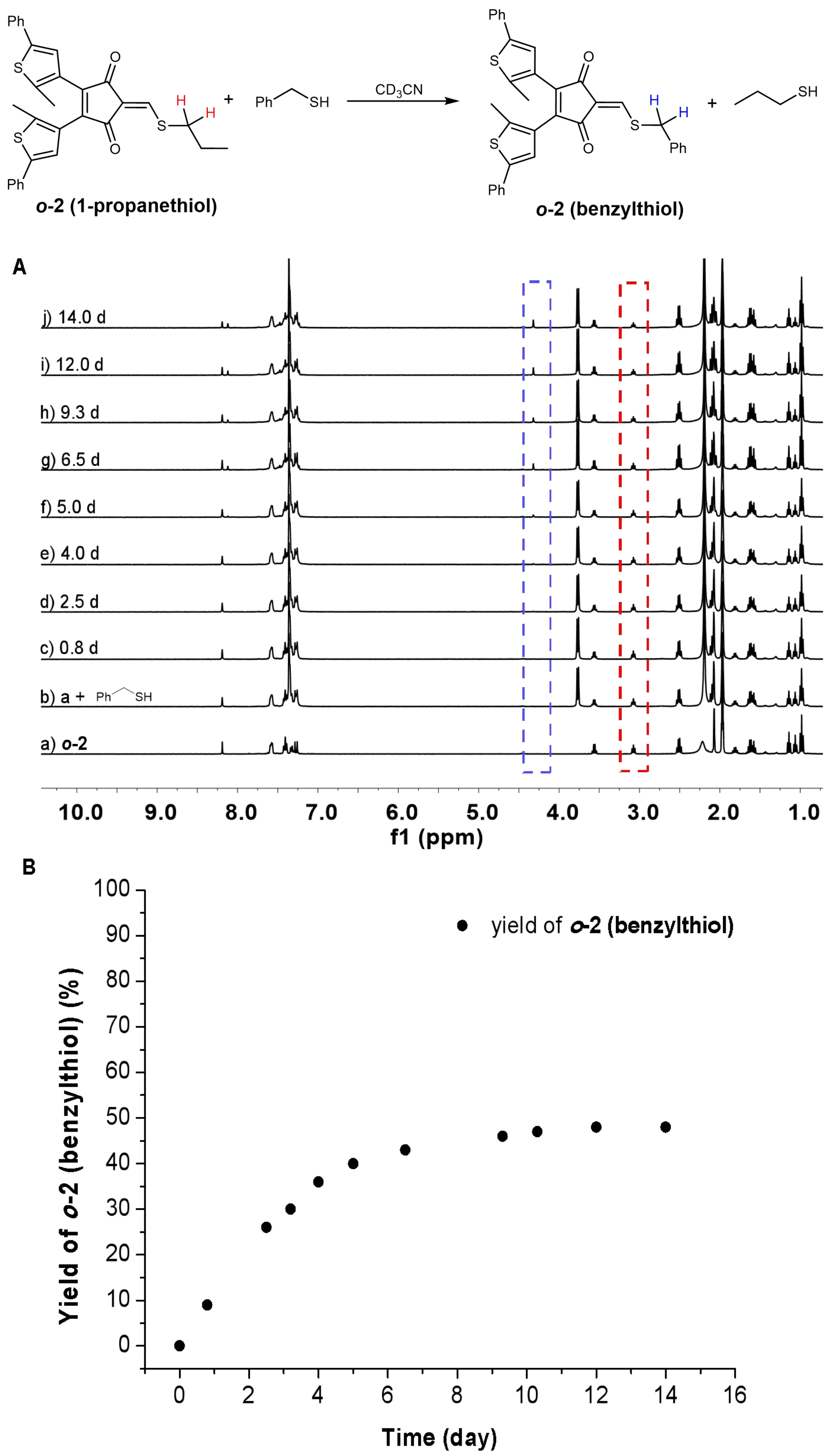

Supplementary Figure 54. (A) Stacked ^{1}H NMR (400 MHz, 20 °C) spectra of the reaction of ***o*-2** created from **1** (5 mM) and 1-propanethiol (3.0 equiv.) *in situ* with benzylthiol (3 equiv.) in CD_3CN at varied time. (B) The kinetics profile of the reaction of 1-propanethiol derived ***o*-2** with benzylthiol in 14 d.

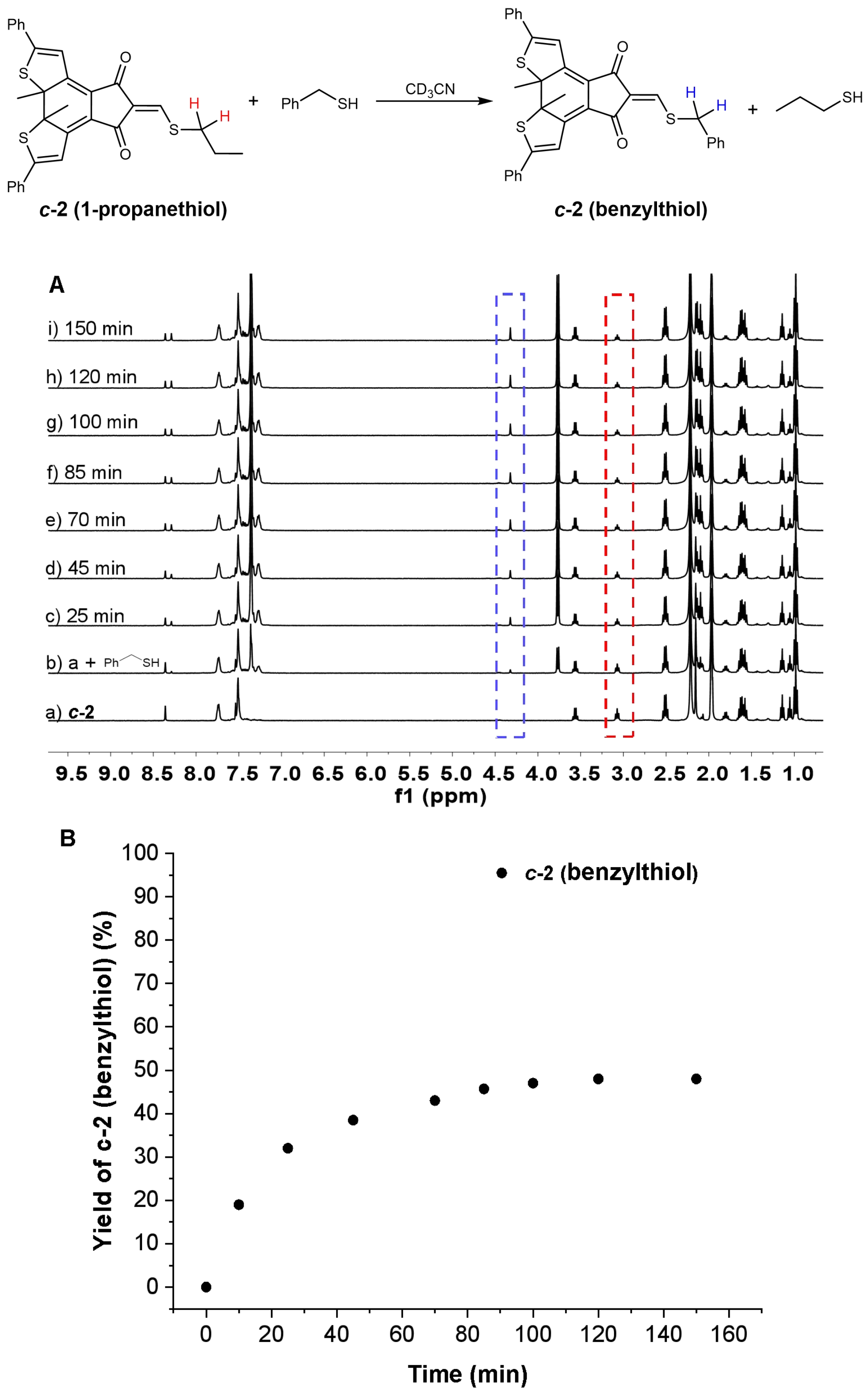

Supplementary Figure 55. (A) Stacked ^{1}H NMR (400 MHz, 20 °C) spectra of the reaction of ***c*-2** created from **1** (5 mM) and 1-propanethiol (3.0 equiv.) *in situ* with benzylthiol (3 equiv.) in CD_3CN at varied time. (B) The kinetics profile of the reaction of 1-propanethiol derived ***c*-2** with benzylthiol in 150 min.

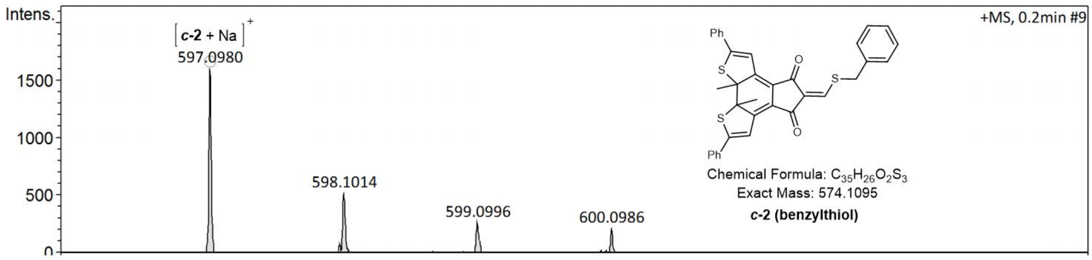

Supplementary Figure 56. ESI mass spectrum of the reaction of 1-propanethiol derived ***c*-2** with benzylthiol in acetonitrile.

5. Light-Controlled Exchange of Amine Nucleophiles.

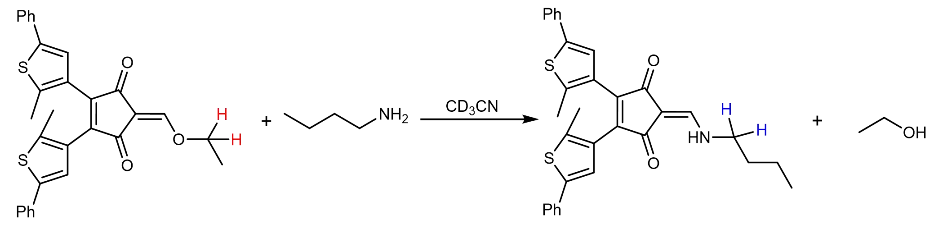

***o*-1** **o-3 (1-butylamine)**

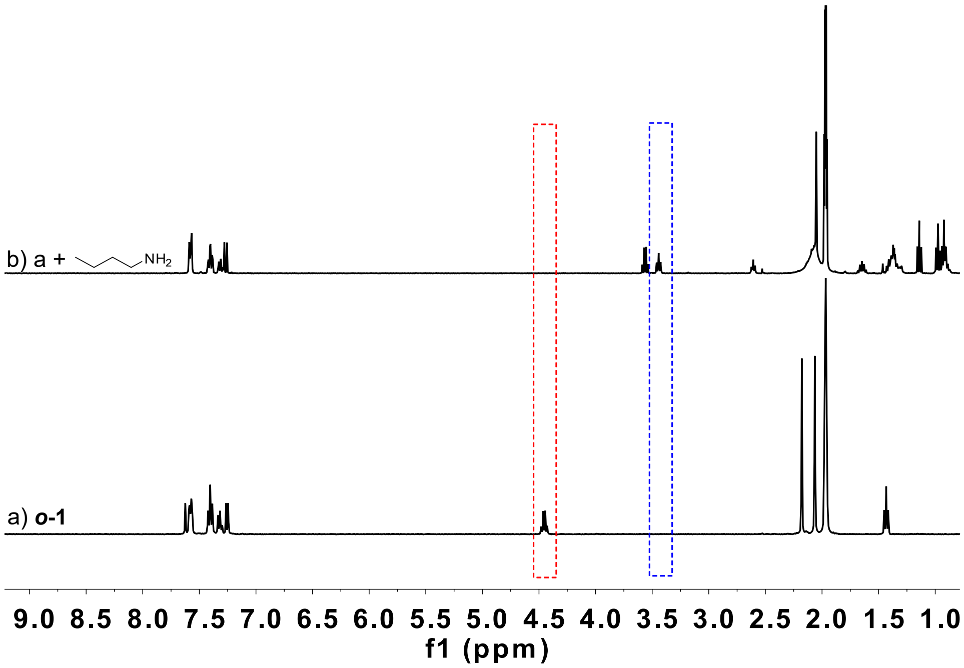

Supplementary Figure 57. ^{1}H NMR (400 MHz, 20 °C) spectrum of the reaction of ***o*-1** (5 mM) with 1-butylamine (1.5 equiv.) in CD_3CN after 3 min.

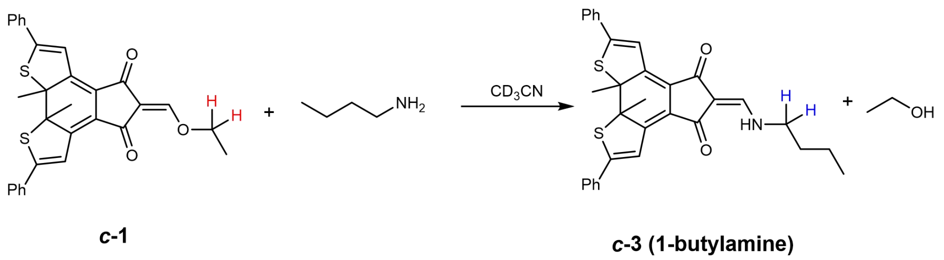

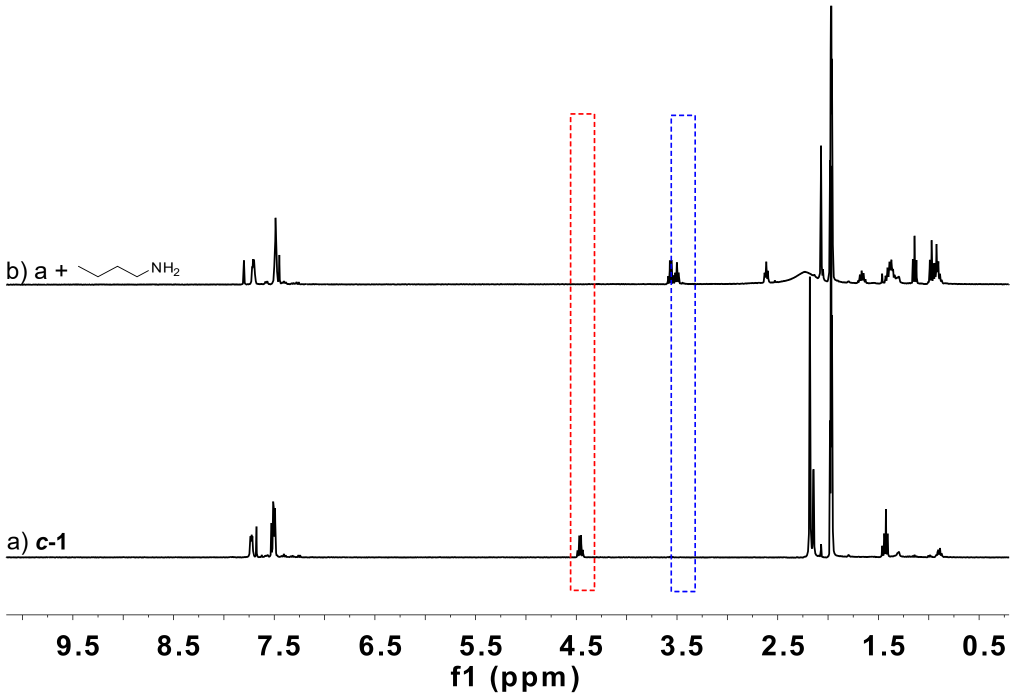

Supplementary Figure 58. ^{1}H NMR (400 MHz, 20 °C) spectrum of the reaction of ***c*-1** (5 mM) with 1-butylamine (1.5 equiv.) in CD_3CN after 3 min.

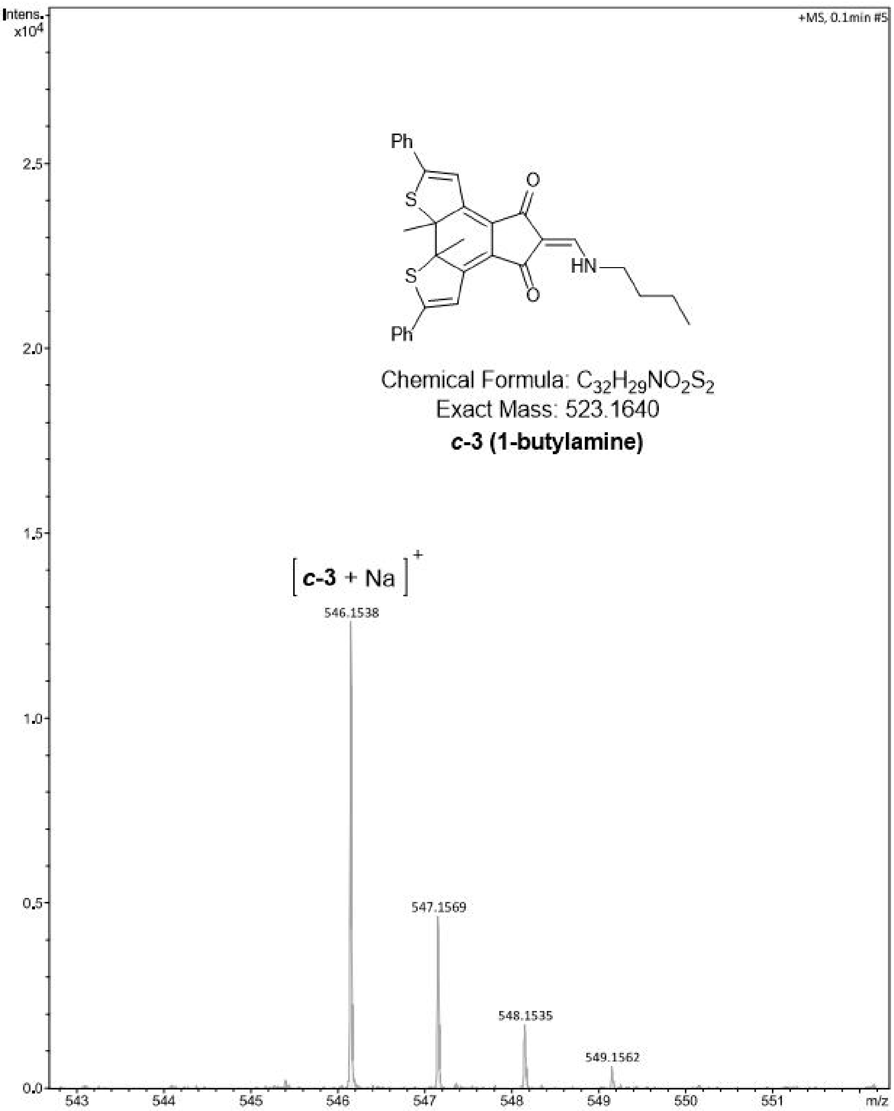

Supplementary Figure 59. ESI mass spectrum of the reaction of ***c*-1** and 1-butylamine in acetonitrile.

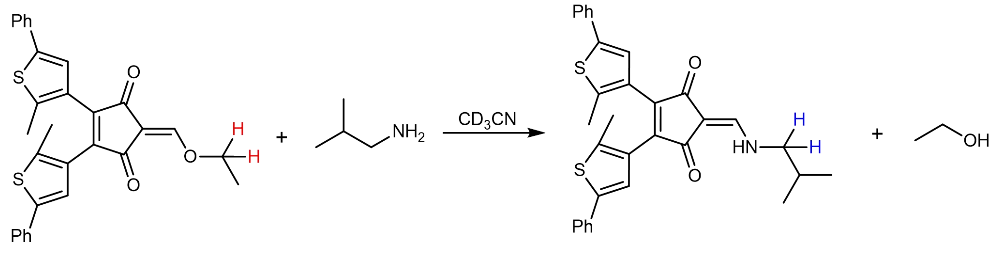

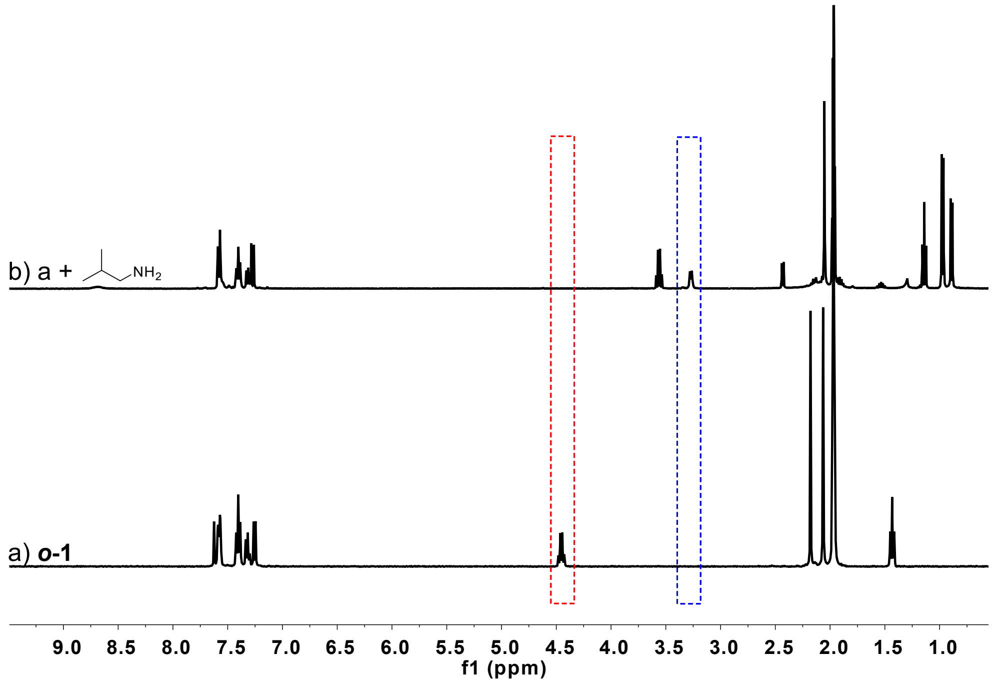

Supplementary Figure 60. (a) ^{1}H NMR (400 MHz, 20 °C) spectrum of ***o*-1** in CD_3CN; (b) The addition of isobutylamine (1.5 equiv.) into the panel a and the reaction after 3 min.

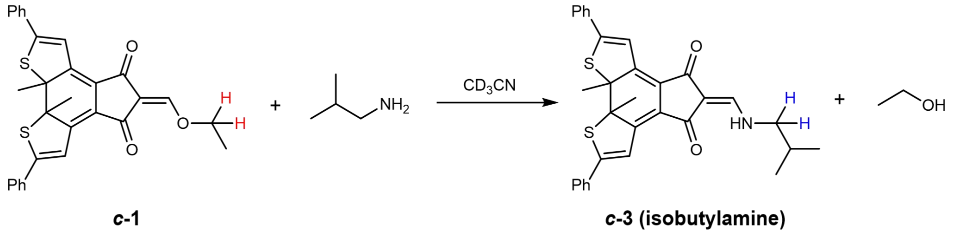

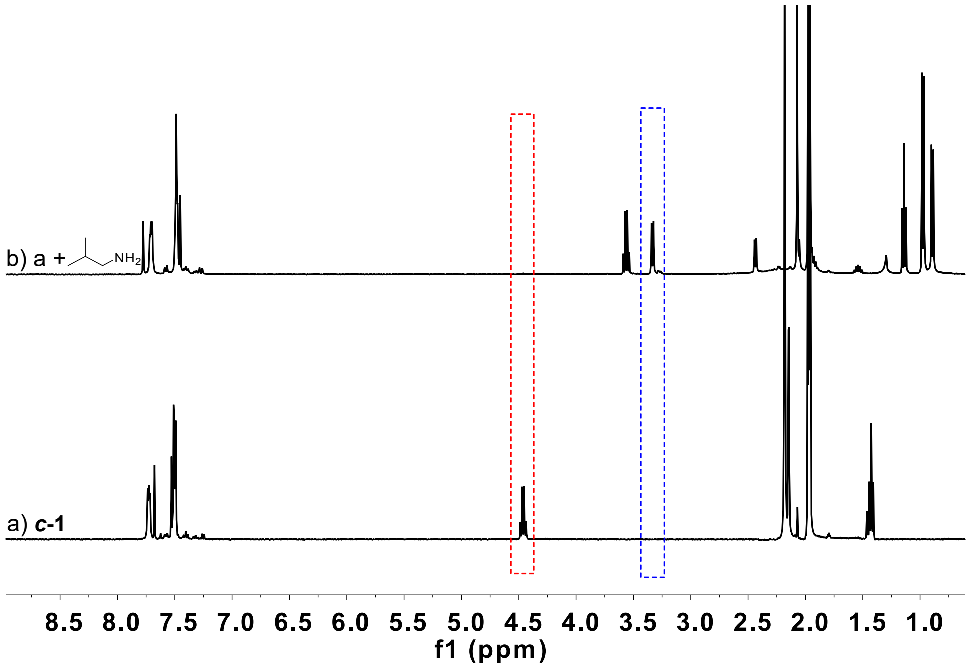

Supplementary Figure 61. (a) ^{1}H NMR (400 MHz, 20 °C) spectrum of ***c*-1** in CD_3CN; (b) The addition of isobutylamine (1.5 equiv.) into the panel a and the reaction after 3 min.

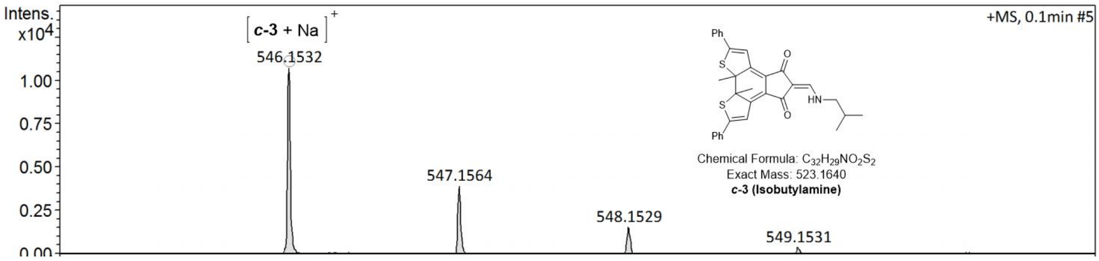

Supplementary Figure 62. ESI mass spectrum of the reaction of ***c*-1** and isobutylamine in acetonitrile.

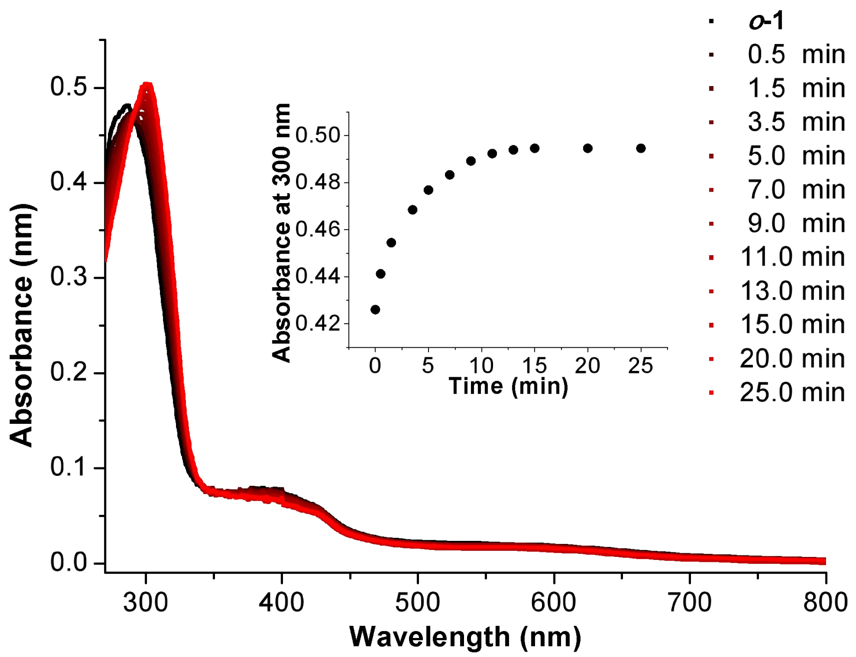

Supplementary Figure 63. Absorbance spectra of the reaction of ***o*-1** (25 μM, 25 °C) with 1-butylamine (1.5 equiv.) in CH_3CN. Inset: the kinetics profile of the reaction of ***o*-1** with 1-butylamine in 25 min.

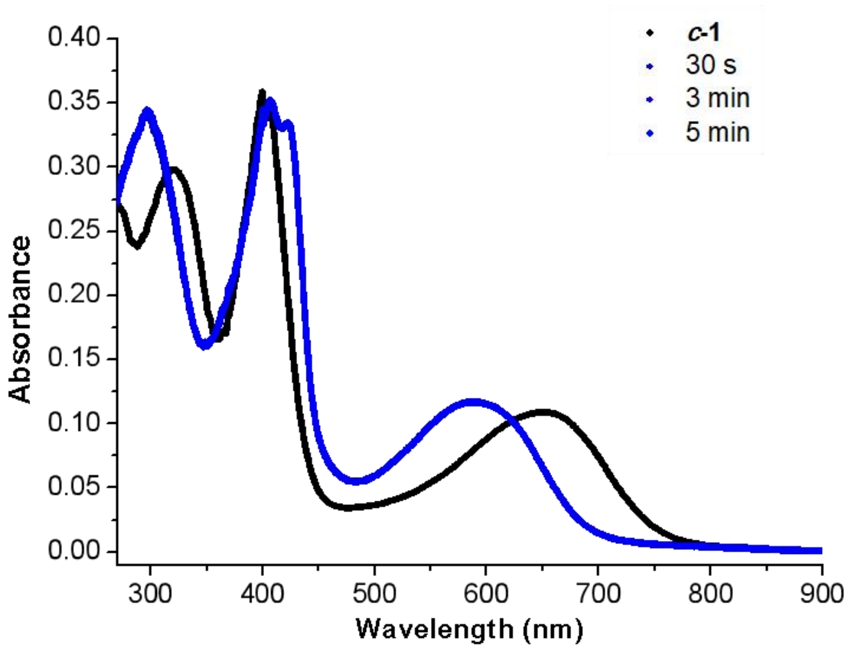

Supplementary Figure 64. Absorbance spectra of the reaction of ***c*-1** (25 μM, 25 °C) with 1-butylamine (1.5 equiv.) in CH_3CN. The reaction was complete after 30 s to afford ***c*-3**.

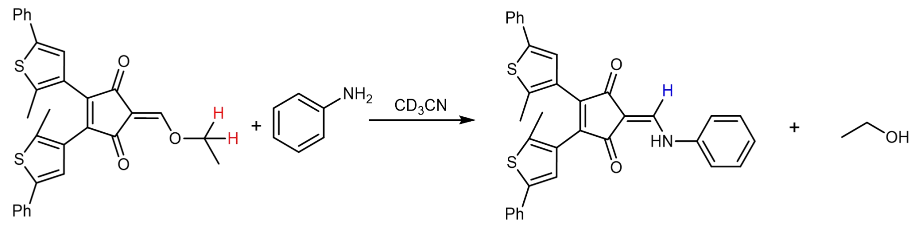

o-1 *o*-3 (aniline)

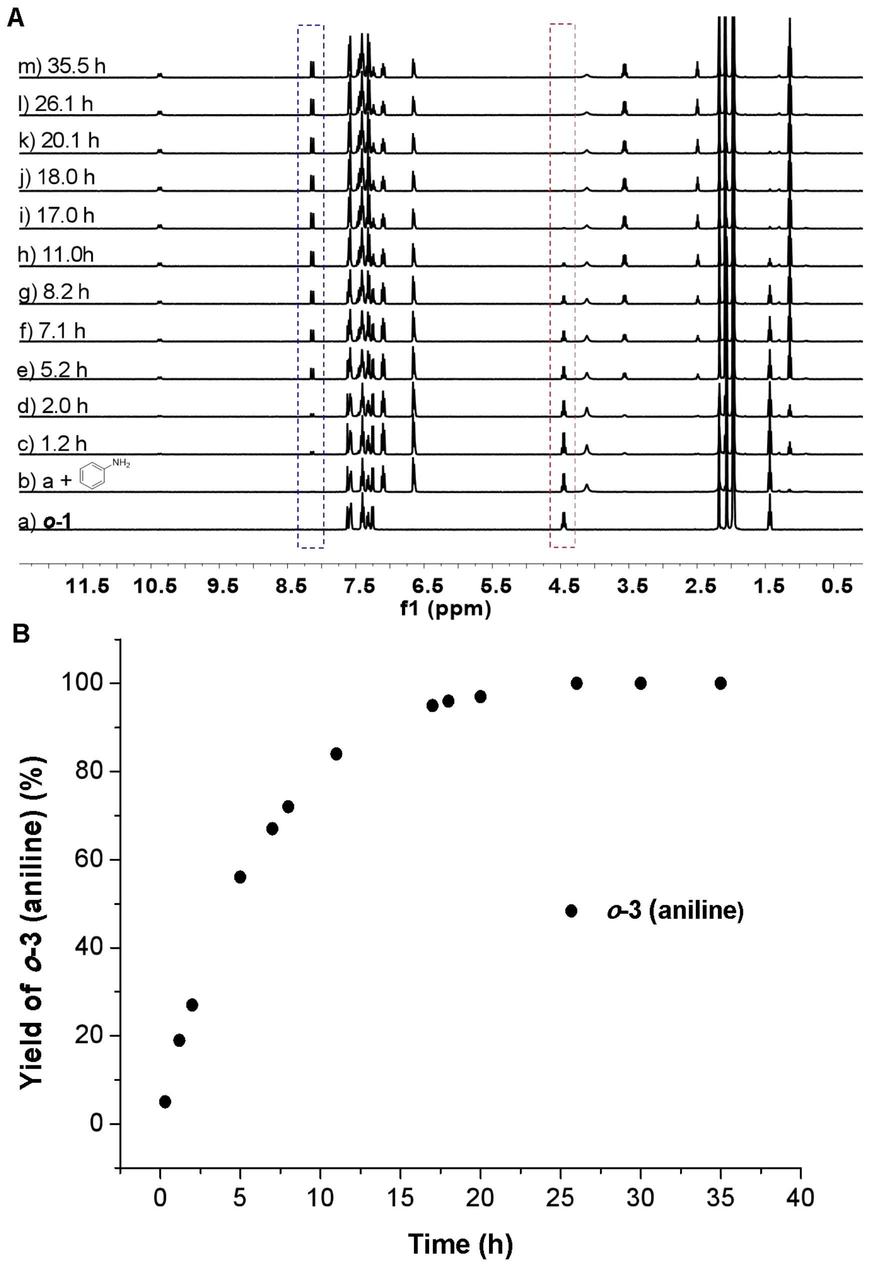

Supplementary Figure 65. (A) Stacked ^{1}H NMR (400 MHz, 20 °C) spectra of the reaction of ***o*-1** (5 mM) with aniline (1.5 equiv.) in CD_3CN at varied time. (B) The kinetics profile of the reaction of ***o*-1** with aniline in 35 h.

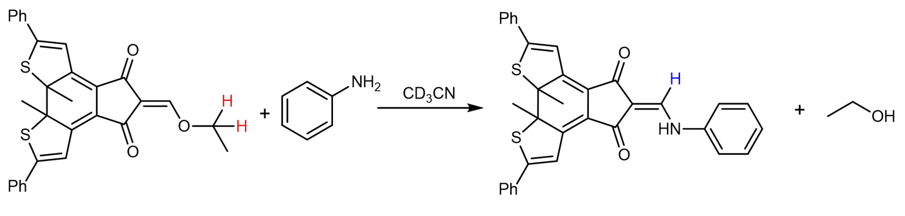

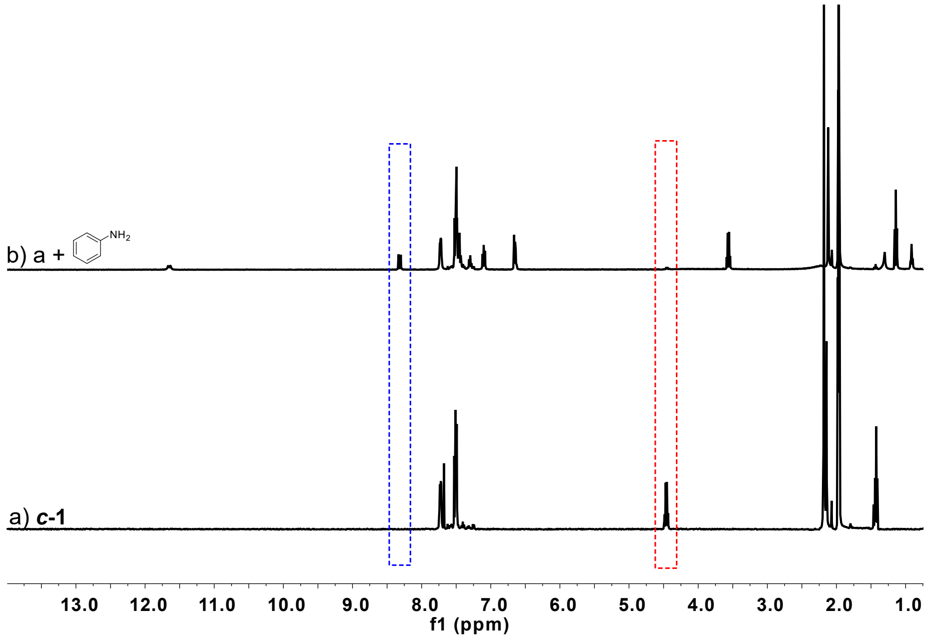

Supplementary Figure 66. (a) ^{1}H NMR (400 MHz, 20 °C) spectrum of ***c*-1** in CD_3CN; (b) The addition of aniline (1.5 equiv.) into the panel a and the reaction after 3 min.

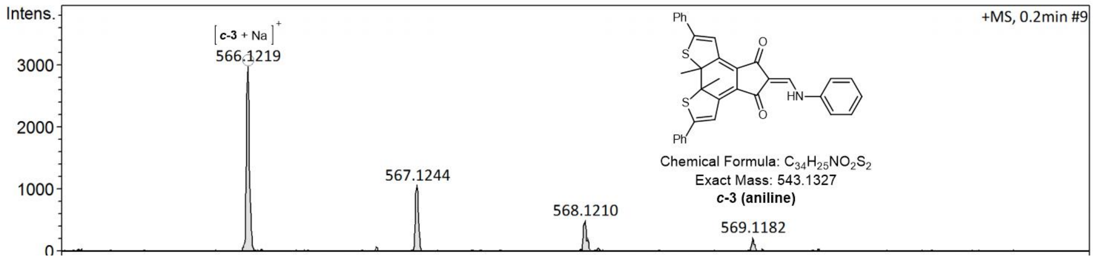

Supplementary Figure 67. ESI mass spectrum of the reaction of ***c*-1** and aniline in acetonitrile.

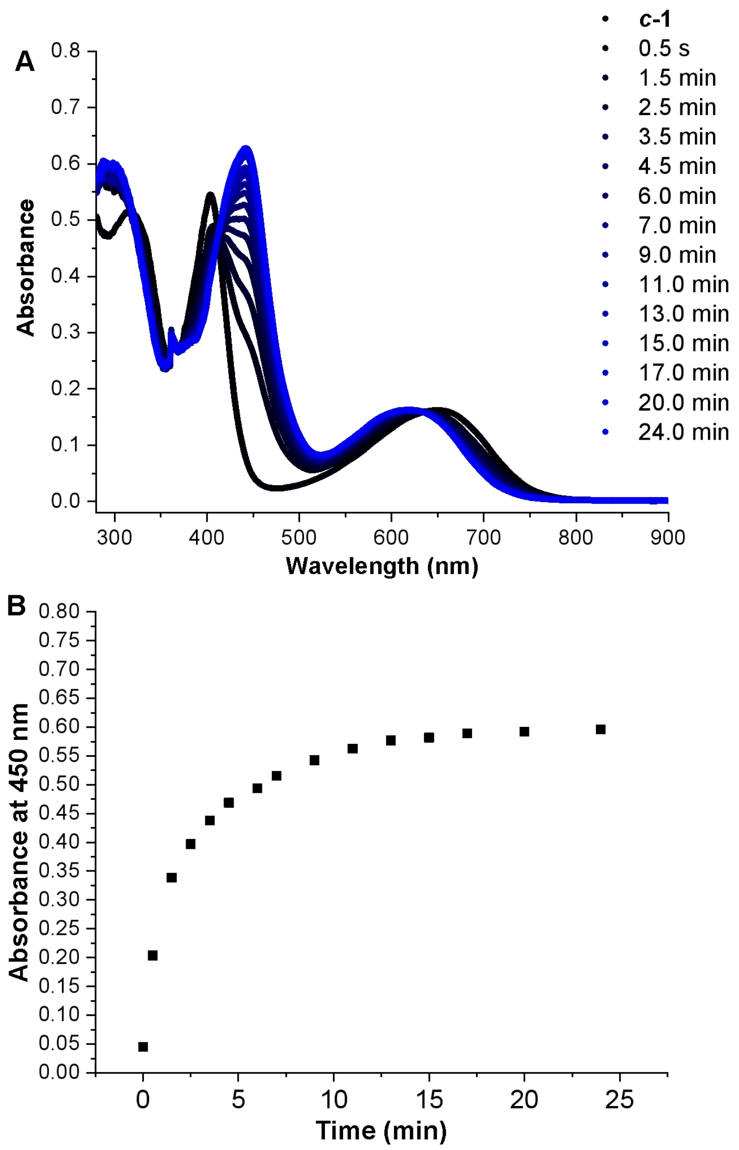

Supplementary Figure 68. (A) Absorbance spectra of the reaction of ***c*-1** (25 µM, 25 °C) with aniline (1.5 equiv.) in CH_3CN. (B) The kinetics profile of the reaction of ***c*-1** with aniline in 25 min.

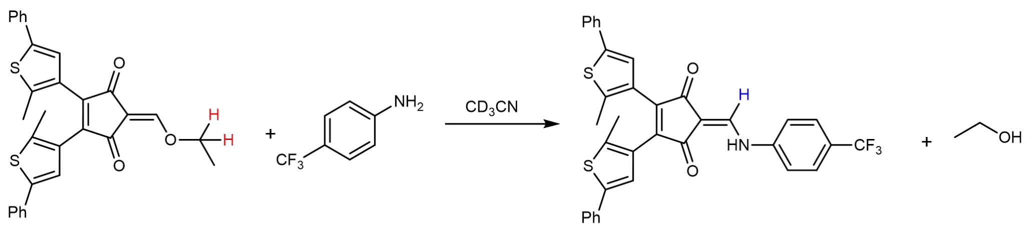

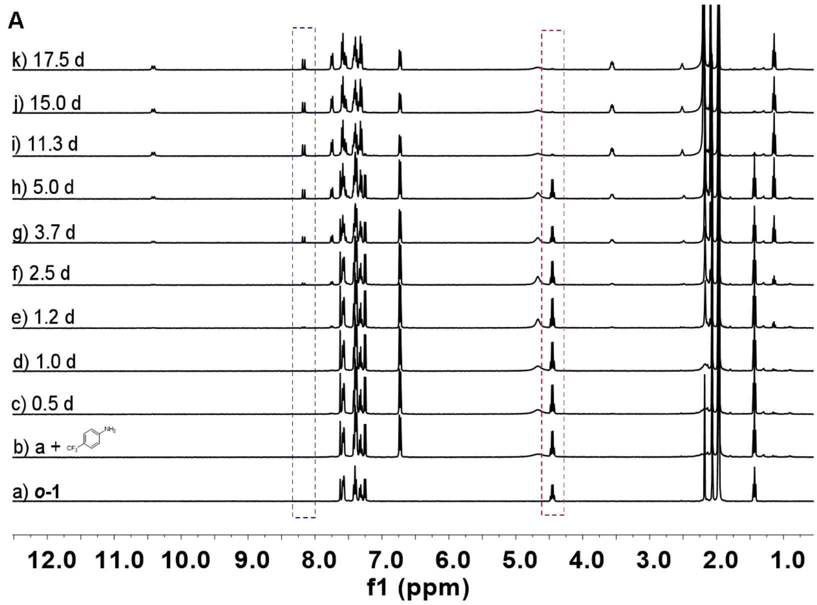

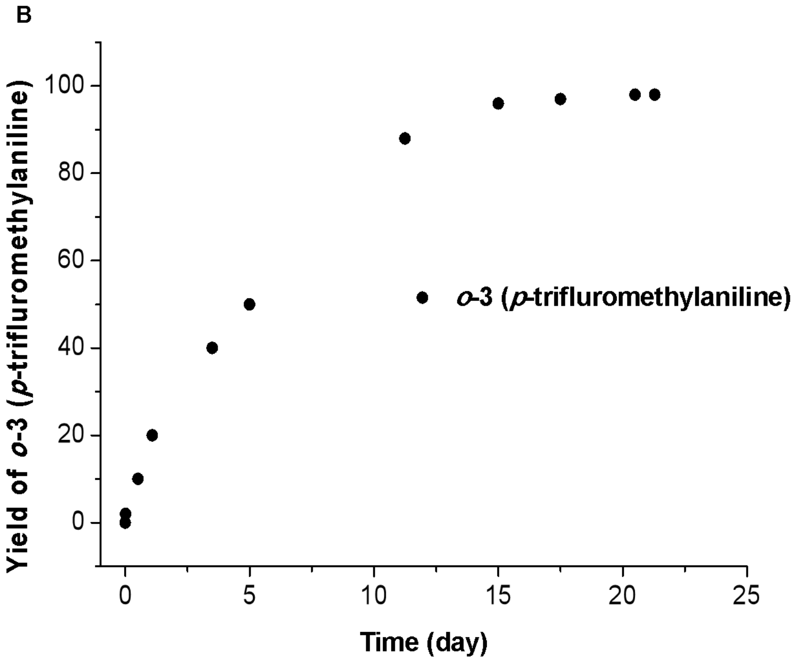

Supplementary Figure 69. (A) Stacked ^{1}H NMR (400 MHz, 20 °C) spectra of the reaction of ***o*-1** (5 mM) with *p*-trifluromethylaniline (1.5 equiv.) in CD_3CN at varied time. (B) The kinetics profile of the reaction of ***o*-1** with *p*-trifluromethylaniline in 22 d.

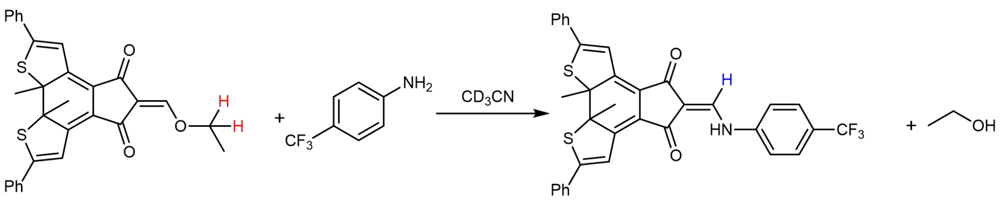

c-1 *c*-3 (*p*-trifluromethylaniline)

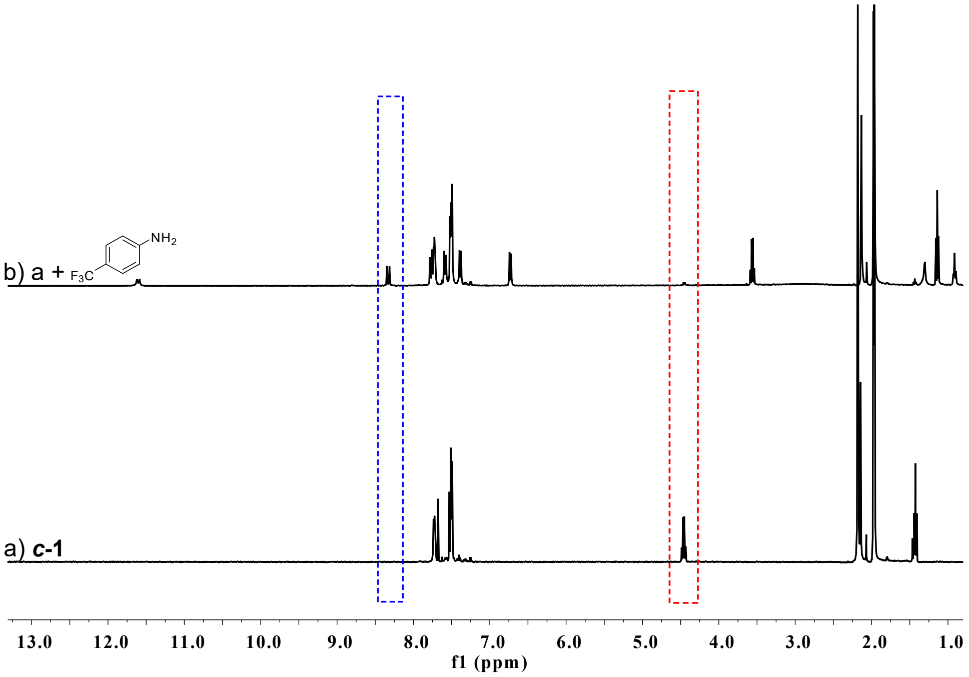

Supplementary Figure 70. (a) ^{1}H NMR (400 MHz, 20 °C) spectrum of ***c*-1** in CD$_3$CN; (b) The addition of *p*-trifluromethylaniline (1.5 equiv.) into the panel a and the reaction after 3 min.

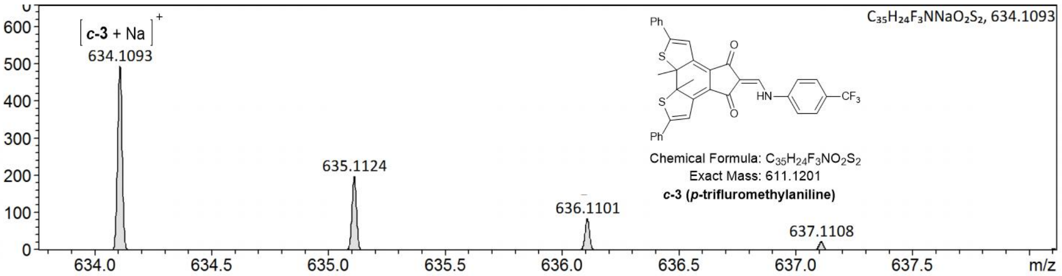

Supplementary Figure 71. ESI mass spectrum of the reaction of ***c*-1** and *p*-trifluromethylaniline in acetonitrile.

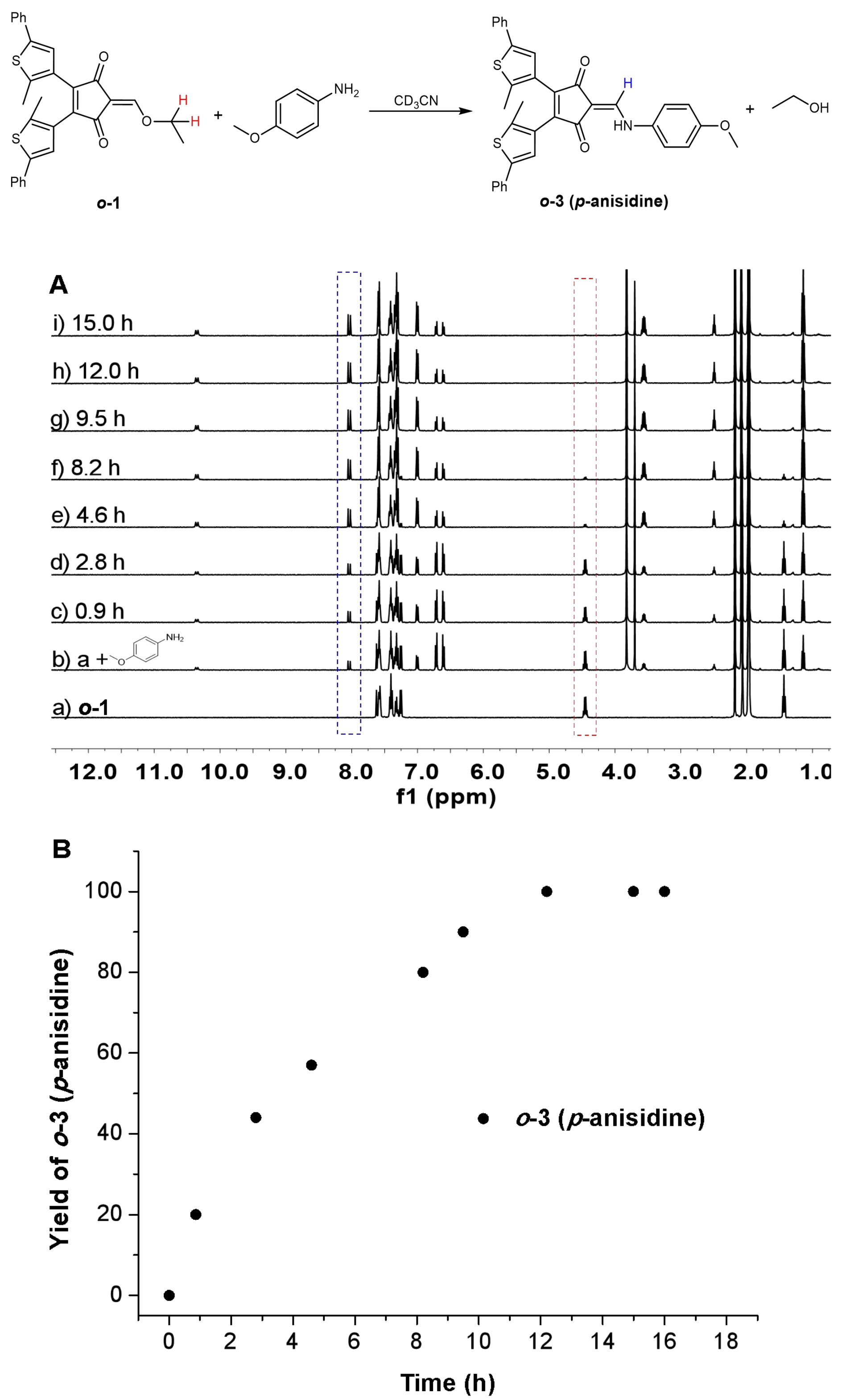

Supplementary Figure 72. (A) Stacked ^{1}H NMR (400 MHz, 20 °C) spectra of the reaction of ***o*-1** (5 mM) with *p*-anisidine (1.5 equiv.) in CD_3CN at varied time. (B) The kinetics profile of the reaction of ***o*-1** with *p*-anisidine in 17 h.

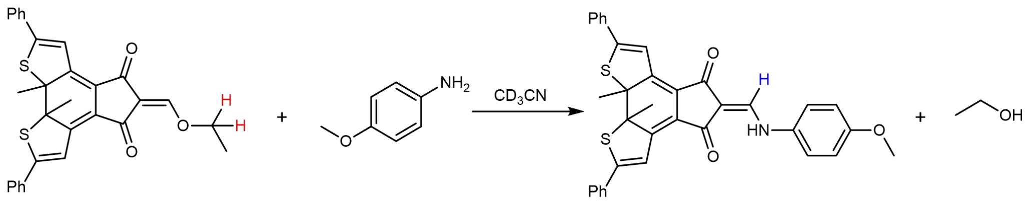

c-1 *c*-3 (*p*-anisidine)

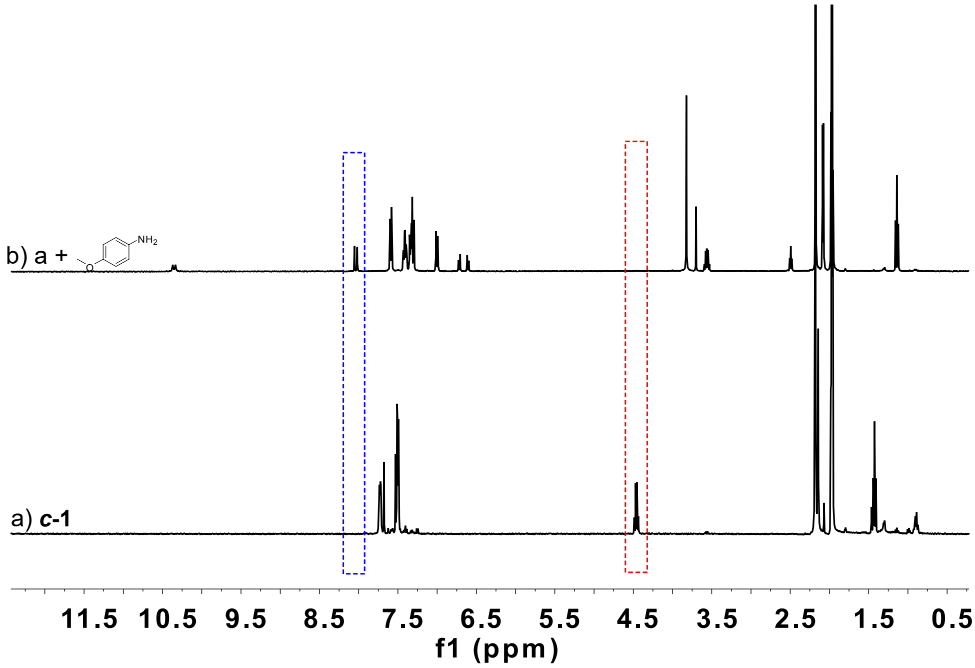

Supplementary Figure 73. (a) ^{1}H NMR (400 MHz, 20 °C) spectrum of ***c*-1** in CD_3CN; (b) The addition of *p*-anisidine (1.5 equiv.) into the panel a and the reaction after 3 min.

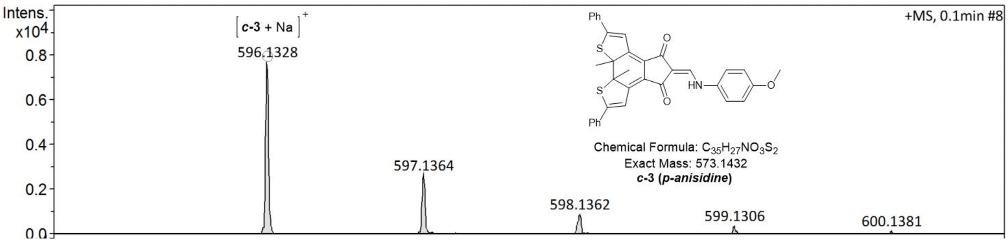

Supplementary Figure 74. ESI mass spectrum of the reaction of ***c*-1** and *p*-anisidine in acetonitrile.

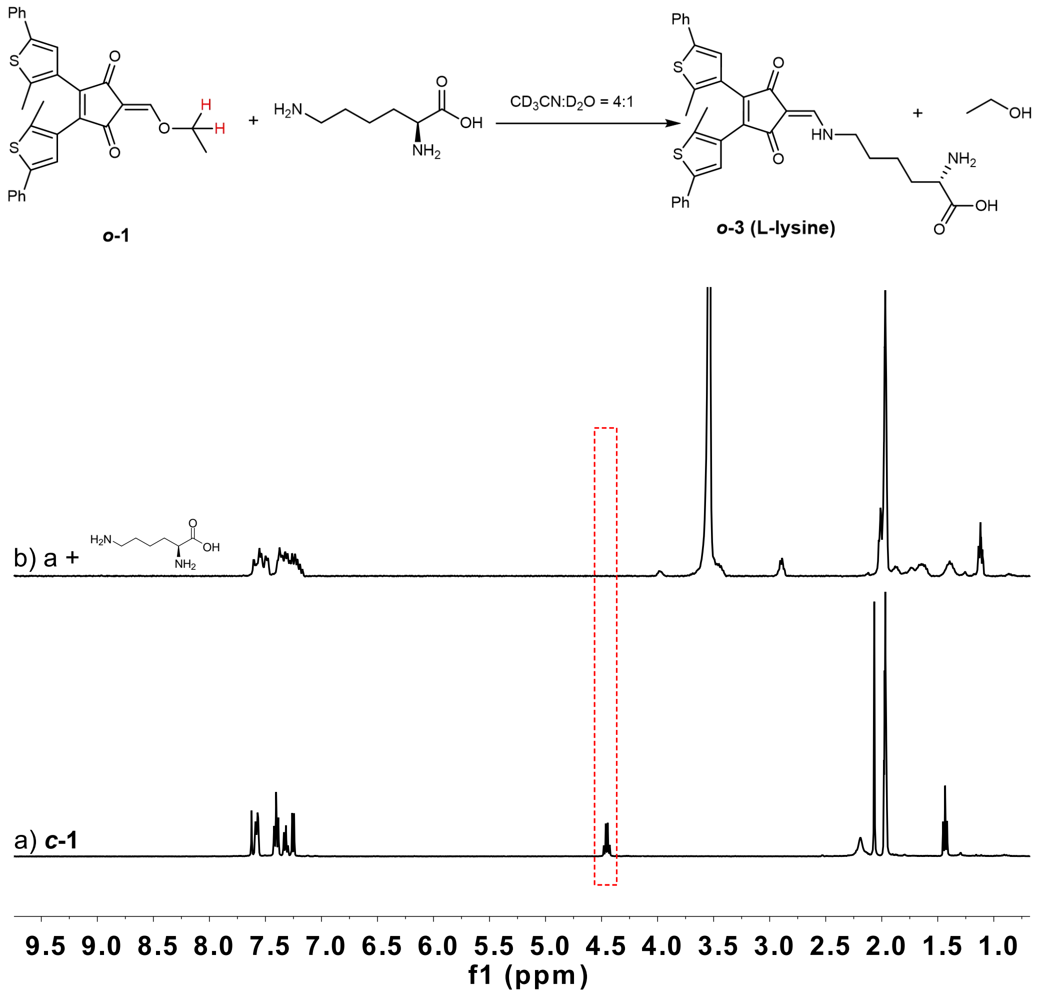

Supplementary Figure 75. (a) ^{1}H NMR (400 MHz, 20 °C) spectrum of ***o*-1** (5 mM) in CD_3CN:D_2O = 4:1 (0.5 mL). (b) After addition of L-lysine (3 equiv., 0.125 mL in D_2O) into the panel a and the reaction after 3 min.

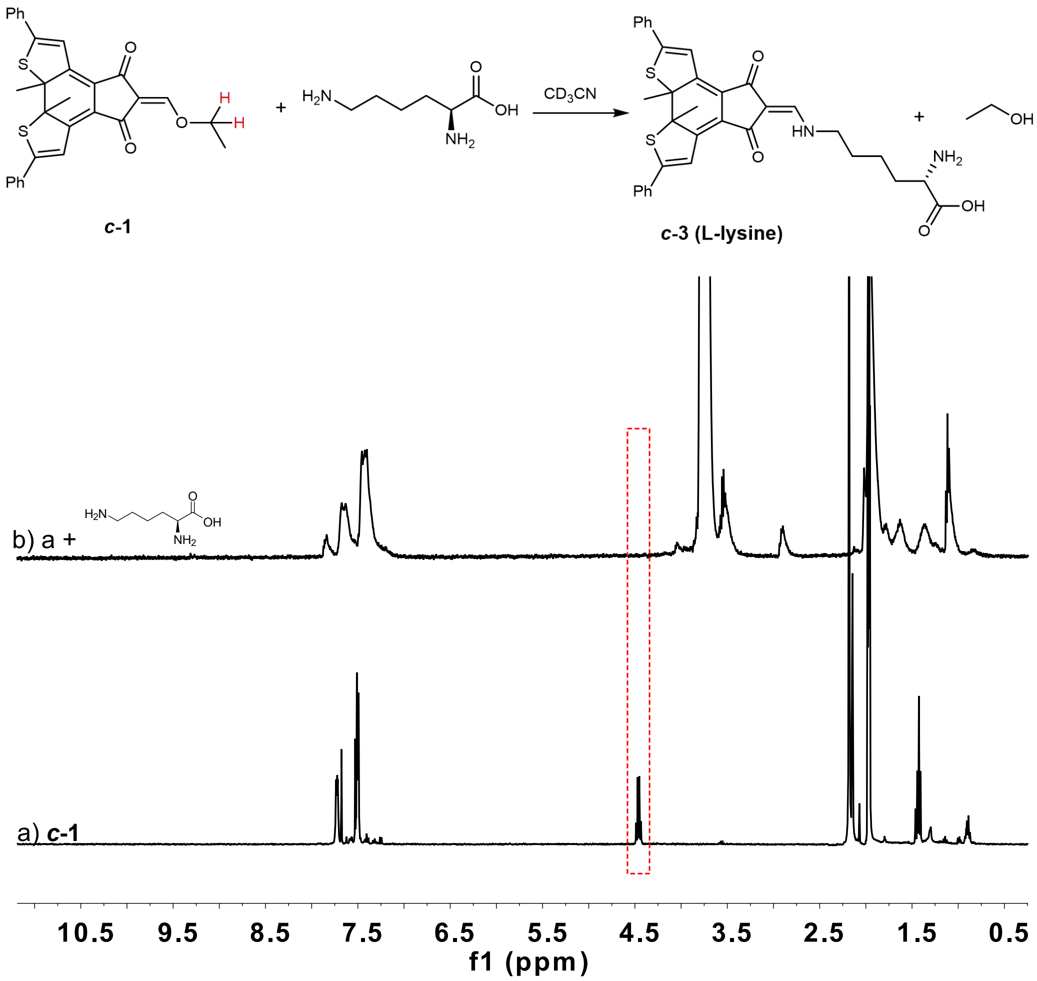

Supplementary Figure 76. (a) ^{1}H NMR (400 MHz, 20 °C) spectrum of ***c*-1** (5 mM) in CD_3CN (0.5 mL). (b) After addition of L-lysine (3 equiv., 0.125 mL in D_2O) into the panel a and the reaction after 3 min.

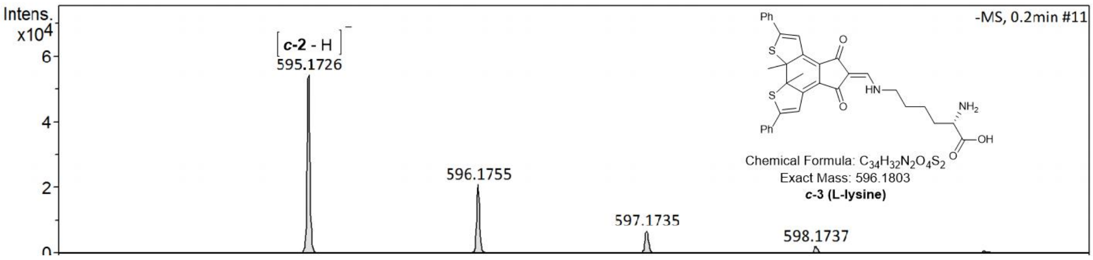

Supplementary Figure 77. ESI mass spectrum of the reaction of ***c*-1** and L-lysine in 4:1 MeCN:H_2O.

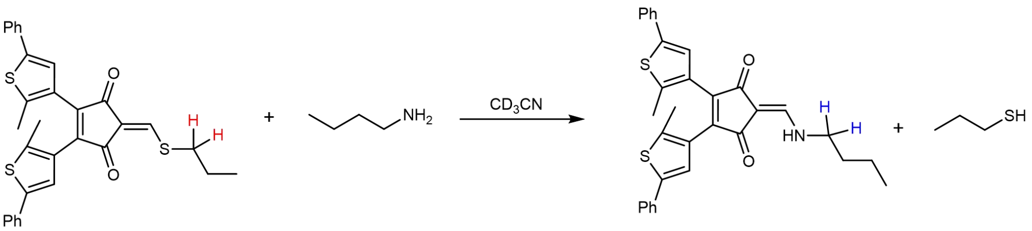

***o*-2 (1-propanethiol)** ***o*-3 (1-butylamine)**

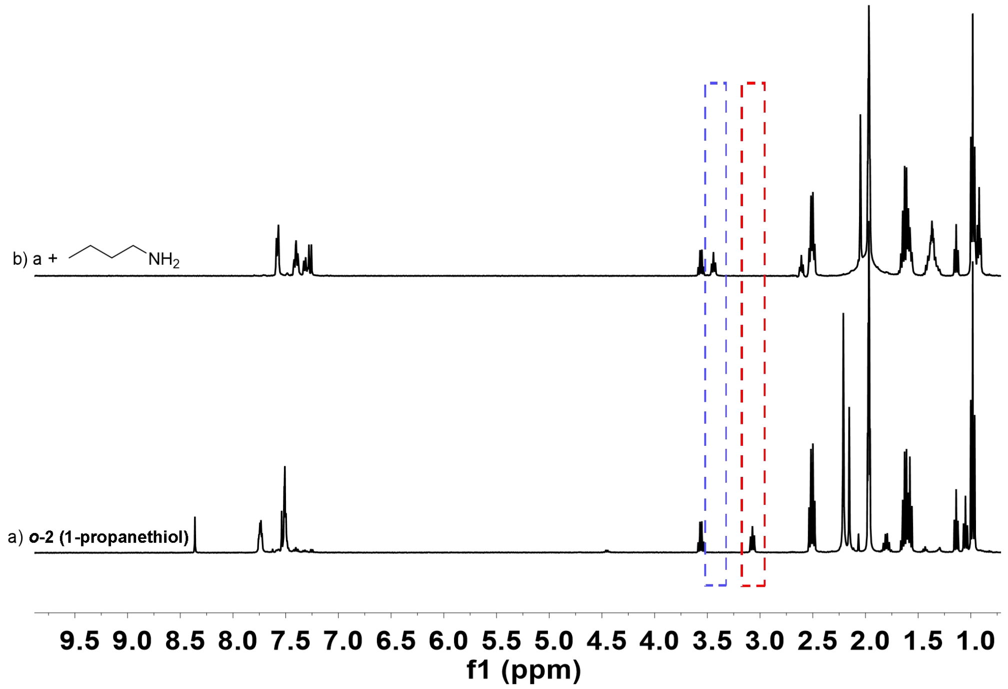

Supplementary Figure 78. (a) ^{1}HNMR (400 MHz, 20 °C) spectrum of ***o*-2** created from **1** (5 mM) and 1-propanethiol (3.0 equiv.) *in situ* in CD_3CN; (b) The addition of 1-butylamine (1.5 equiv.) into the panel a. The reaction was complete within 3 min to afford ***o*-3**.

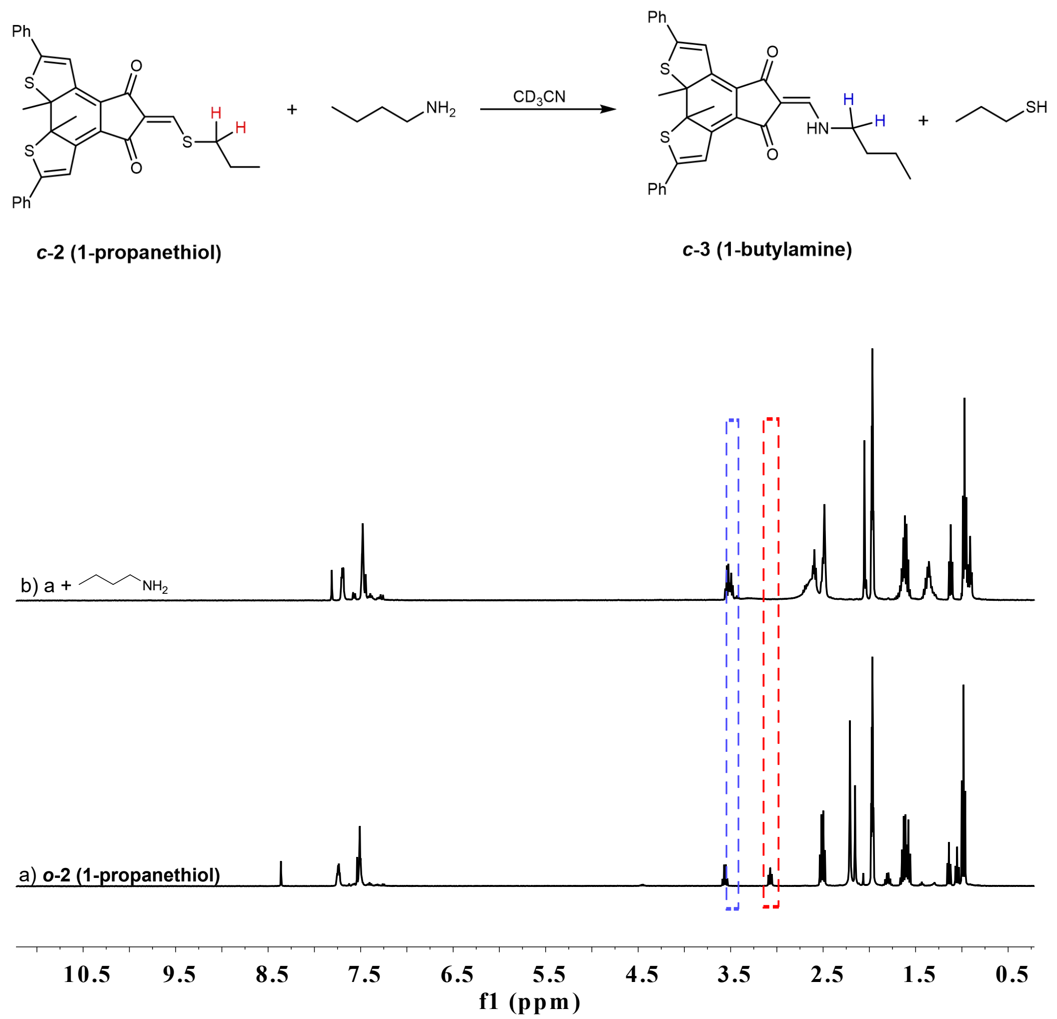

Supplementary Figure 79. (a) ^{1}H NMR (400 MHz, 20 °C) spectrum of ***c*-2** created from **1** (5 mM) and 1-propanethiol (3.0 equiv.) *in situ* in CD_3CN; (b) The addition of 1-butylamine (1.5 equiv.) into the panel a. The reaction was complete within 3 min to afford ***c*-3**.

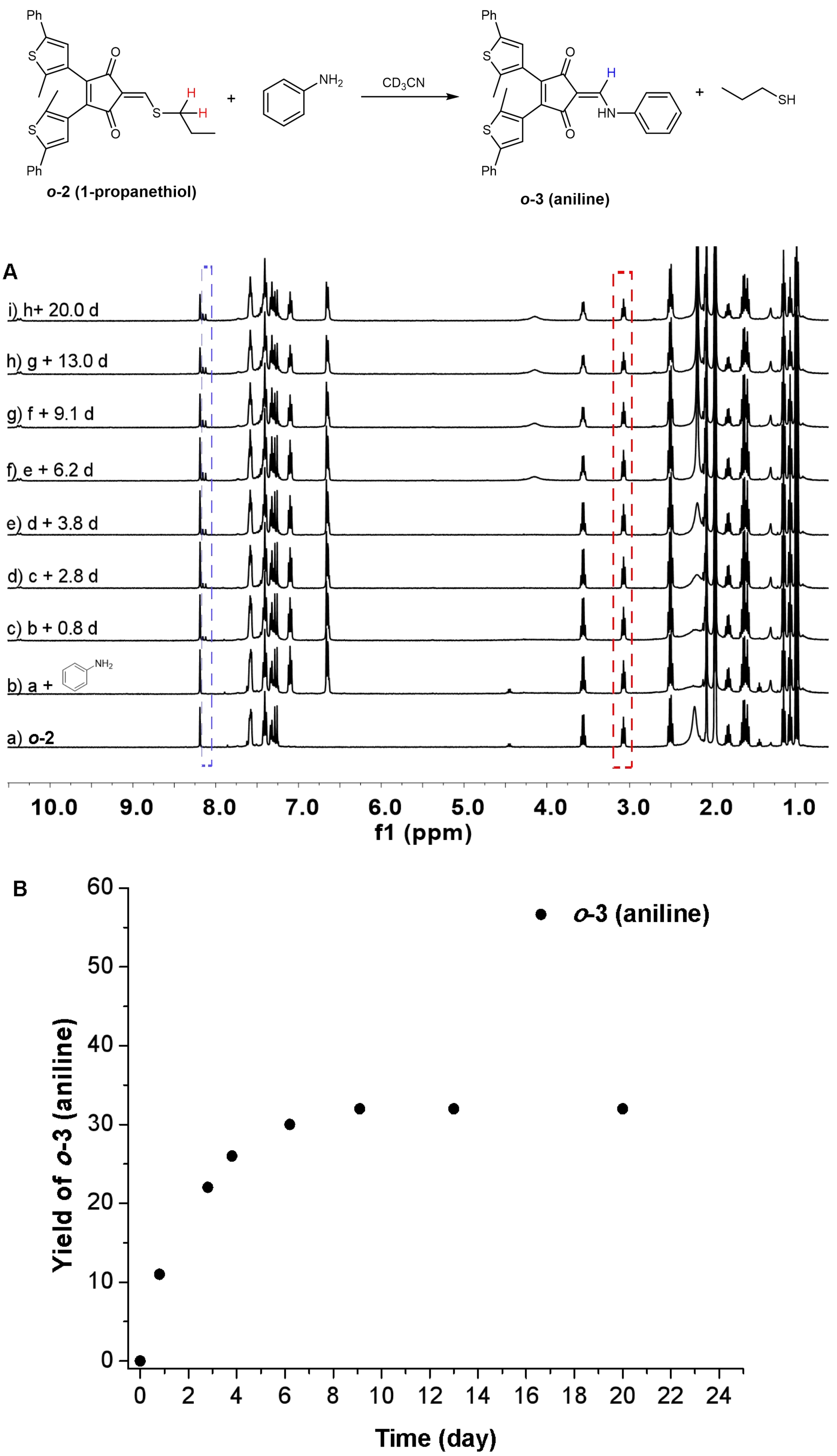

Supplementary Figure 80. (A) Stacked ^{1}H NMR (400 MHz, 20 °C) spectra of the reaction of ***o*-2** created from **1** (5 mM) and 1-propanethiol (3.0 equiv.) *in situ* with aniline (1.5 equiv.) in CD_3CN at varied time. (B) The kinetics profile of the reaction of ***o*-2** with aniline in 20 d.

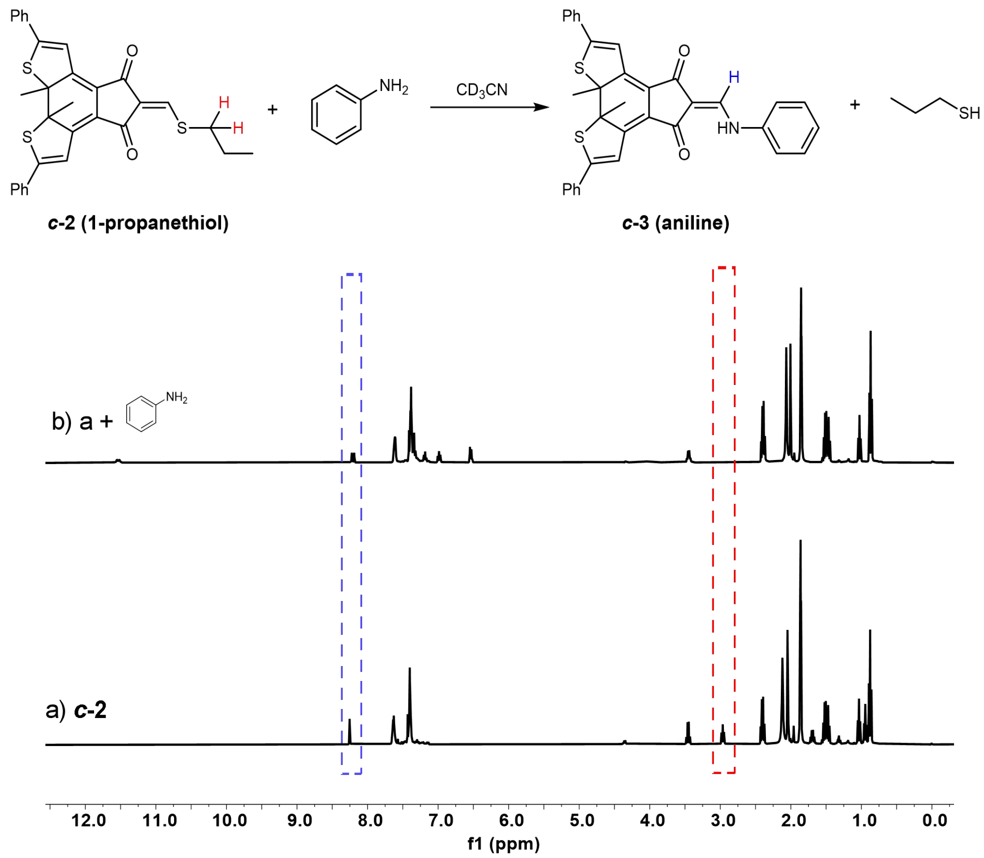

Supplementary Figure 81. (a) ^{1}H NMR (400 MHz, 20 °C) spectrum of ***c*-2** created from **1** (5 mM) and 1-propanethiol (3.0 equiv.) *in situ* in CD_3CN; (b) The addition of aniline (1.5 equiv.) into the panel a. The reaction was complete within 3 min to afford ***c*-3**.

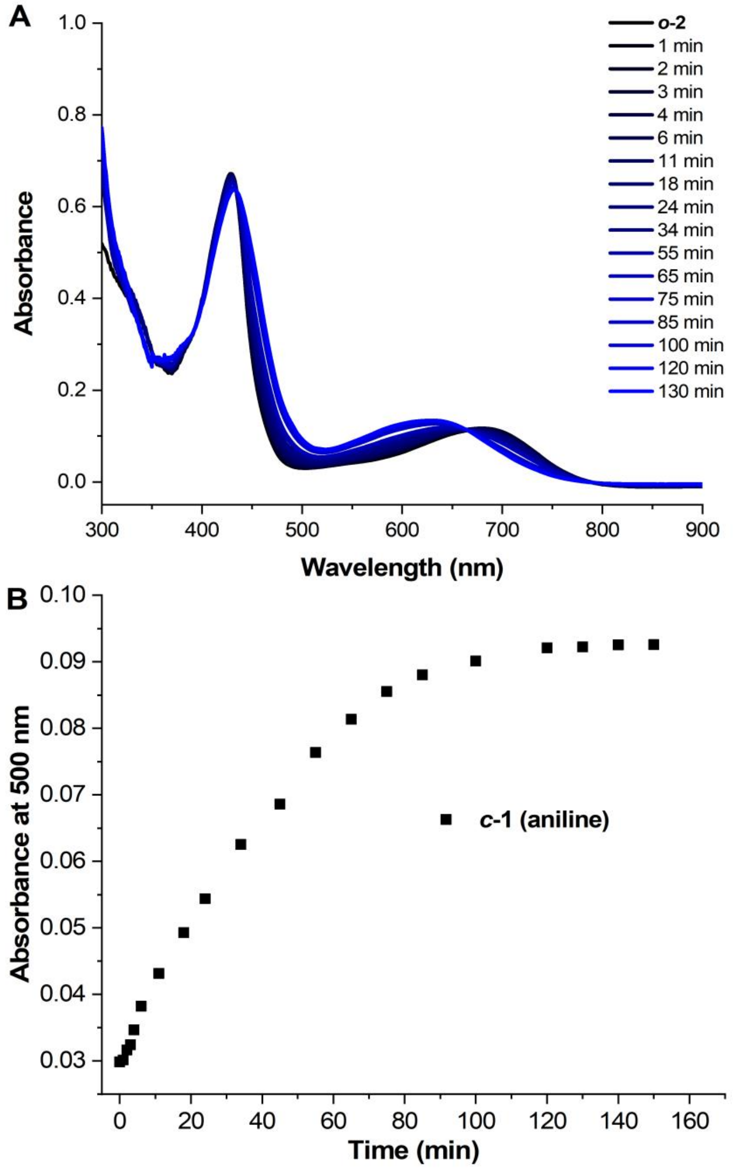

Supplementary Figure 82. (A) Absorbance spectra of the reaction of 1-propanethiol derived ***c*-2** (25 μM, 25 °C) with aniline (1.5 equiv.) in CH_3CN. (B) The kinetics profile of the reaction of ***c*-2** with aniline in 150 min.

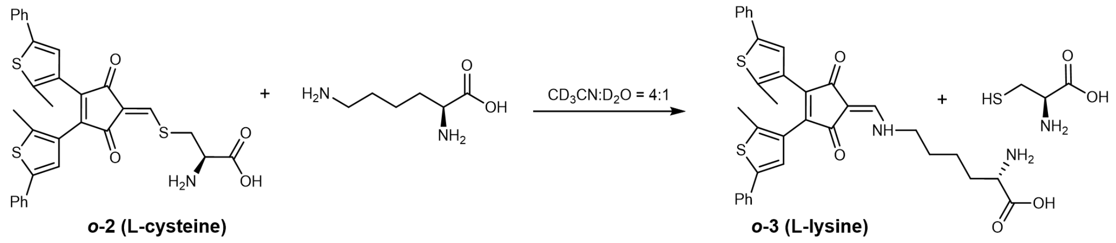

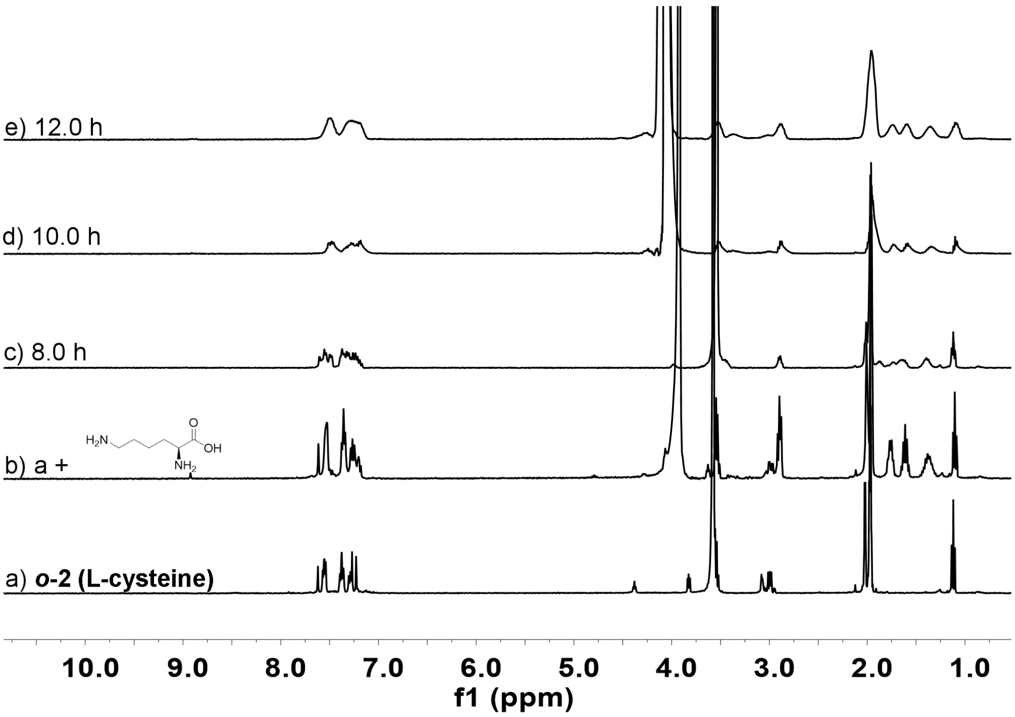

Supplementary Figure 83. (a) ^{1}H NMR (400 MHz, 20 °C) spectrum of ***o*-2** created from **1** (5 mM) and L-cysteine (5 mM) in 4:1 $CD_3CN:D_2O$ (0.5 mL); (b) After addition of L-lysine (3 equiv.) into the panel a; (c-e) The reaction of ***o*-2** with L-lysine (3 equiv.) in 4:1 $CD_3CN:D_2O$ (0.5 mL) at varied time. The reaction was complete within 12 h.

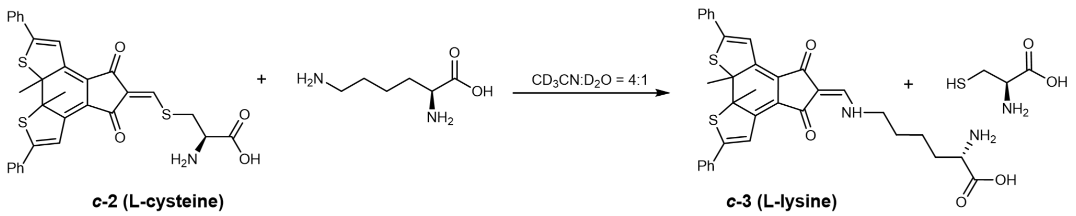

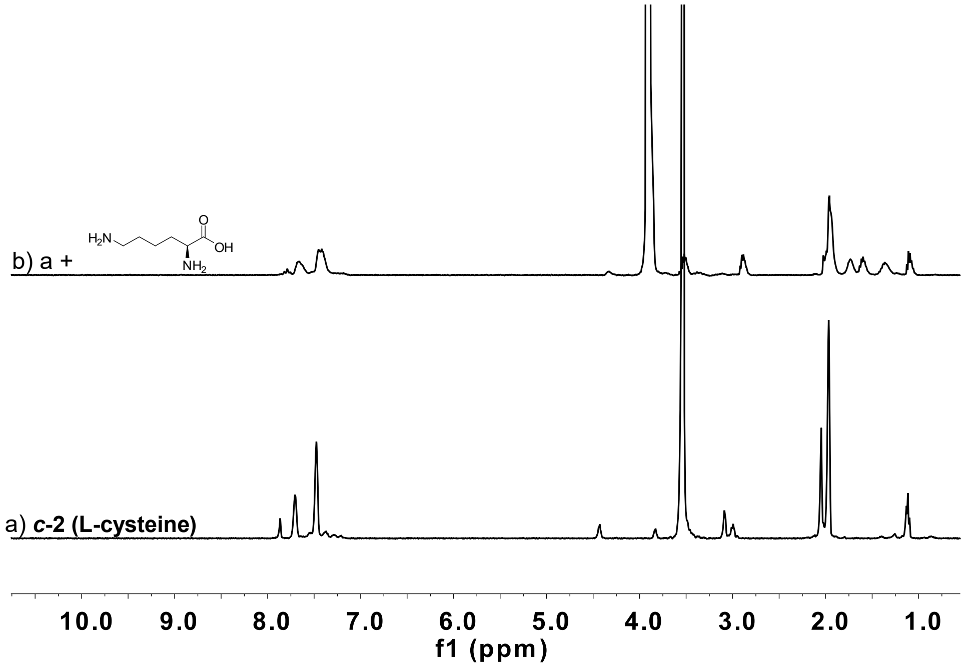

Supplementary Figure 84. (a) ^{1}H NMR (400 MHz, 20 °C) spectrum of ***c*-2** created from **1** (5 mM) and L-cysteine (5 mM) in 4:1 CD_3CN:D_2O (0.5 mL). (b) After addition of L-lysine (3 equiv.) into the panel a. The reaction was complete within 3 min to afford ***c*-3**.

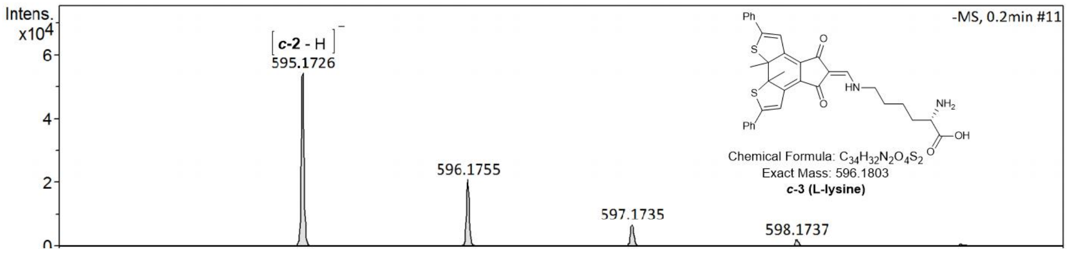

Supplementary Figure 85. ESI mass spectrum of the reaction of L-cysteine derived ***c*-2** and L-lysine in 4:1 MeCN:H_2O.

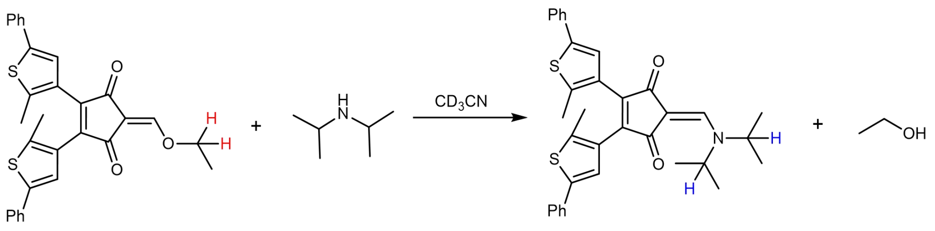

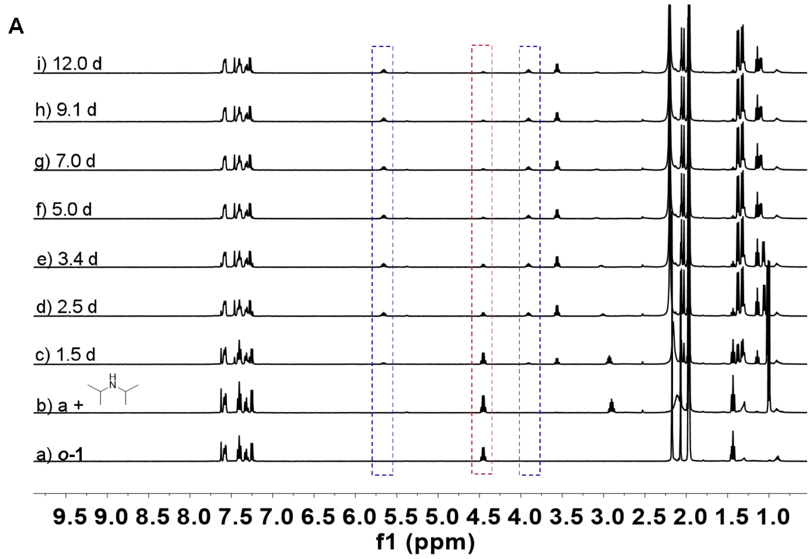

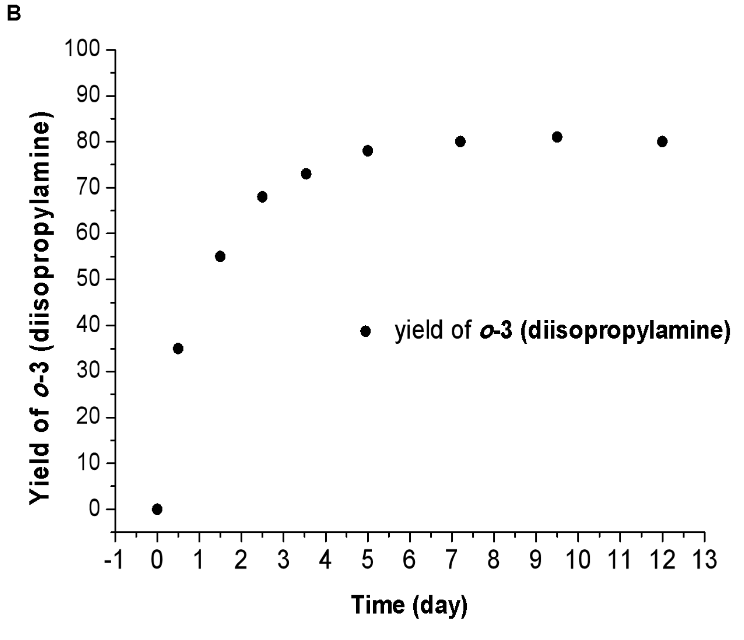

Supplementary Figure 86. (A) Stacked ^{1}H NMR (400 MHz, 20 °C) spectra of the reaction of ***o*-1** (5 mM) with diisopropylamine (1.5 equiv.) in CD_3CN at varied time. (B) The kinetics profile of the reaction of ***o*-1** with diisopropylamine in 12 d.

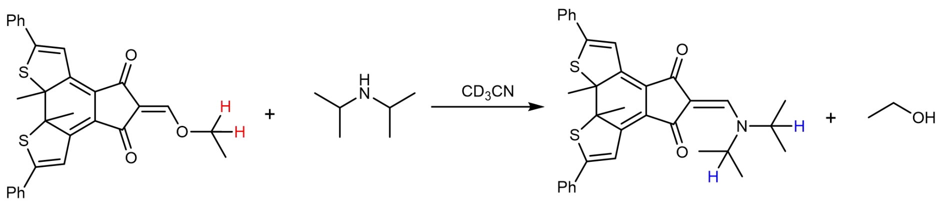

c-1

c-3 (diisopropylamine)

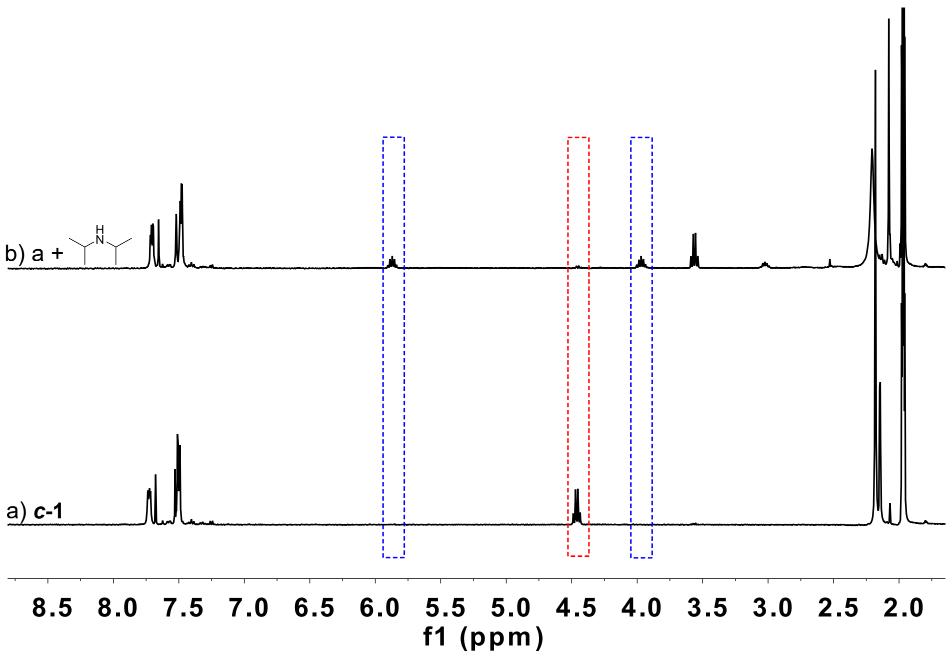

Supplementary Figure 87. (a) ^{1}H NMR (400 MHz, 20 °C) spectrum of ***c*-1** in CD_3CN; (b) The addition of diisopropylamine (1.5 equiv.) into the panel a and the reaction after 3 min.

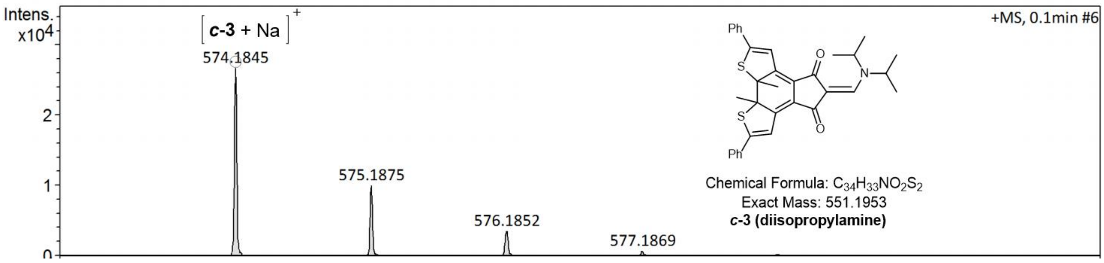

Supplementary Figure 88. ESI mass spectrum of the reaction of ***c*-1** and diisopropylamine in acetonitrile.

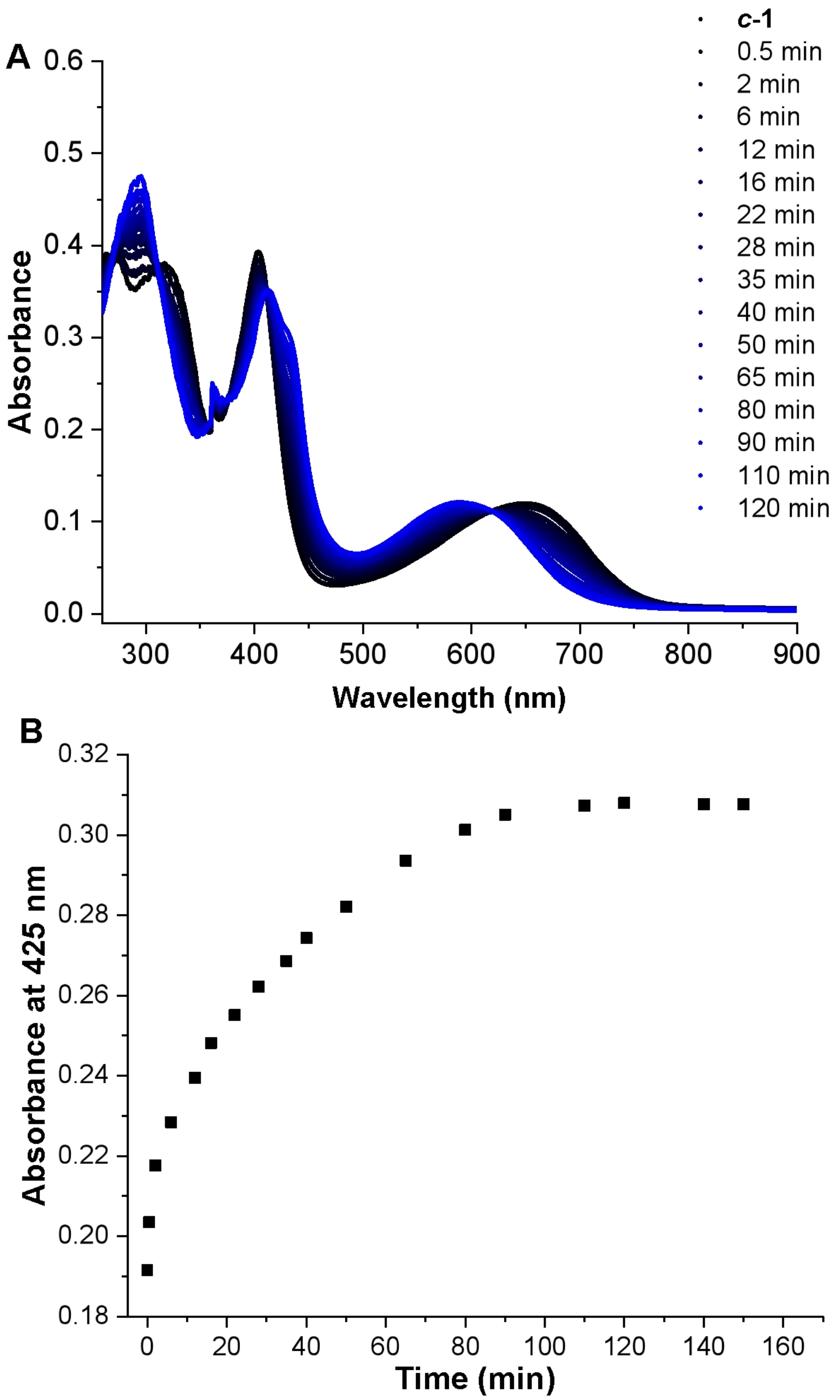

Supplementary Figure 89. (A) Absorbance spectra of the reaction of ***c*-1** (25 μM, 25 °C) with diisopropylamine (1.5 equiv.) in CH_3CN. (B) The kinetics profile of the reaction of ***c*-1** with diisopropylamine in 150 min.

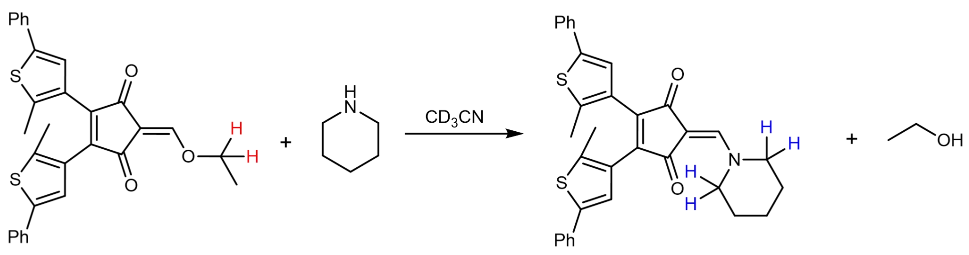

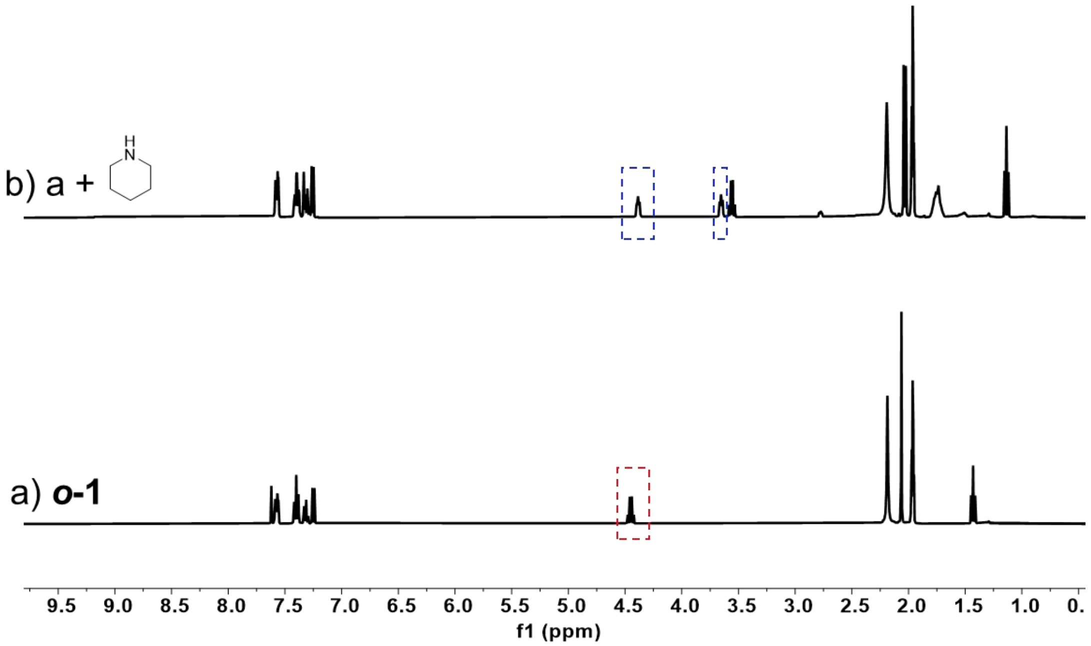

Supplementary Figure 90. (a) ^{1}H NMR (400 MHz, 20 °C) spectrum of ***o*-1** in CD_3CN; (b) The addition of piperidine (1.5 equiv.) into the panel a and the reaction after 3 min.

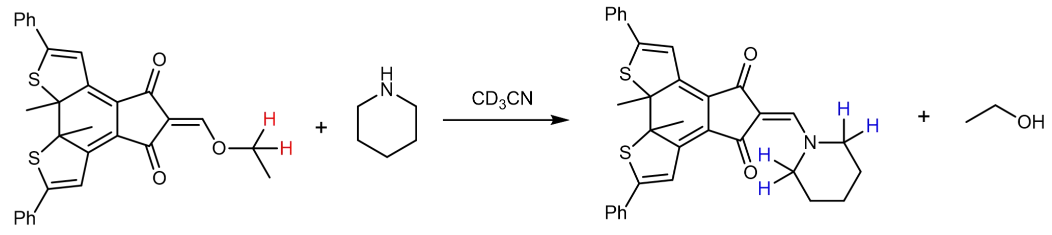

***c*-1** ***c*-3 (piperidine)**

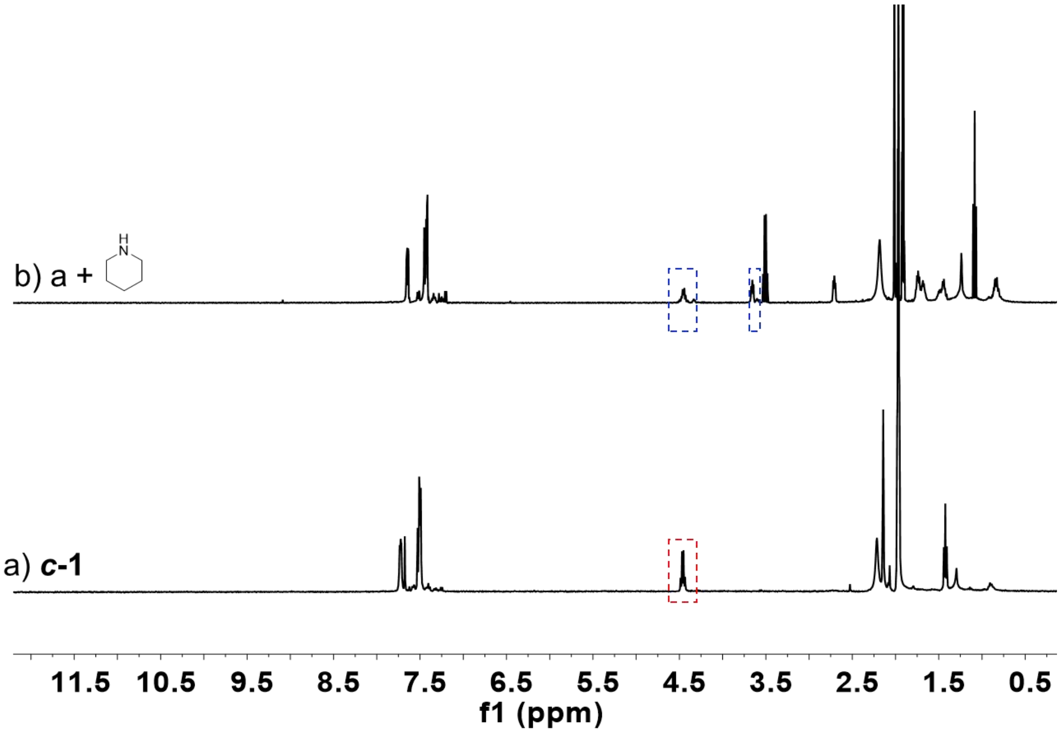

Supplementary Figure 91. (a) ^{1}H NMR (400 MHz, 20 ℃) spectrum of ***c*-1** in CD_3CN; (b) The addition of piperidine (1.5 equiv.) into the panel a and the reaction after 3 min.

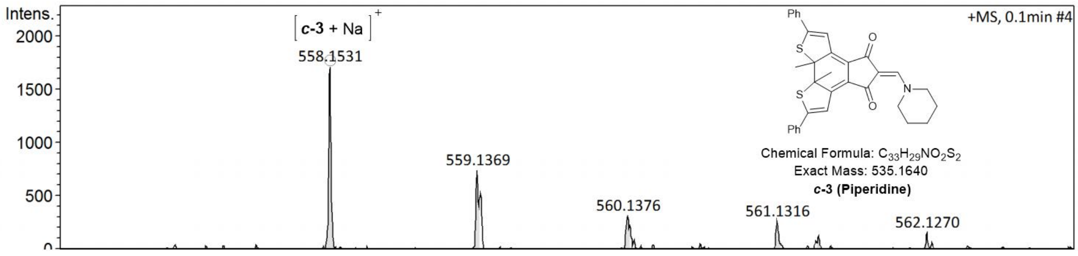

Supplementary Figure 92. ESI mass spectrum of the reaction of ***c*-1** and piperidine in acetonitrile.

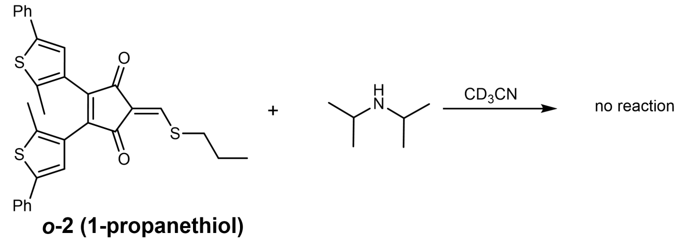

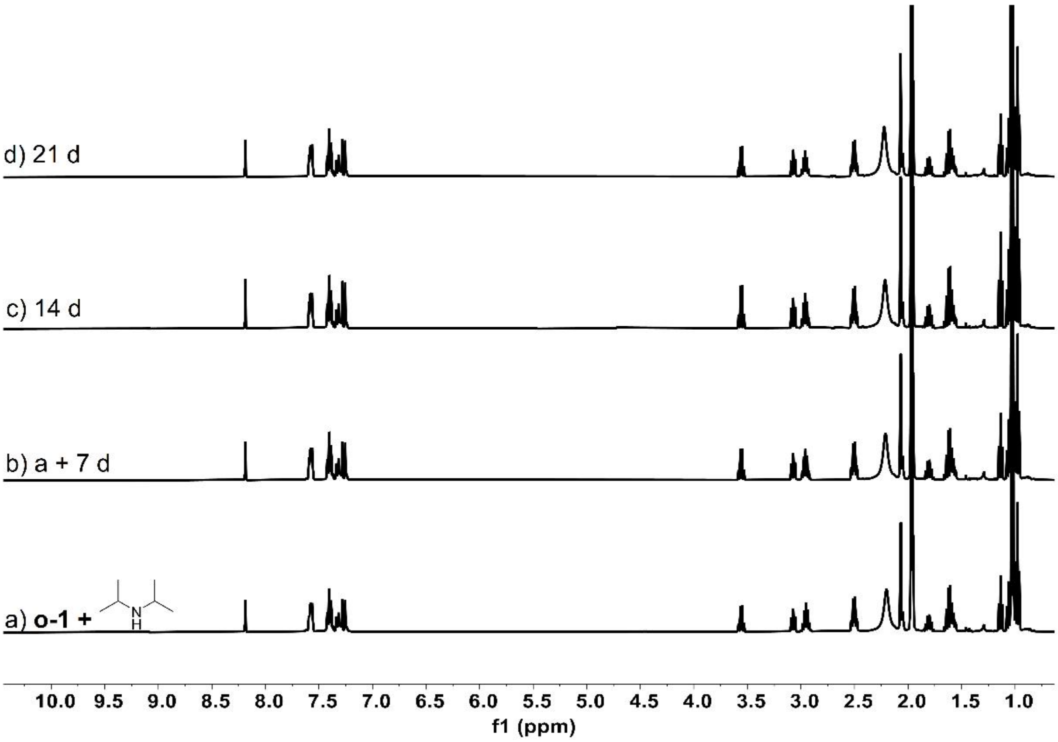

Supplementary Figure 93. Stacked ^{1}H NMR (400 MHz, 20 °C) spectra of the reaction of ***o*-2** (5 mM) with diisopropylamine (1.5 equiv.) in CD_3CN at varied time. No reaction occurred.

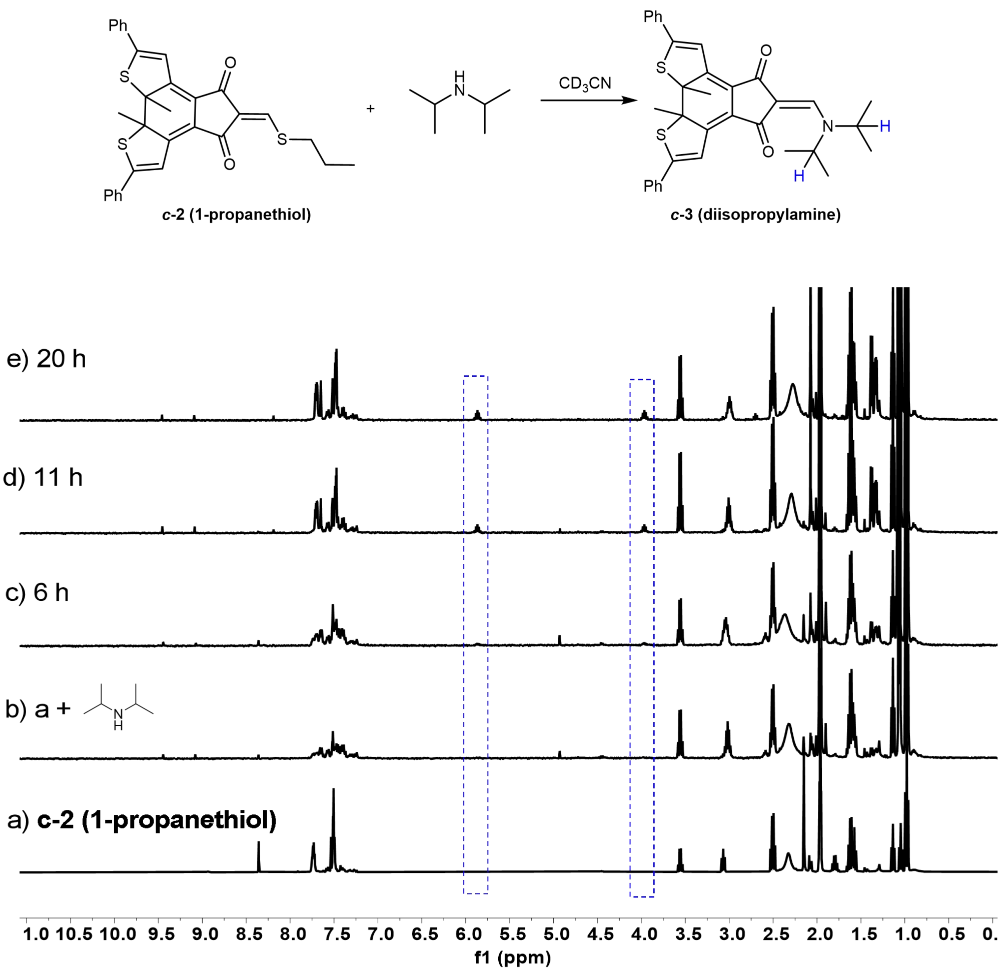

Supplementary Figure 94. Stacked ^{1}H NMR (400 MHz, 20 °C) spectra of the reaction of ***c*-2** (5 mM) with diisopropylamine (1.5 equiv.) in CD_3CN at varied time.

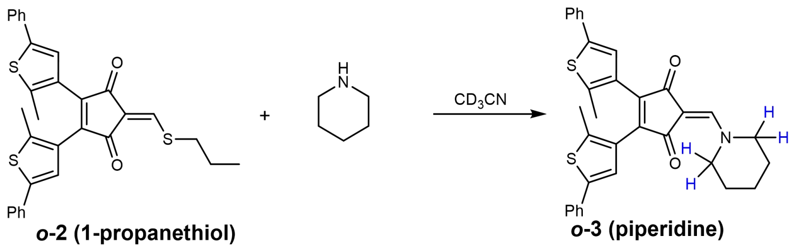

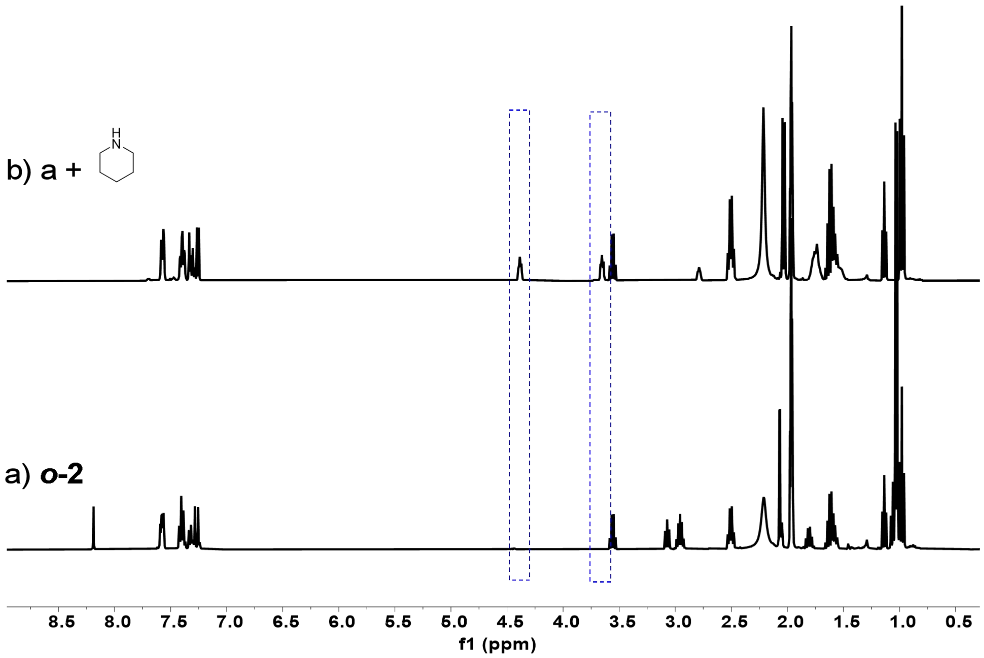

Supplementary Figure 95. ^{1}H NMR (400 MHz, 20 °C) spectrum of the reaction of ***o*-2** (5 mM) with piperidine (1.5 equiv.) in CD_3CN after 3 min.

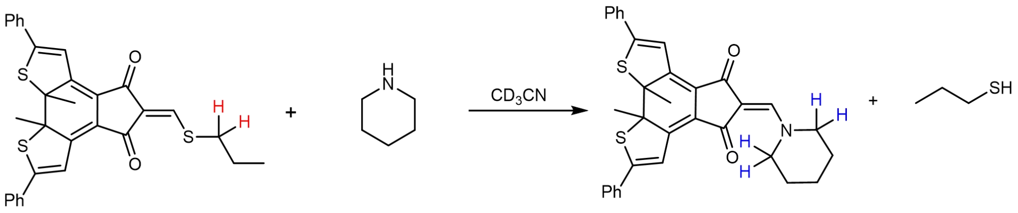

c-2 (1-propanthiol) **c-3 (piperidine)**

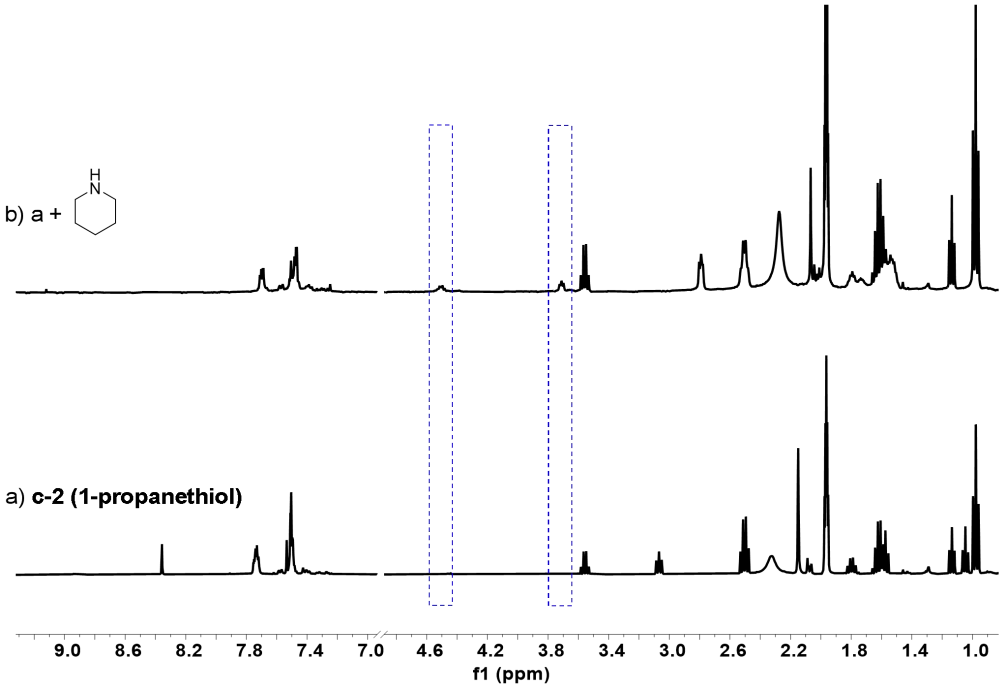

Supplementary Figure 96. ^{1}H NMR (400 MHz, 20 °C) spectrum of the reaction of ***c*-2** (5 mM) with piperidine (1.5 equiv.) in CD_3CN after 3 min.

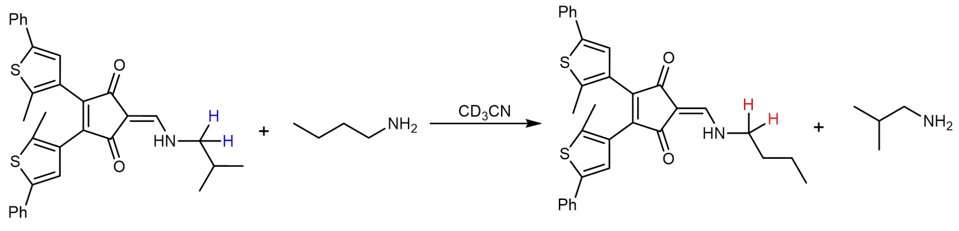

***o*-3 (isobutylamine)** | ***o*-3 (1-butylamine)**

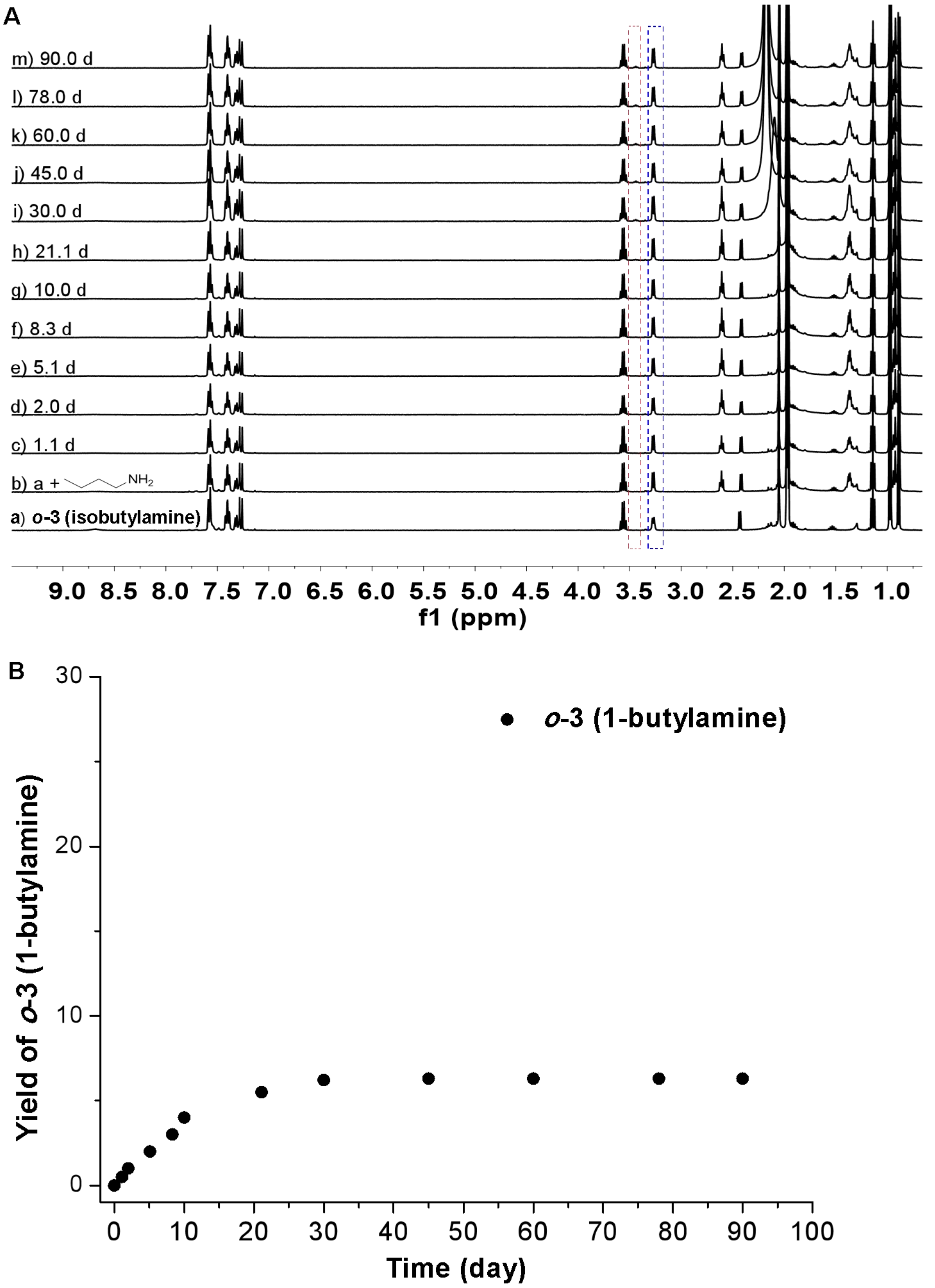

Supplementary Figure 97. (A) Stacked ^{1}H NMR (400 MHz, 20 °C) spectra of the reaction of isobutylamine derived ***o*-3** (5 mM) with 1-butylamine (1.0 equiv.) in CD_3CN at varied time. (B) The kinetics profile of the reaction of isobutylamine derived ***o*-3** with 1-butylamine in 90 d.

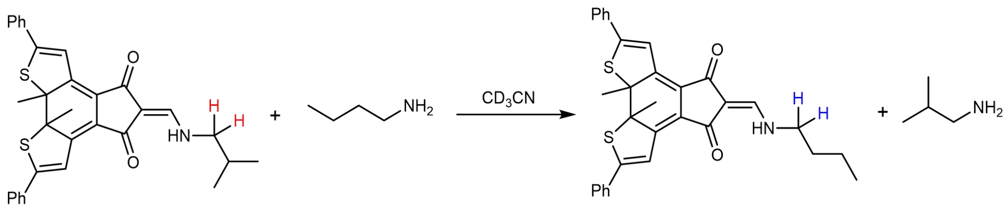

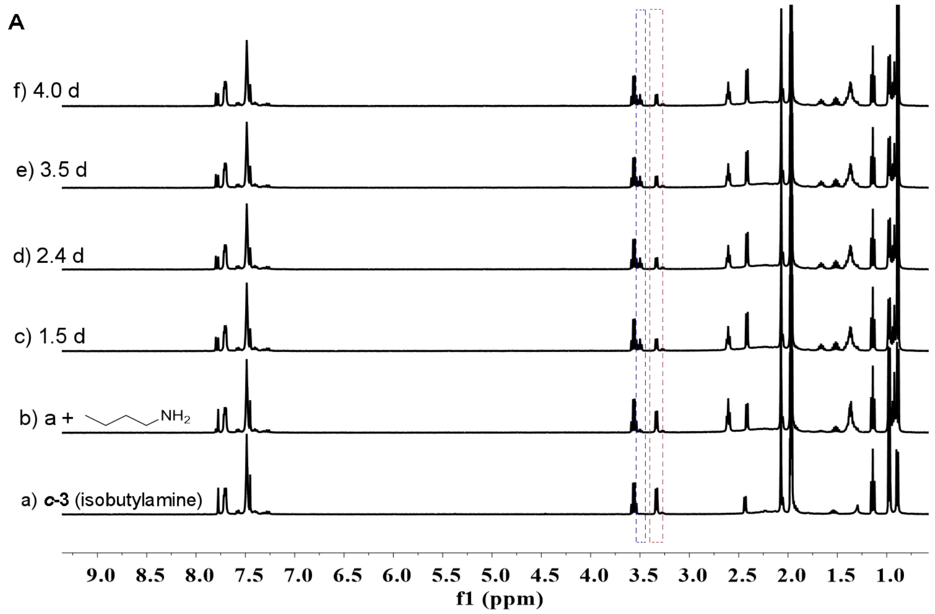

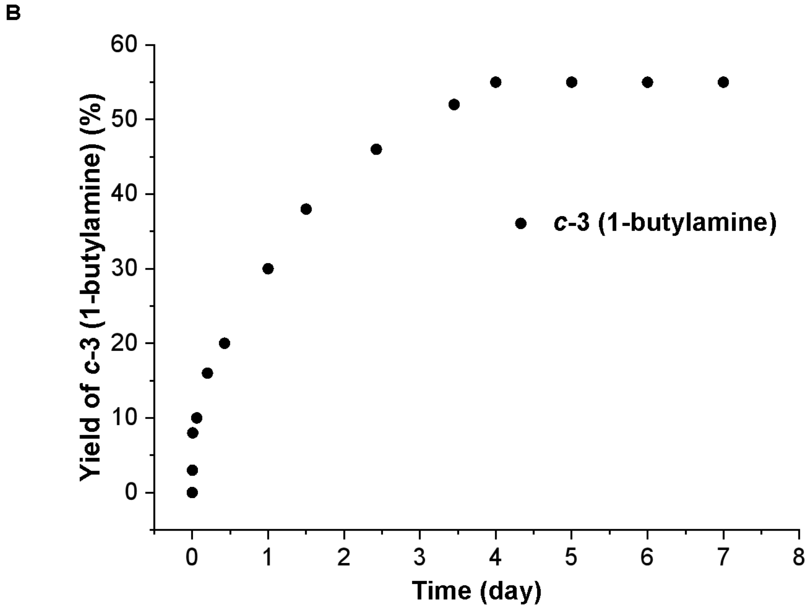

Supplementary Figure 98. (A) Stacked ^{1}H NMR (400 MHz, 20 °C) spectra of the reaction of isobutylamine derived ***c*-3** (5 mM) with 1-butylamine (1.0 equiv.) in CD_3CN at varied time. (B) The kinetics profile of the reaction of isobutylamine derived ***c*-3** with 1-butylamine in 8 d.

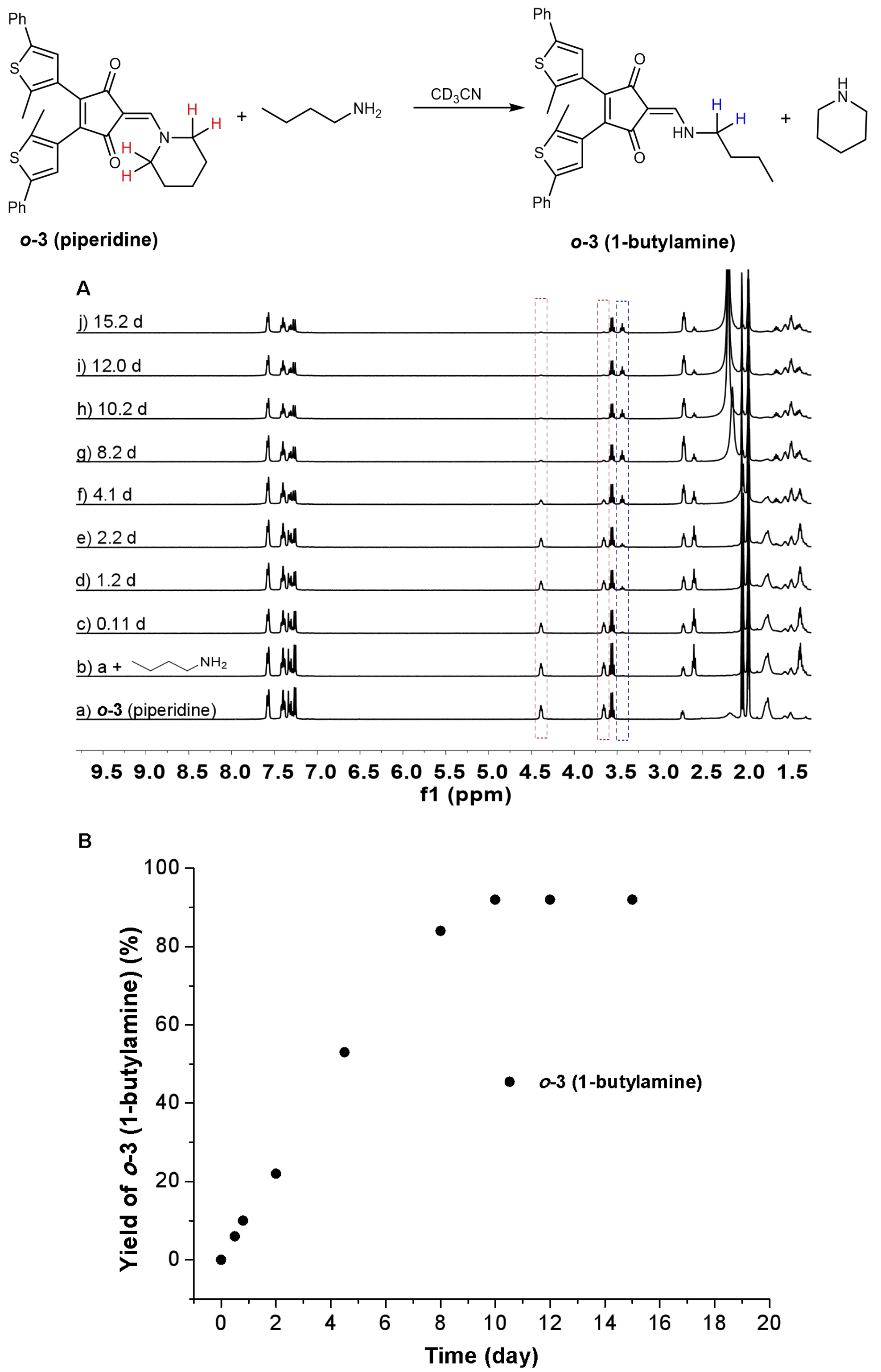

Supplementary Figure 99. (A) Stacked ^{1}H NMR (400 MHz, 20 °C) spectra of the reaction of piperidine derived ***o*-3** (5 mM) with 1-butylamine (1.0 equiv.) in CD_3CN at varied time. (B) The kinetics profile of the reaction of piperidine derived ***o*-3** with 1-butylamine in 17 d.

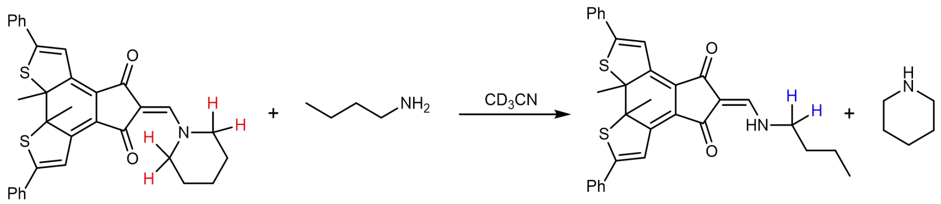

c-3 (piperidine) *c*-3 (1-butylamine)

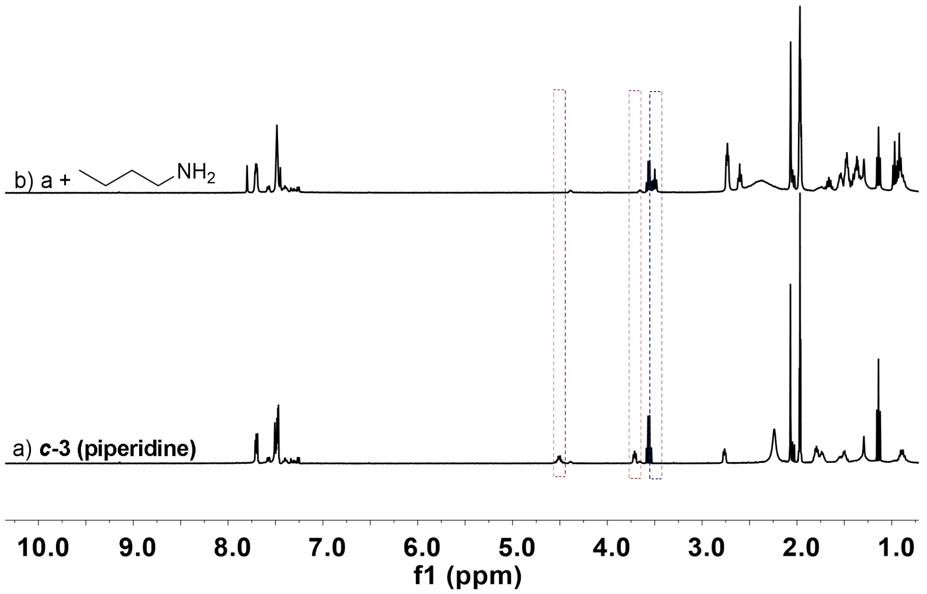

Supplementary Figure 100. (a) ^{1}H NMR (400 MHz, 20 °C) spectrum of piperidine derived ***c*-3** in CD_3CN; (b) The addition of 1-butylamine (1.5 equiv.) into the panel a and the reaction after 3 min.

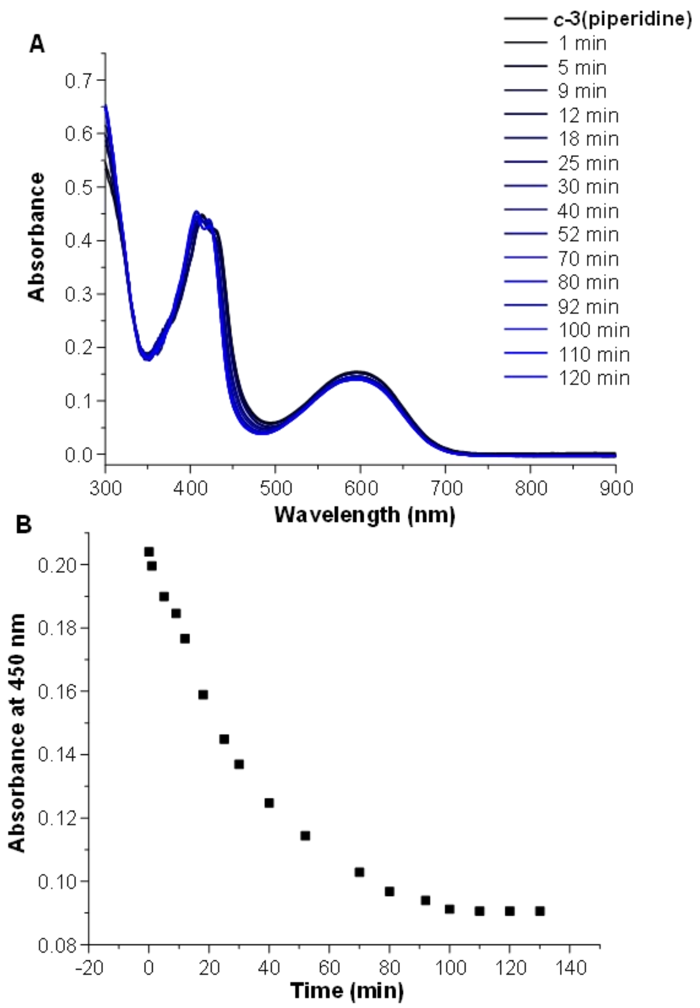

Supplementary Figure 101. (A) Absorbance spectra of the reaction of piperidine derived ***c*-3** (25 μM, 25 °C) with 1-butylamine (1.5 equiv.) in CH_3CN. (B) The kinetics profile of the reaction of ***c*-3** with 1-butylamine in 130 min.

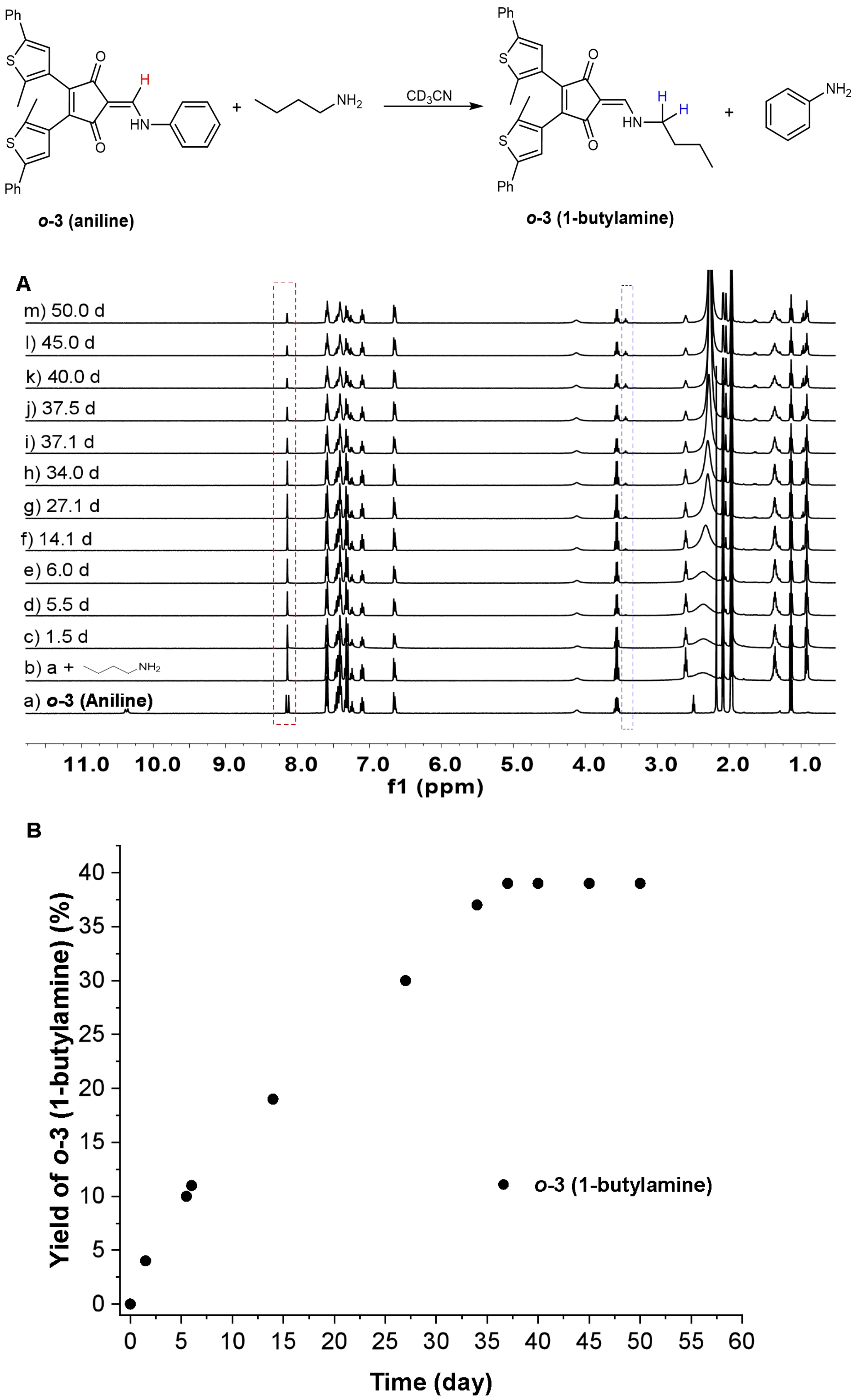

Supplementary Figure 102. (A) Stacked ^{1}H NMR (400 MHz, 20 °C) spectra of the reaction of aniline derived ***o*-3** (5 mM) with 1-butylamine (1.0 equiv.) in CD_3CN at varied time. (B) The kinetics profile of the reaction of aniline derived ***o*-3** with 1-butylamine in 60 d.

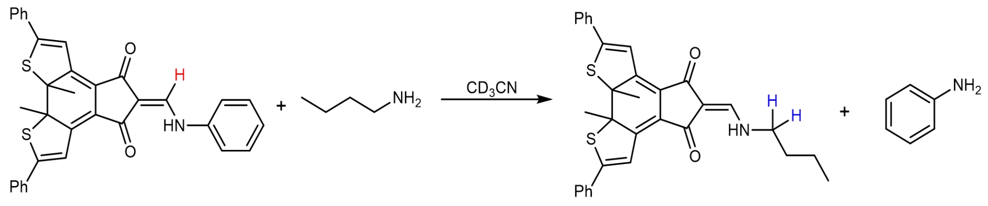

c-3 (aniline) *c*-3 (1-butylamine)

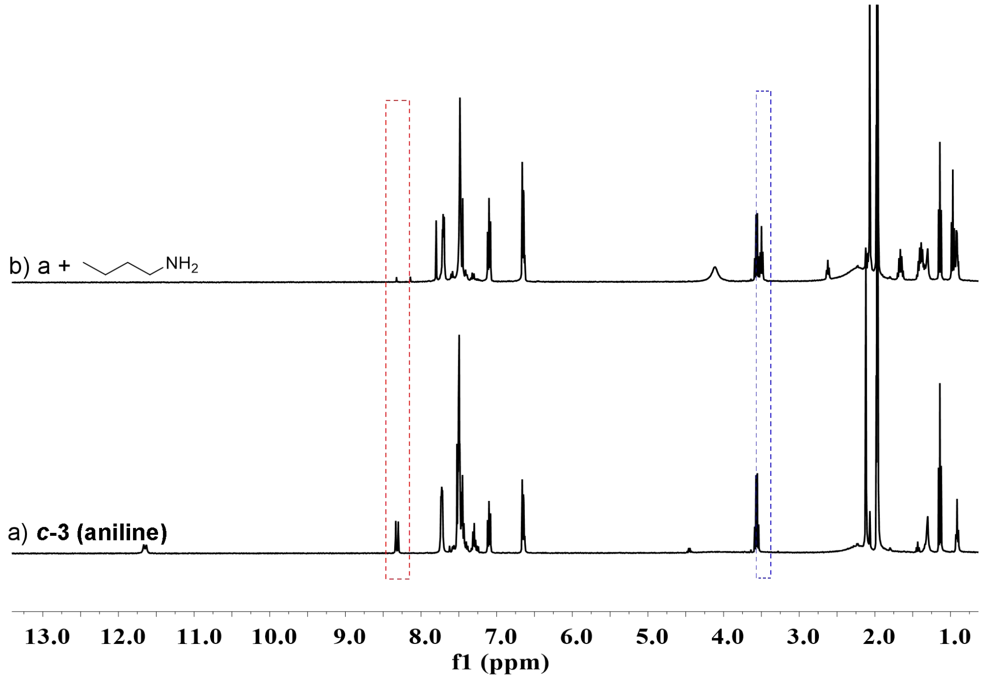

Supplementary Figure 103. (a) ^{1}H NMR (400 MHz, 20 °C) spectrum of aniline derived ***c*-3** in CD_3CN; (b) The addition of 1-butylamine (1.5 equiv.) into the panel a and the reaction after 3 min.

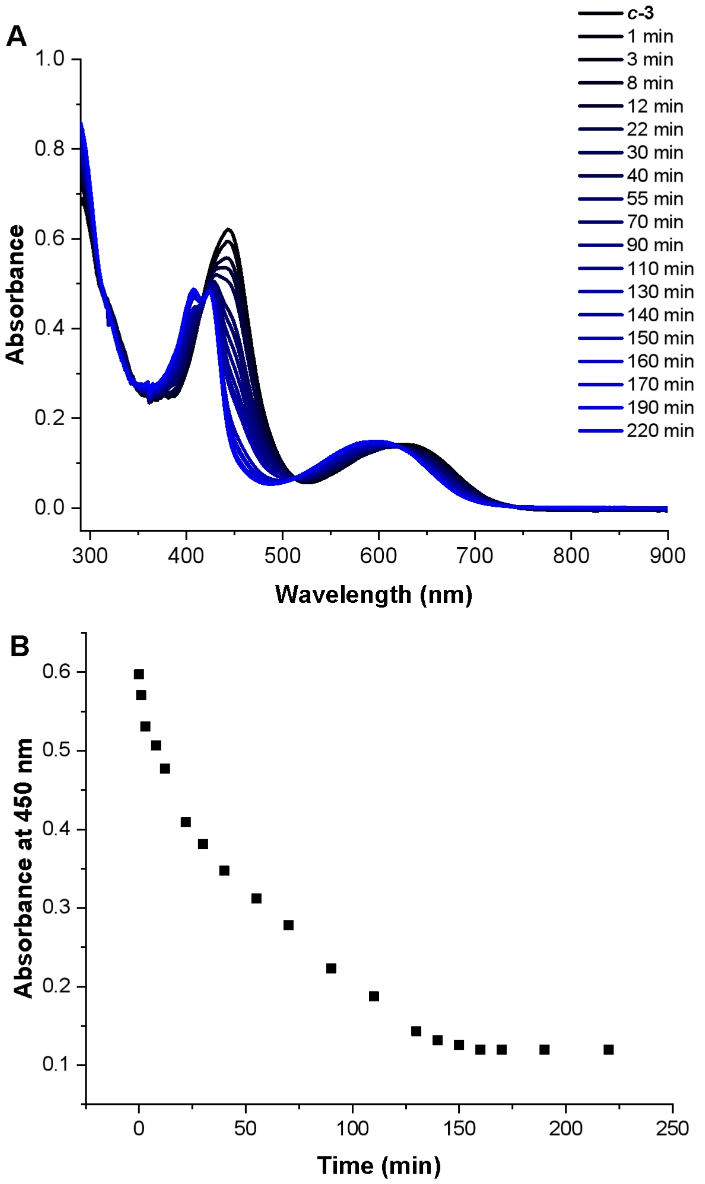

Supplementary Figure 104. (A) Absorbance spectra of the reaction of aniline derived ***c*-3** (25 μM, 25 °C) with 1-butylamine (1.5 equiv.) in CH_3CN. (B) The kinetics profile of the reaction of ***c*-3** with 1-butylamine in 220 min.

6. Determination of the Activation Energy

The pesudo-order rate constants $k_{o,c}$ ($M^{-1}s^{-1}$) at different temperatures were obtained from the exchange experiment of ***o*-1**/***c*-1** with an excess amount of 1-propanthiol. The activation energy can be calculated by plotting ln($k_{o,c}$) versus $\frac{1000}{T}$ (Arrhenius equation).[5]

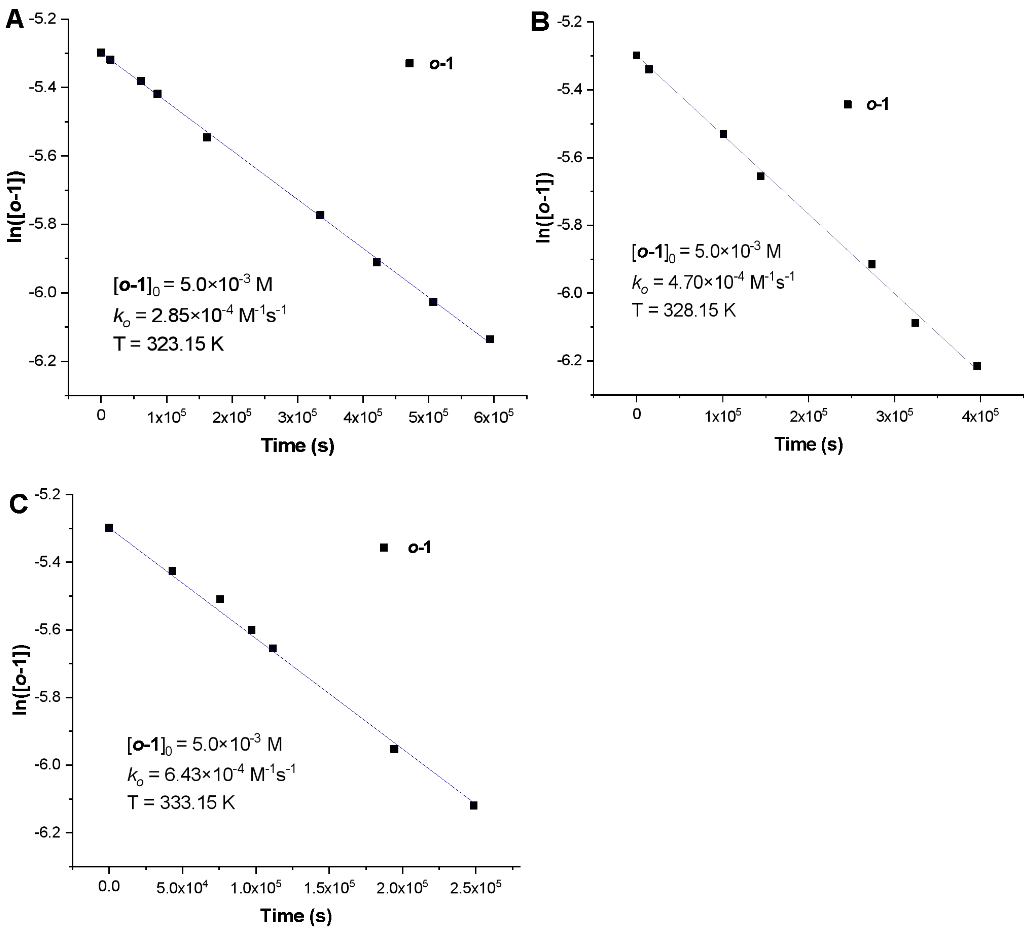

Supplementary Figure 105. Kinetic fitting curves of the reactions of ***o*-1** with 1-propanethiol (10 equiv.) at 328.15 K (A), 323.15 K (B), and 333.15 K (C). The reaction rate was shown in the insert of the figure.

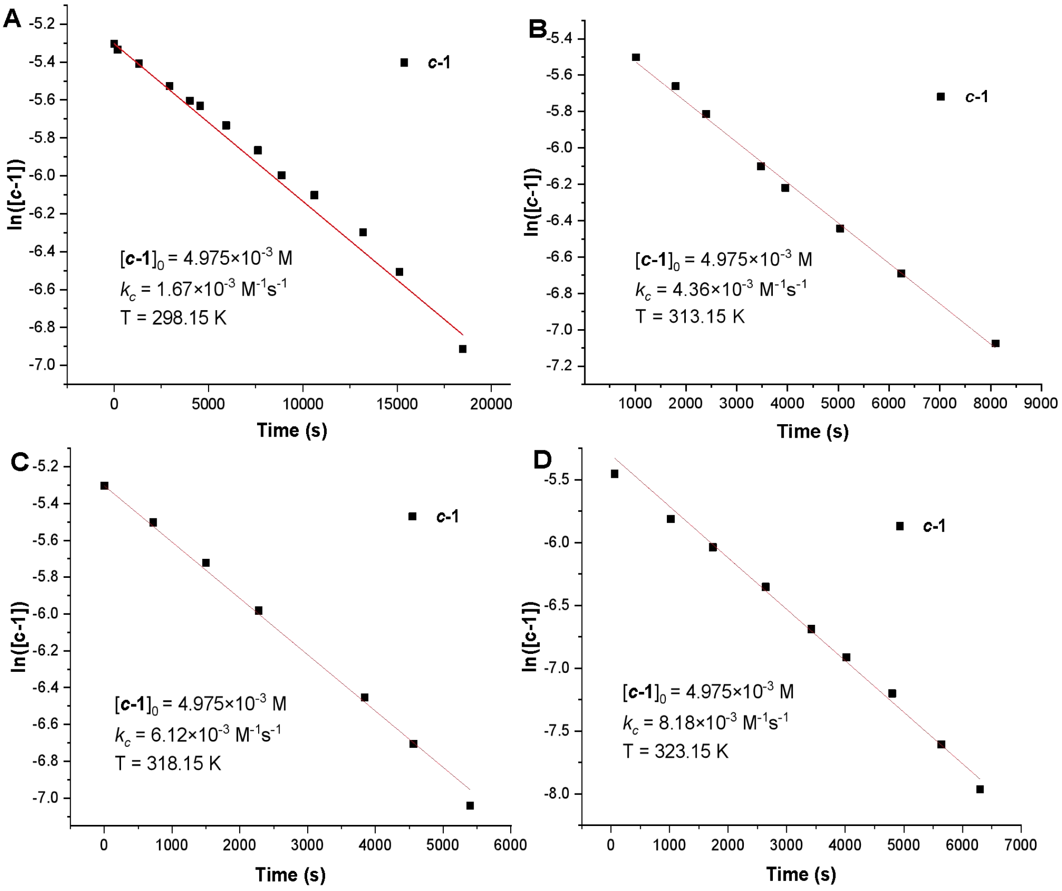

Supplementary Figure 106. Kinetic fitting curves of the reactions of ***c*-1** with 1-propanethiol (10 equiv.) at 298.15 K (A), 313.15 K (B), 318.15 K (C), and 323.15 K (D). The reaction rate was shown in the insert of the figure.

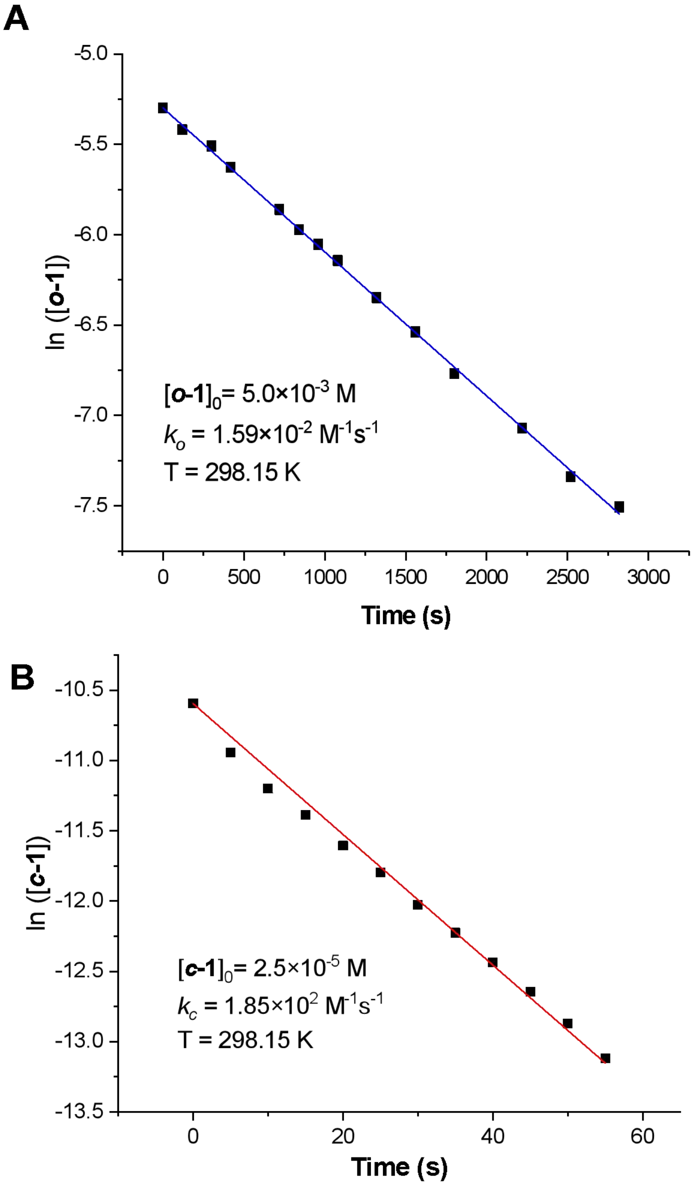

Supplementary Figure 107. Kinetic fitting curves of the reactions of ***o*-1** with aniline (10 equiv.) at 298.15 K (A) and ***c*-1** with aniline (10 equiv.) at 298.15 K (B). The ratio of k_c and k_o is 1.16 x 10^4.

7. Computations

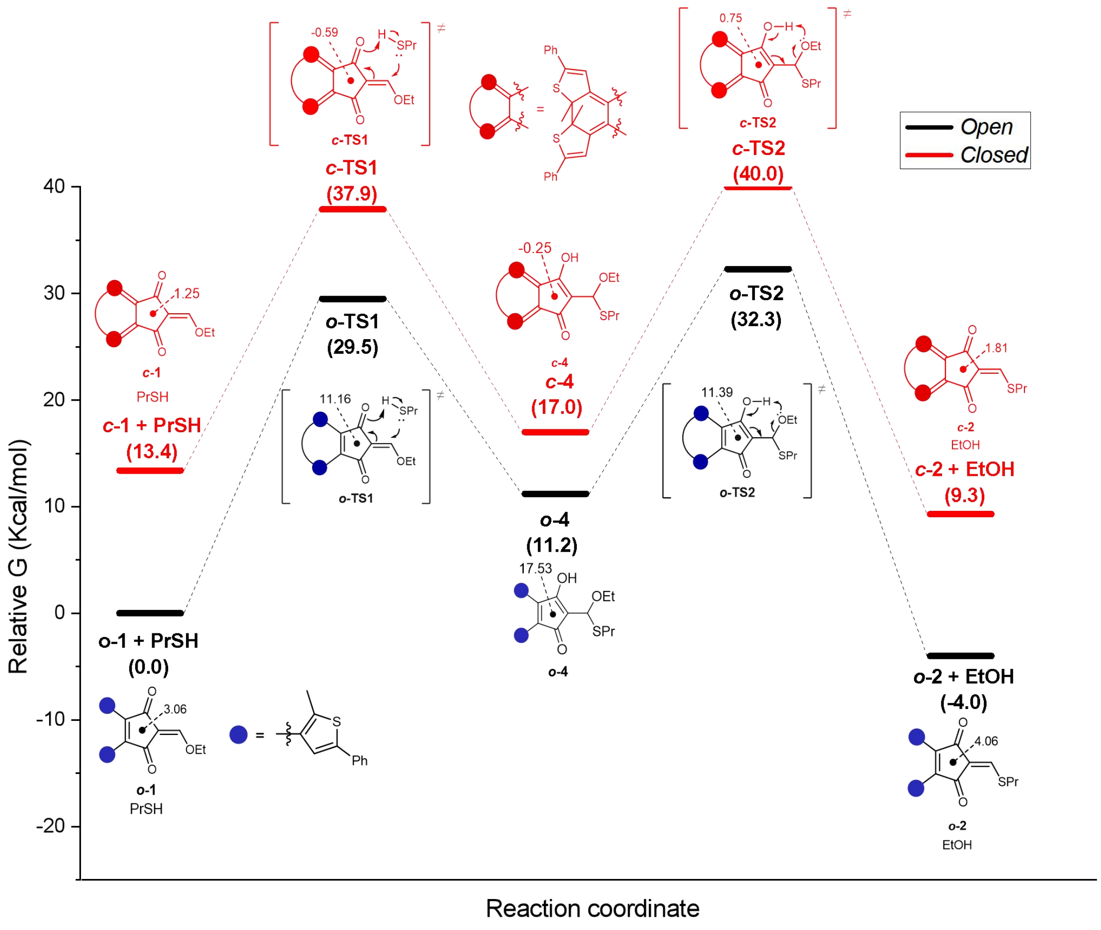

Supplementary Figure 108. Calculated reaction pathway between ***o*-1**/***c*-1** and 1-propanethiol, with relative free energy (kcal/mol) and NICS(1)$_{zz}$ (ppm) values listed.

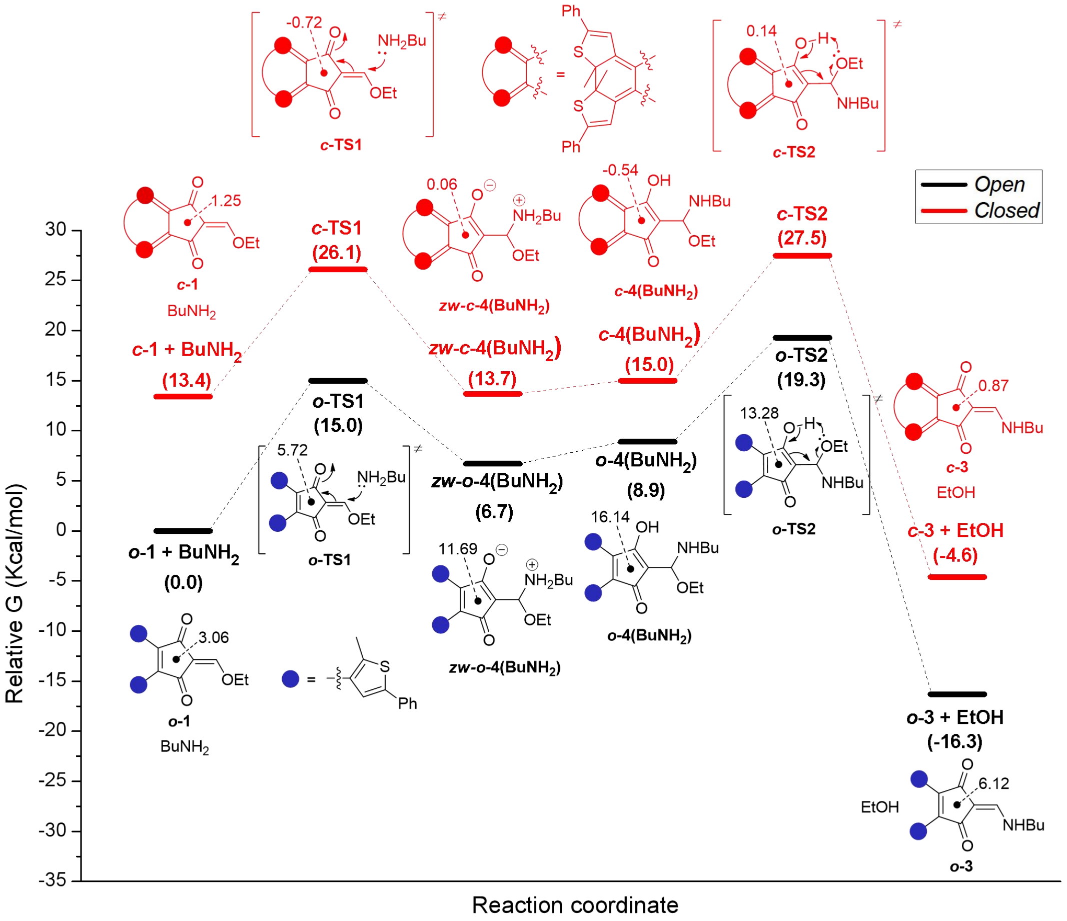

Supplementary Figure 109. Calculated reaction pathway between ***o*-1**/***c*-1** and 1-butylamine, with relative free energy (kcal/mol) and NICS(1)$_{zz}$ (ppm) values listed.

8. Light-Controlled Amphiphilic Assemblies

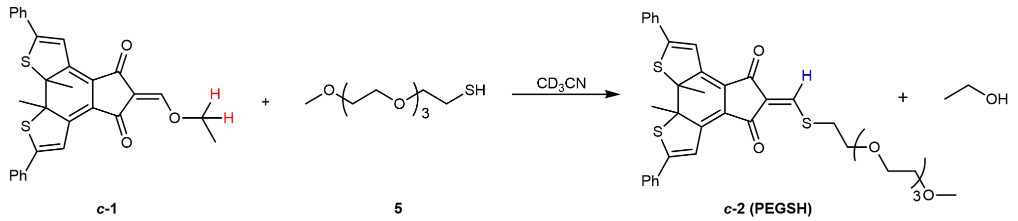

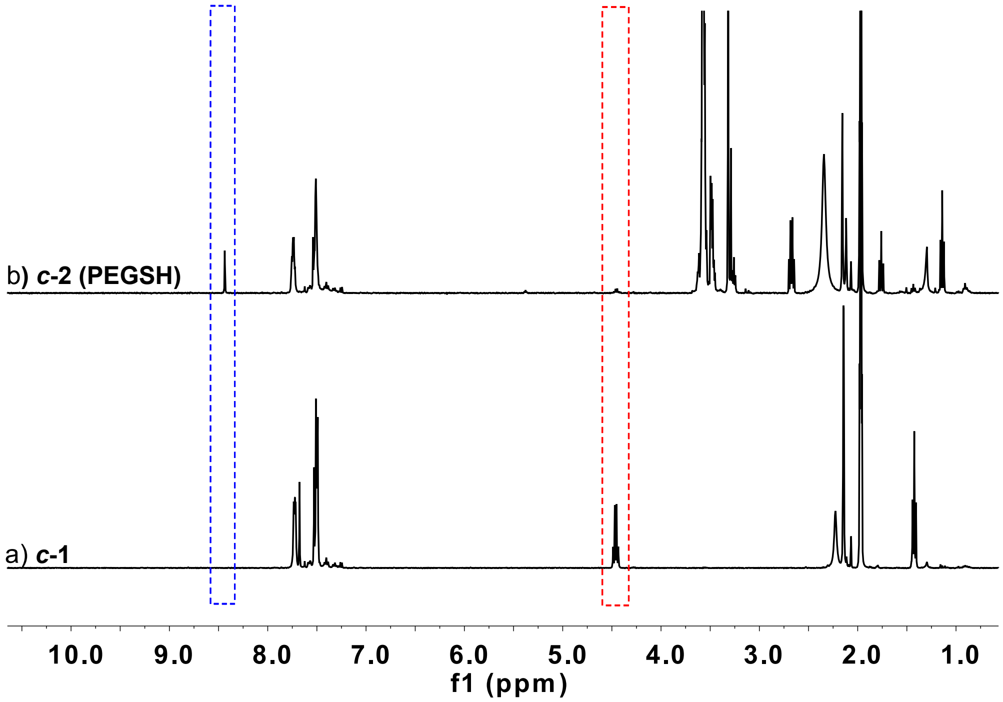

Supplementary Figure 110. (a) ^{1}H NMR (400 MHz, 20 °C) spectrum of ***c*-1** (5 mM) in CD_3CN (0.5 mL); (b) After addition of **5** (3 equiv.) into the panel a to afford ***c*-2** (PEGSH) after 12 h.

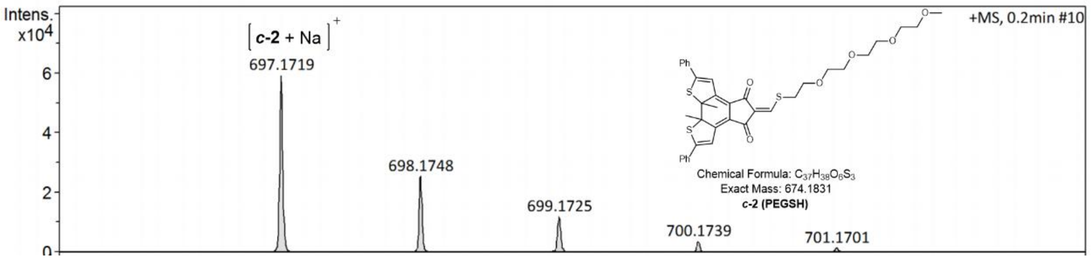

Supplementary Figure 111. ESI mass spectrum of ***c*-2** (PEGSH) in acetonitrile.

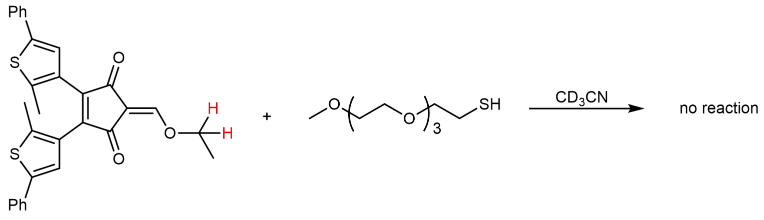

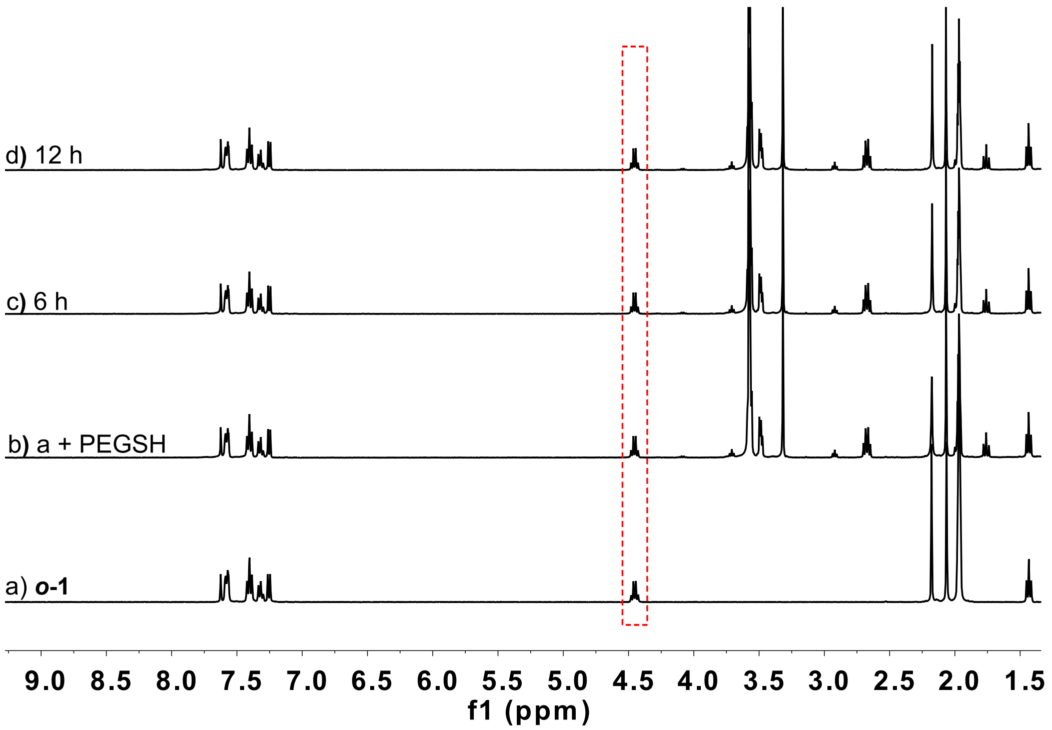

Supplementary Figure 112. Stacked ^{1}H NMR (400 MHz, 20 °C) spectra of the reaction of ***o*-1** (5 mM) with **5** (3 equiv.) in CD_3CN in 12 h. No reaction occurred.

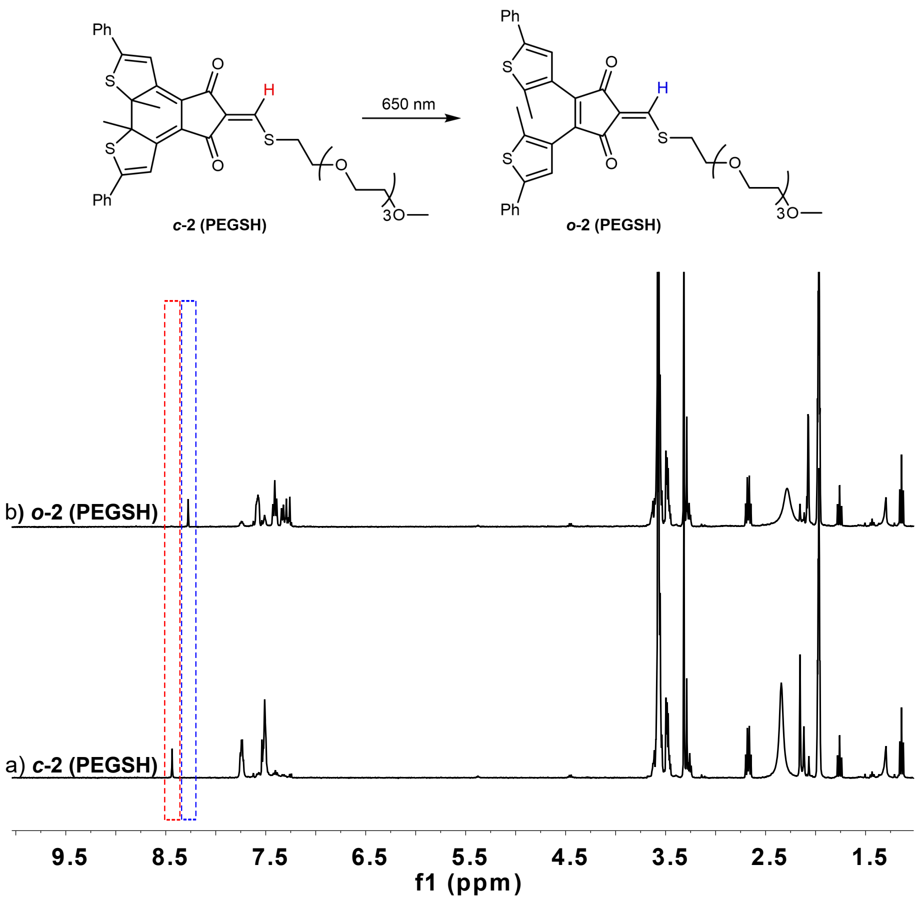

Supplementary Figure 113. (a) ^{1}H NMR (400 MHz, 20 °C) spectrum of ***c*-2** (5 mM) created *in situ* in CD3CN; (b) After illumination with visible light (650 nm, 2 h) to convert ***c*-2** into ***o*-2**. The ratio of ***o*-2** and ***c*-2** is 95:5.

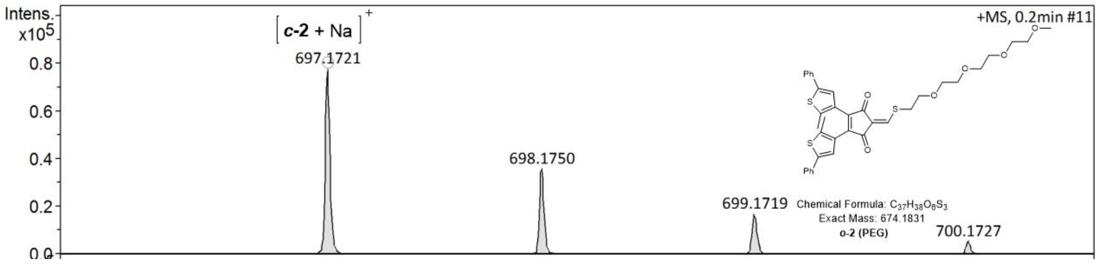

Supplementary Figure 114. ESI mass spectrum of ***o*-2** (PEGSH) in acetonitrile.

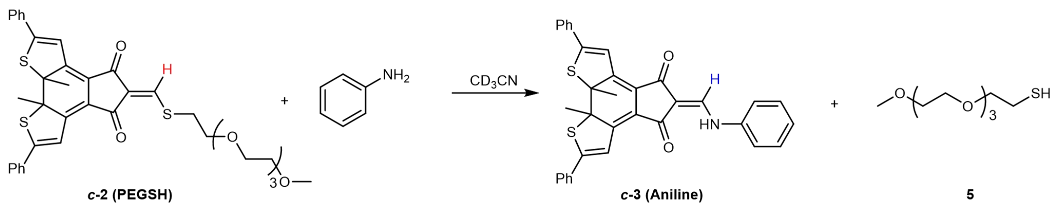

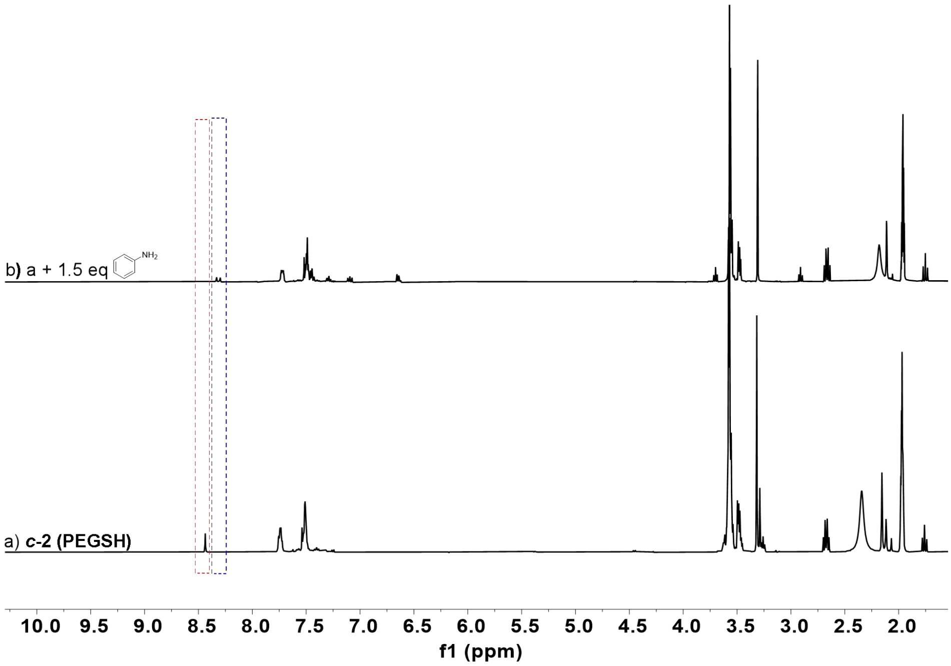

Supplementary Figure 115. (a) ^{1}H NMR (400 MHz, 20 °C) spectrum of ***c*-2** (5 mM) created *in situ* in CD_3CN; (b) After addition of aniline (1.5 equiv.) into the panel a to afford ***c*-3**. ***c*-2** completely converted to ***c*-3** after 10 min.

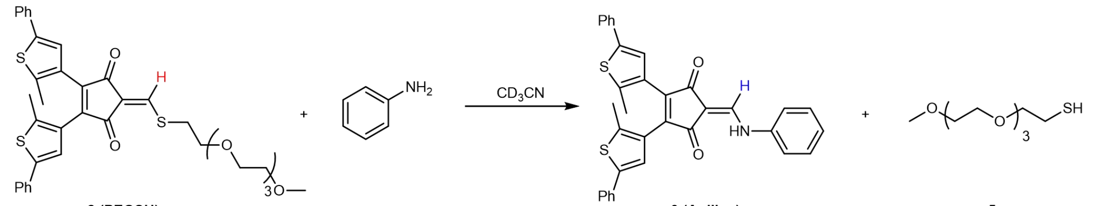

***o*-2 (PEGSH)** ***o*-3 (Aniline)** **5**

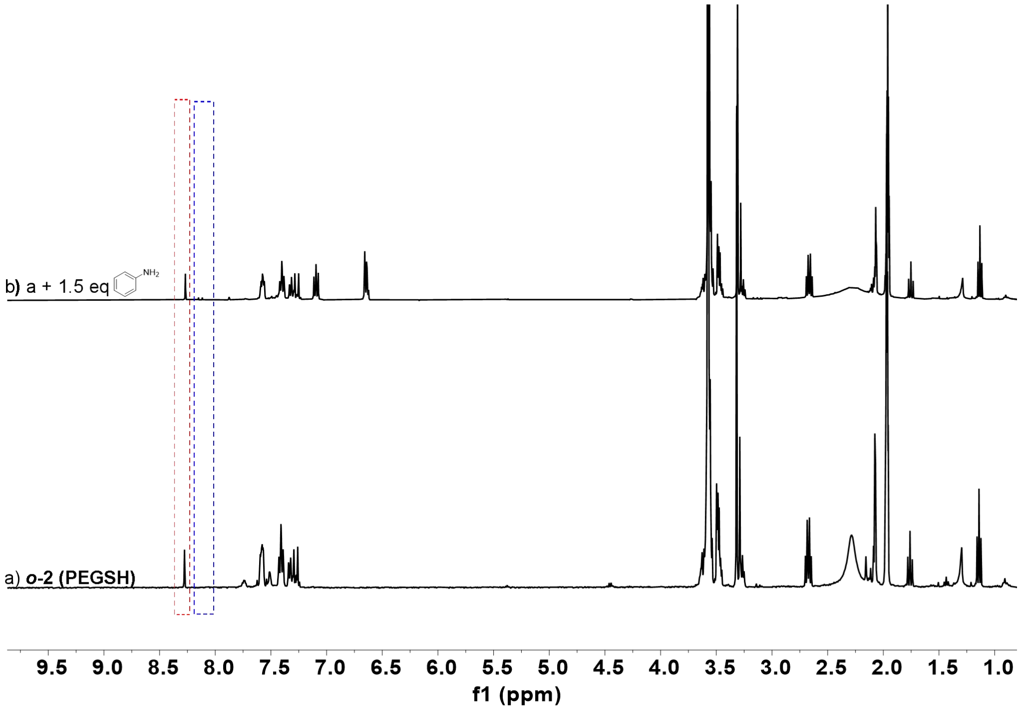

Supplementary Figure 116. (a) ^{1}H NMR (400 MHz, 20 °C) spectrum of ***o*-2** (5 mM) created *in situ* in CD_3CN; (b) After addition of aniline (1.5 equiv.) into the panel a to afford ***o*-3**. The yield of ***o*-3** is 15% after 24 h.

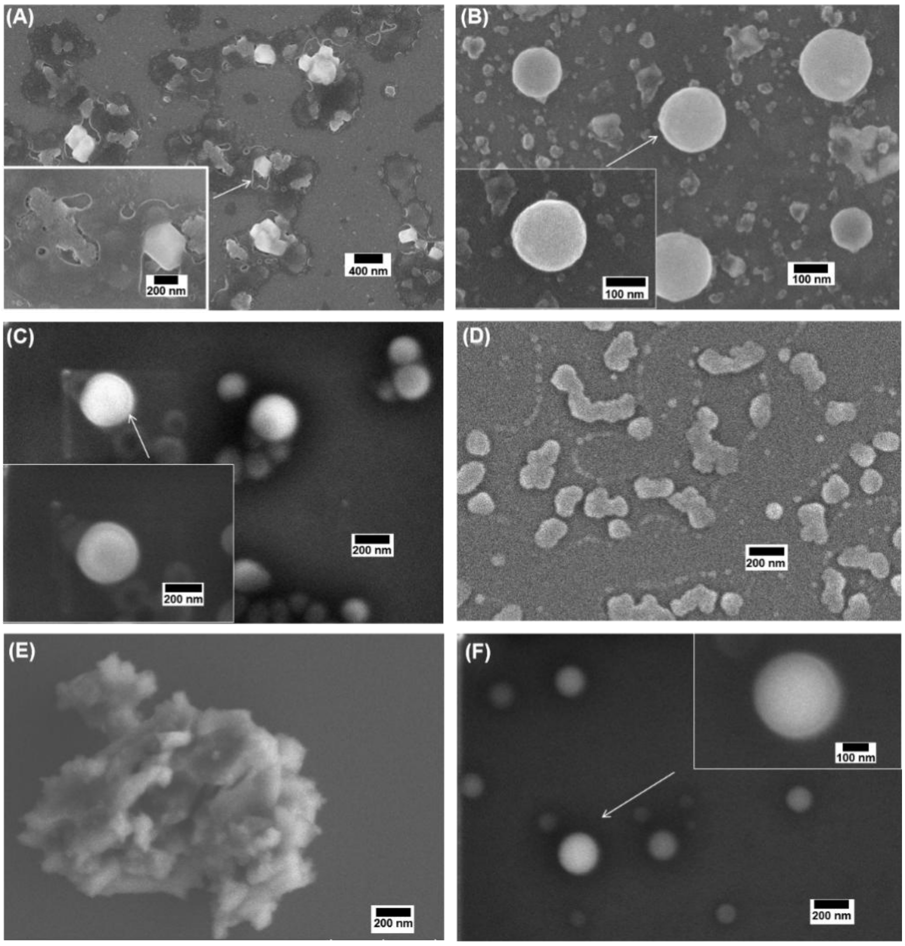

Supplementary Figure 117. SEM images of the amphiphilic assemblies formed under the following conditions. (A) The reaction of ***o*-1** (5 mM) with **5** (3.0 equiv.) for 12 h. (B) Preparation of ***c*-2** from ***c*-1**. (C) ***o*-2** was obtained from ***c*-2** after illumination at 650 nm for 2 h. (D) The control compound **5**. (E) The reaction of ***c*-2** with aniline (1.5 equiv.) for 3 min. (F) The reaction of ***o*-2** with aniline (1.5 equiv.) for 24 h.

9. Light-Control Regulation of Polymers

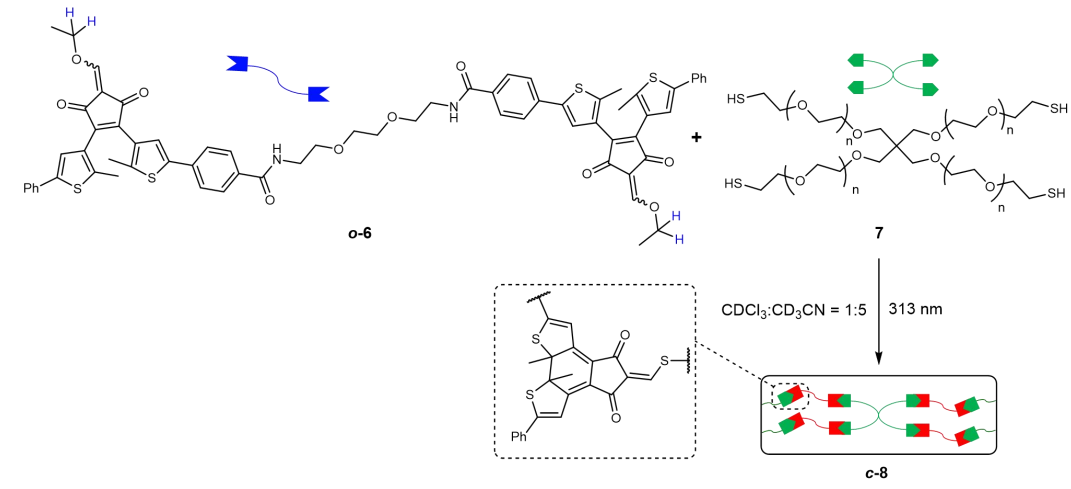

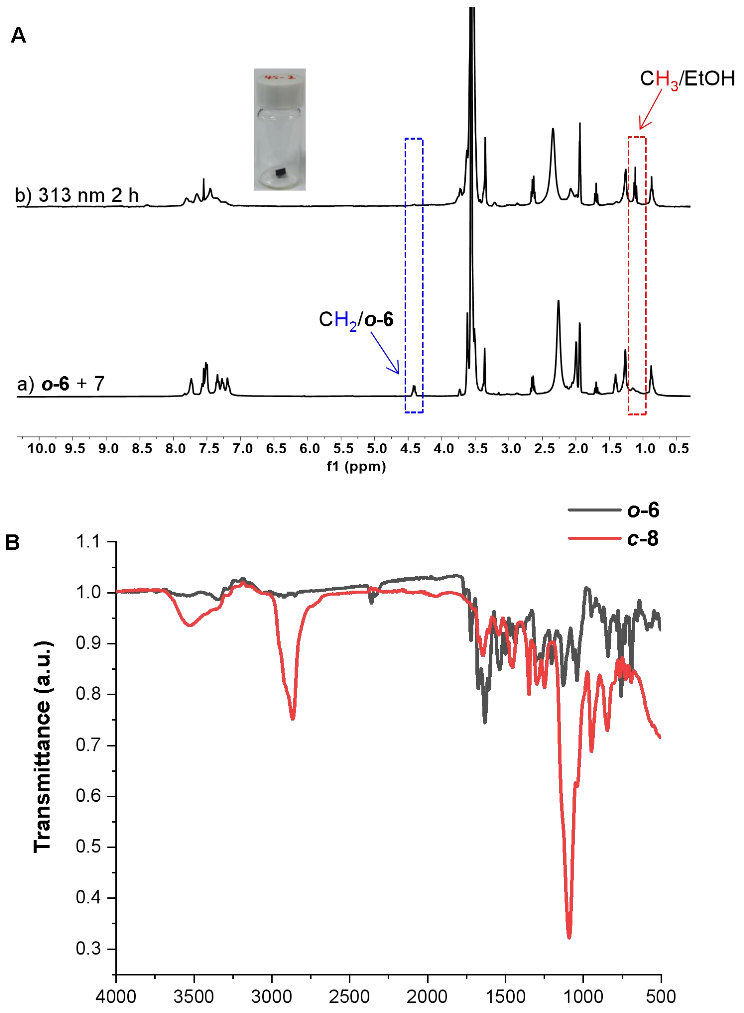

Supplementary Figure 118. (A) (a) ^{1}H NMR (400 MHz, 20 °C) spectrum of ***o*-6** (5 mM) with 4-arm-PEG-SH **7** (1.5 equiv.) in $CD_3CN:CDCl_3$ (5:1, 0.5 mL); (b) The illumination with UV light (313 nm, 2 h) to convert ***o*-6** into polymer ***c*-8**. (B) FTIR spectra of ***o*-6** and polymer ***c*-8**.

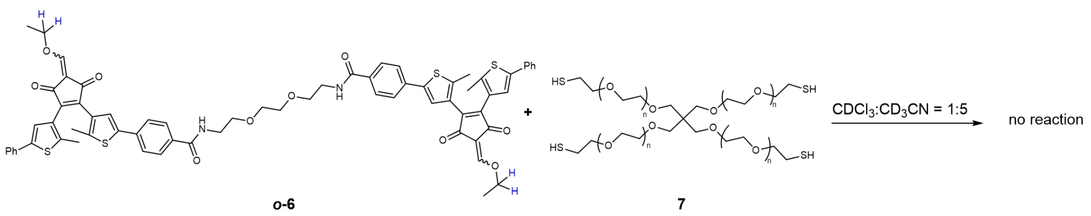

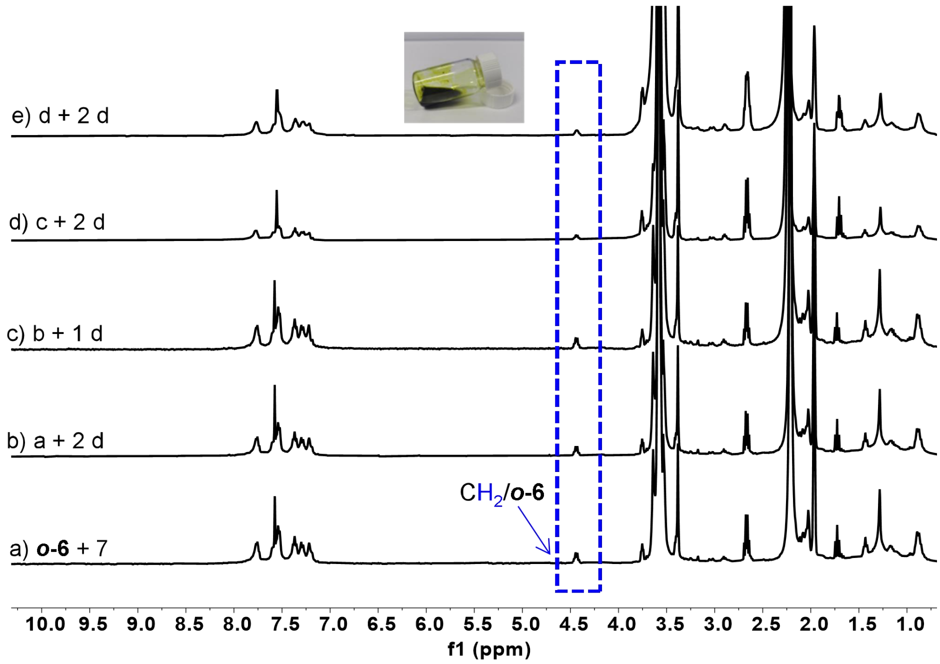

Supplementary Figure 119. Stacked ^{1}H NMR (400 MHz, 20 °C) spectra of the reaction of ***o*-6** (5 mM) with 4-arm-PEG-SH **7** (1.5 equiv.) in CD_3CN:$CDCl_3$ (5:1, 0.5 ml) at varied time. No detected of polymer ***o*-8** after 7 days.

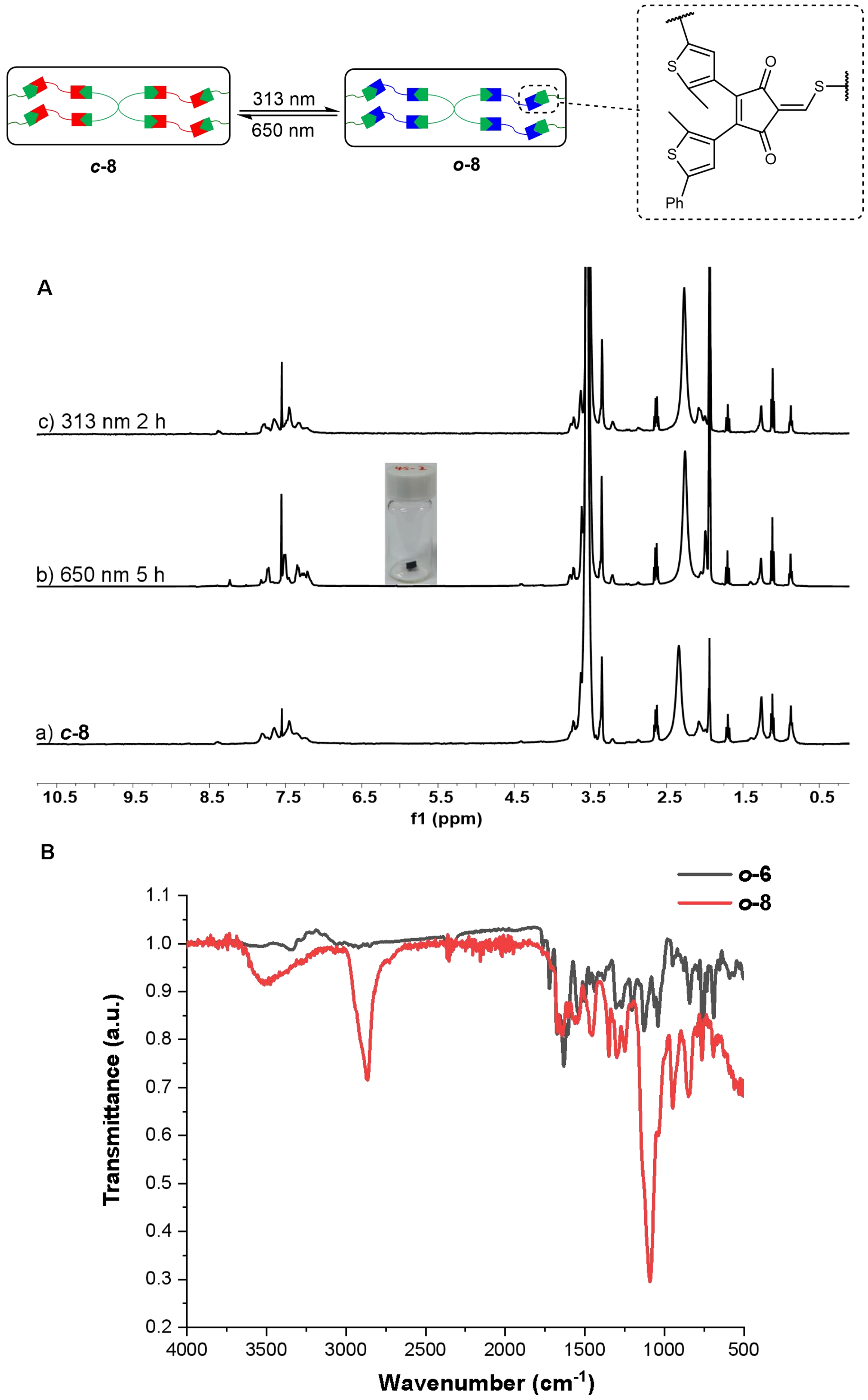

Supplementary Figure 120. (A) (a) ^{1}H NMR (400 MHz, 20 °C) spectrum of ***c*-8** created from ***o*-6** (5 mM) and 4-arm-PEG-SH **7** (1.5 equiv.) *in situ* in CD_3CN:$CDCl_3$ (5:1, 0.5 mL); (b) Irradiation of ***c*-8** with visible light (650 nm, 5 h) to give ***o*-8**; (c) Further irradiation with UV light (313 nm, 2 h) to recover ***c*-8**. (B) FTIR spectra of ***o*-6** and polymer ***o*-8**.

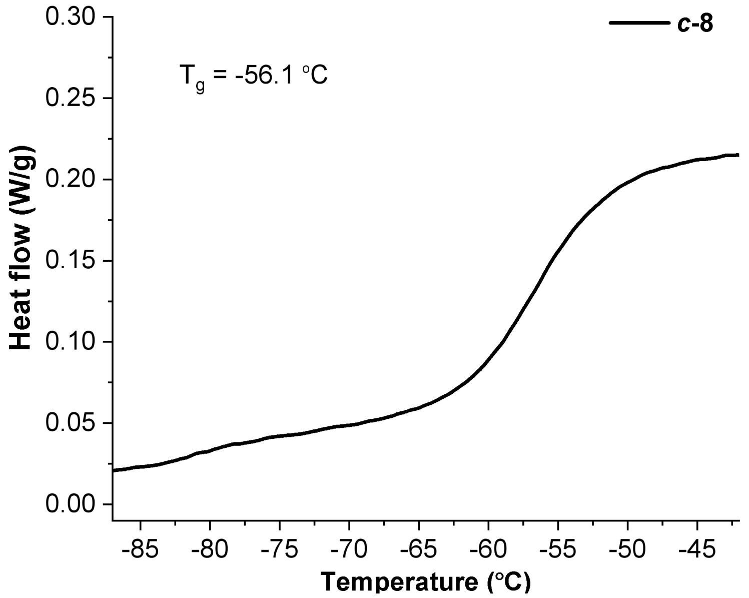

Supplementary Figure 121. Differential scanning calorimetry (DSC) thermogram for polymer ***c*-8**. The glass transition temperature was found to be -56.1 °C.

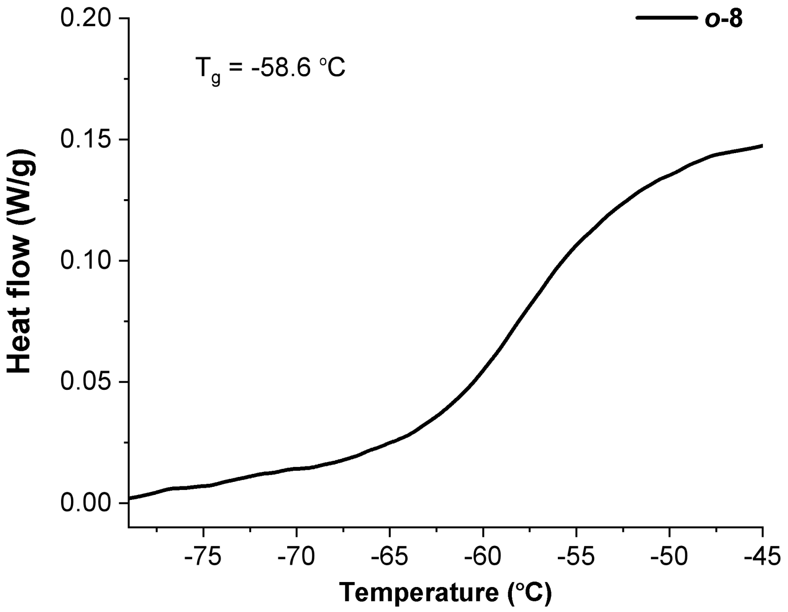

Supplementary Figure 122. Differential scanning calorimetry (DSC) thermogram for polymer ***o*-8**. The glass transition temperature was found to be -58.6 °C.

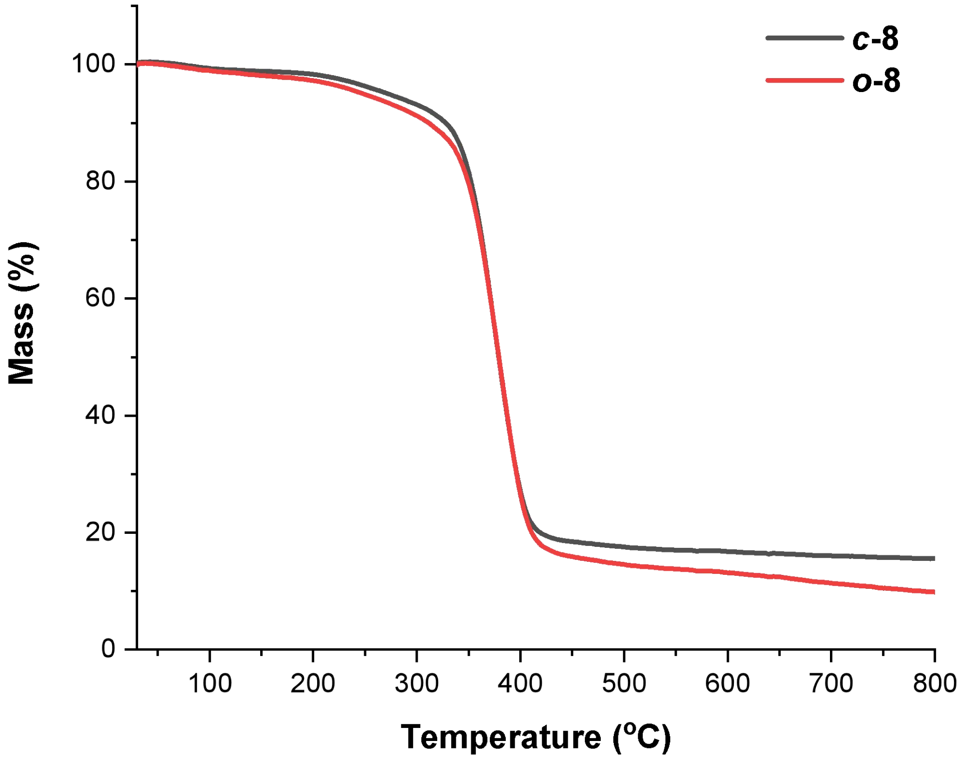

Supplementary Figure 123. Thermogravimetric analysis (TGA) curves recorded in nitrogen atmosphere for ***c*-8** and ***o*-8**.

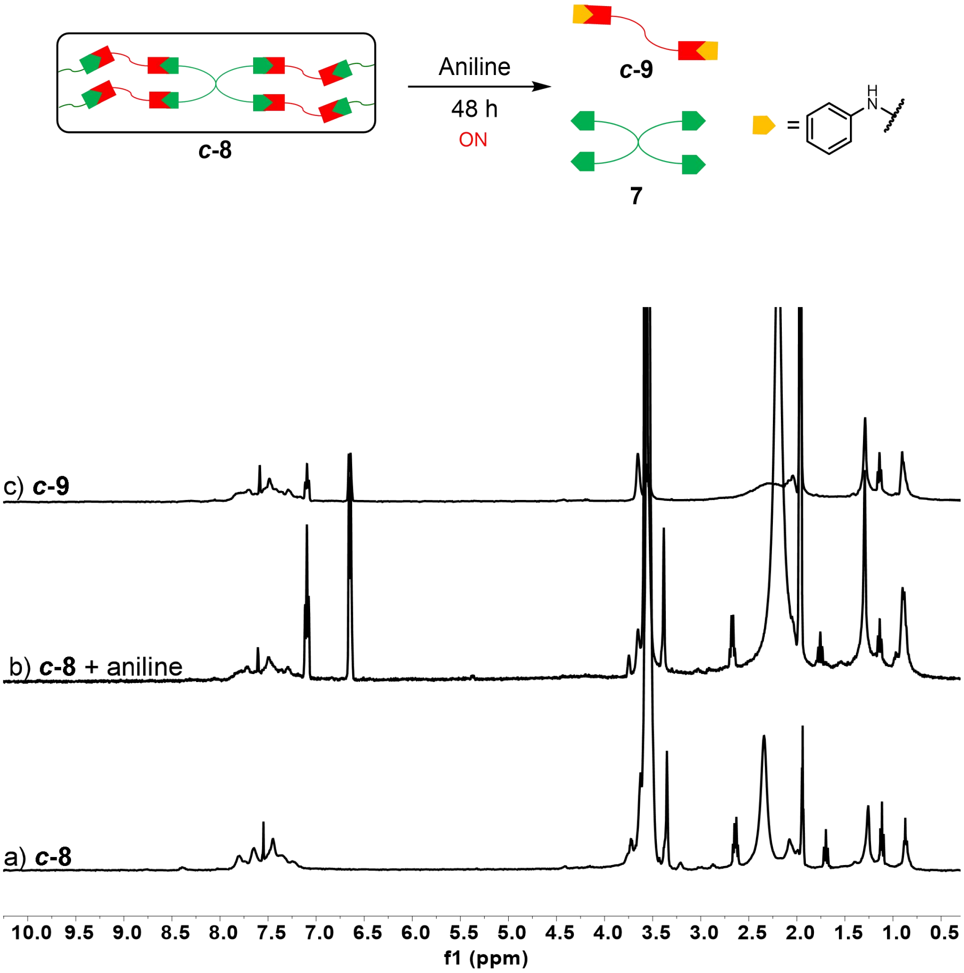

Supplementary Figure 124. (a) ^{1}H NMR (400 MHz, 20 °C) spectrum of ***c*-8** (5 mM) created *in situ* in CD_3CN; (b) After addition of aniline (20 mM) into the panel a to afford ***c*-9** in 48 h; (c) ^{1}H NMR spectrum of ***c*-9** created *in situ* from ***o*-6** (5 mM) and aniline (1.5 equiv.) in CD_3CN (0.5 mL) after illumination at 313 nm for 1.5 h. ***c*-8** degraded to create ***c*-9** after 48 h.

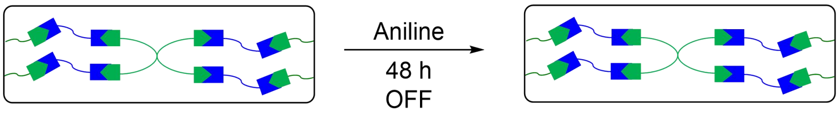

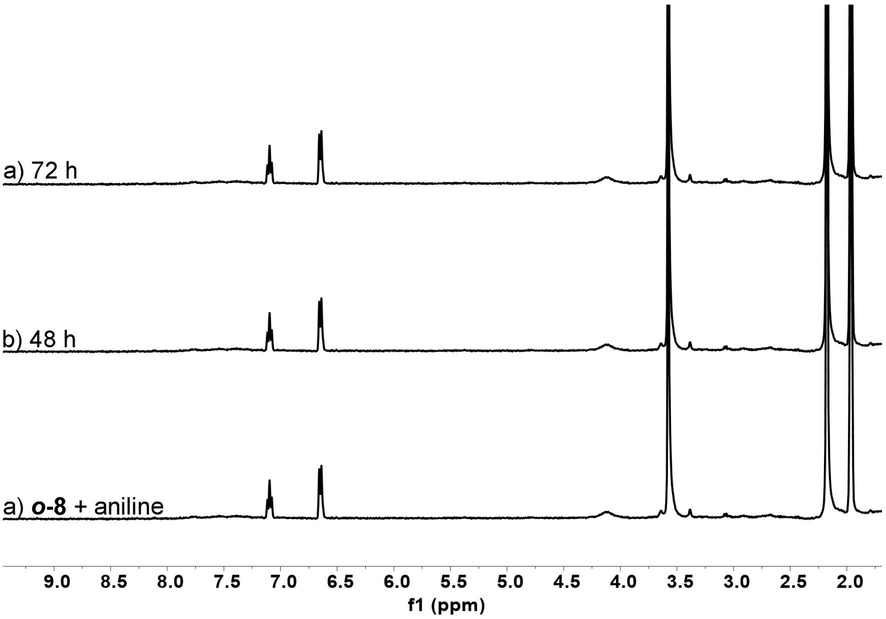

Supplementary Figure 125. Stacked ^{1}H NMR (400 MHz, 20 °C) spectra of the reaction of ***o*-8** (5 mM) with aniline (5 mM) CD_3CN (0.5 mL) at varied time. The insoluble polymer ***c*-8** remained after 48 h. Aniline peaks were observed in NMR.

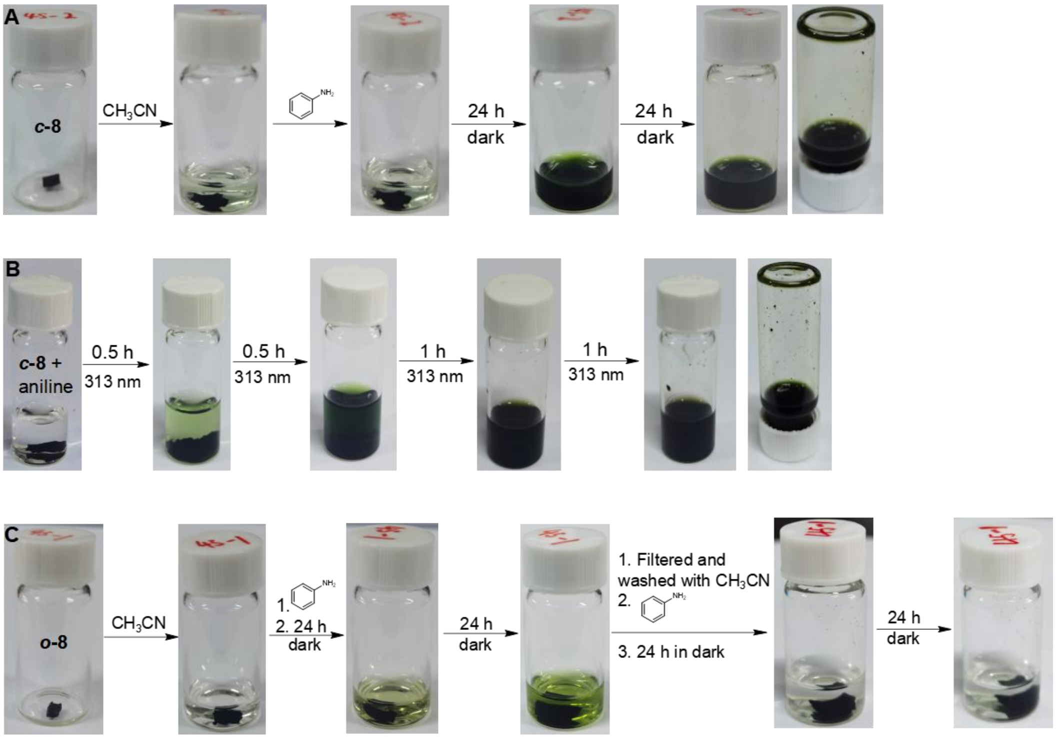

Supplementary Figure 126. Control of the degradation of polymers ***c*-8** and ***o*-8** with aniline. ***c*-8** (10 mg; M_n = 107364, M_w = 208222, PDI = 1.94) degraded in the solution of aniline (20 mM) in CH_3CN (1 mL) after 48 h to give ***c*-9** and release 4-arm-PEG-SH **7** (M_n = 15832, M_w = 21078, PDI = 1.33) (A). The UV light (313 nm) promoted the degradation of ***c*-8** after 3 h (B). The insoluble ***o*-8** remained and became swollen in the solution of aniline (20 mM) after 48 h (C). The coloration of the solution was caused by the incomplete photoconversion of ***c*-8** to ***o*-8**. After the solid was filtered and washed with CH_3CN followed by adding a new batch of aniline solution, the solution remained colorless after another 48 h.

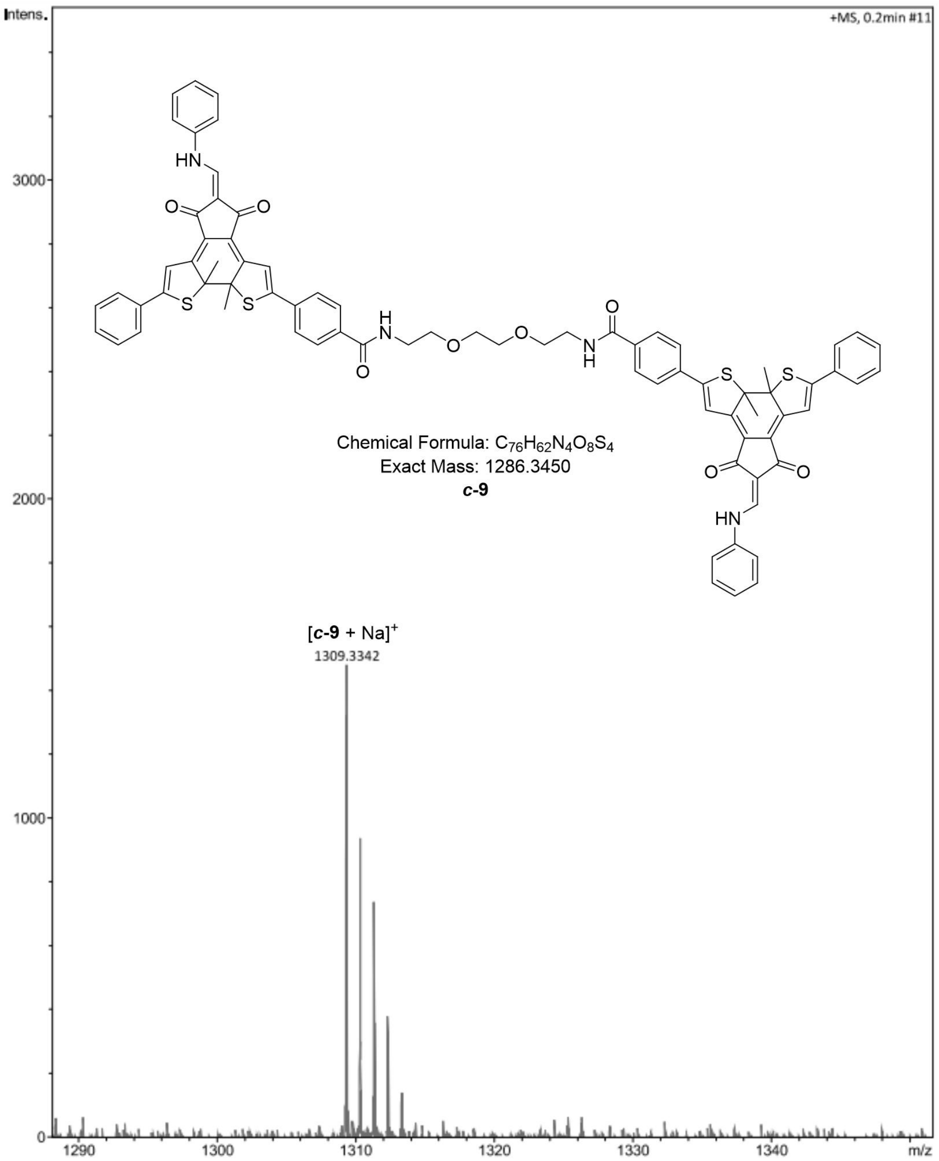

Supplementary Figure 127. ESI mass spectrum of the degradation of ***c*-8** to give ***c*-9**.

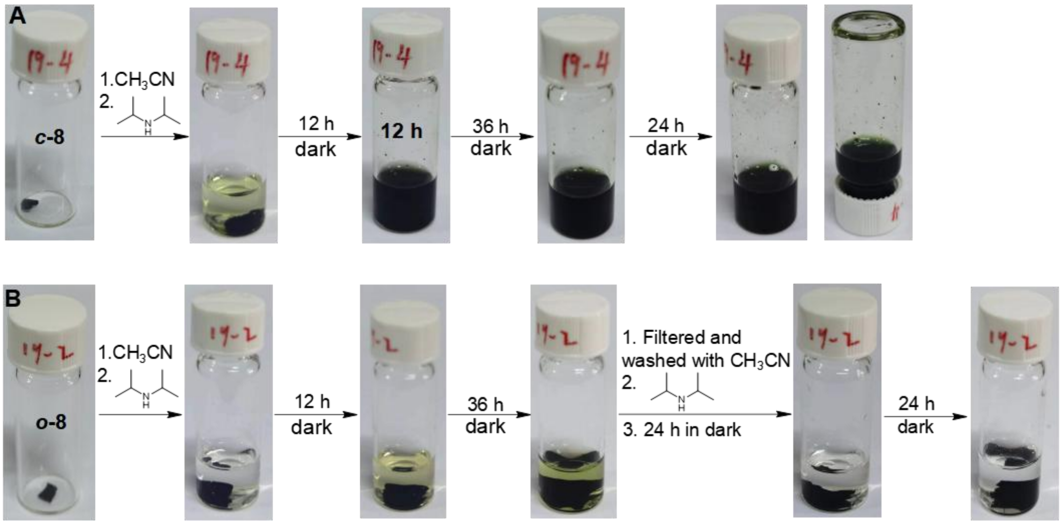

Supplementary Figure 128. Control of the degradation of polymers ***c*-8** and ***o*-8** with diisopropylamine. ***c*-8** (10 mg) degraded in the solution of diisopropylamine (20 mM) in CH_3CN (1 mL) after 72 h (A), while insoluble ***o*-8** remained and became swollen under the same condition after 48 h (B).

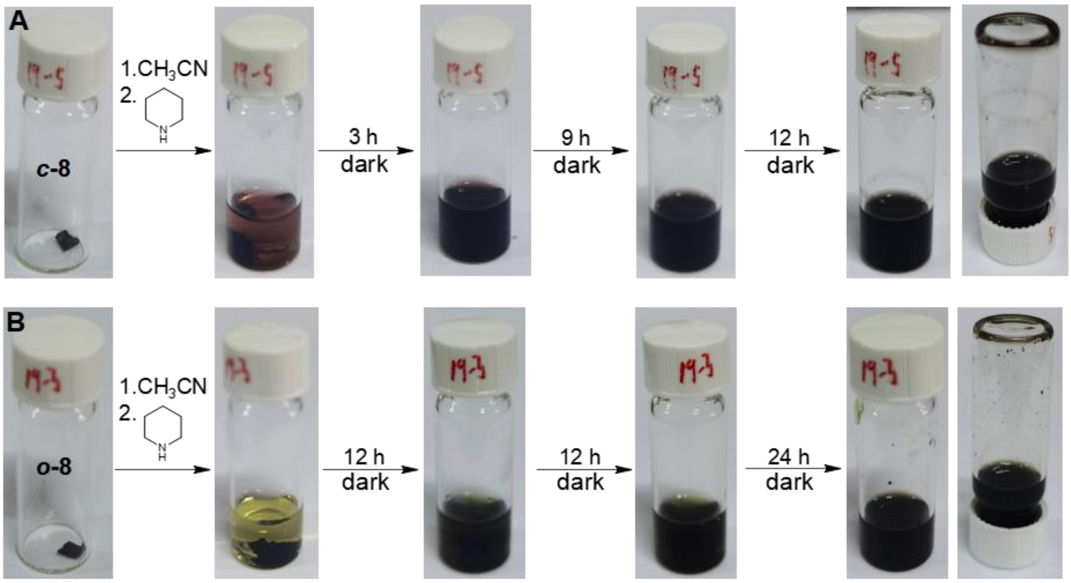

Supplementary Figure 129. Control of the degradation of polymers ***c*-8** and ***o*-8** with piperidine. ***c*-8** (10 mg) degraded in the solution of piperidine (20 mM) in CH_3CN (1 mL) after 24 h (A), while ***o*-8** degraded and dissolved under the same condition after 48 h (B).

10. Supplementary References

1. Nourmohammadian, F., Wu, T. & Branda, N. R. A 'chemically-gated' photoresponsive compound as a visible detector for organophosphorus nerve agents. *Chem. Commun.* **47**, 10954-10956 (2011).
2. Moreno, J., Schweighofer, F., Wachtveitl, J. & Hecht, S., Reversible photomodulation of electronic communication in a π-conjugated photoswitch-fluorophore molecular dyad. *Chem.-Eur. J.* **22**, 1070-1075 (2016).
3. Kuhni, J., Adamo, V. & Belser, P. Synthesis of an unsymmetrically substituted, dithienylethene-containing 1,10-phenanthroline ligand and its ruthenium (II) complex. *Synthesis* 1946-1948 (2006).
4. Choi, D. S. et al. Revised structures of N-substituted dibrominated pyrrole derivatives and their polymeric products. termaleimide models with low optical band gaps. *J. Org. Chem.* **63**, 2646−2655 (1998).
5. Delahaye, M.; Winne, J. M. & Du Prez, F. E. Internal Catalysis in Covalent Adaptable Networks: Phthalate Monoester Transesterification As a Versatile Dynamic Cross-Linking Chemistry. *J. Am. Chem. Soc.* **141**, 15277-15287 (2019).
