# Supplementary material for: Photoswitchable dynamic conjugate addition-elimination reactions as a tool for light-mediated click and clip chemistry
Source: Nat Commun. 2023 Jul 7;14:4015. doi: 10.1038/s41467-023-39669-x (PMC10328932; doi:10.1038/s41467-023-39669-x)
Supplement: Supplementary file 3 — Description of Additional Supplementary Files [file 41467_2023_39669_MOESM3_ESM.docx]

**Description of Additional Supplementary Files**

**File name: Supplementary Data 1**

**Description:** Atomic coordinates of the optimized geometries for the studied systems.

**File name: Supplementary Data 2**

**Description:** The single-crystal data of ***o*-3** are summarized in Supplementary Data 1 and archived at the Cambridge Crystallographic Data Centre under the reference number CCDC-2179459.
